# Timer-based proteomic profiling of the ubiquitin-proteasome system reveals a substrate receptor of the GID ubiquitin ligase

**DOI:** 10.1016/j.molcel.2021.04.018

**Published:** 2021-06-03

**Authors:** Ka-Yiu Edwin Kong, Bernd Fischer, Matthias Meurer, Ilia Kats, Zhaoyan Li, Frank Rühle, Joseph D. Barry, Daniel Kirrmaier, Veronika Chevyreva, Bryan-Joseph San Luis, Michael Costanzo, Wolfgang Huber, Brenda J. Andrews, Charles Boone, Michael Knop, Anton Khmelinskii

**Affiliations:** 1 Institute of Molecular Biology (IMB), Mainz, Germany; 2 Computational Genome Biology, German Cancer Research Center (DKFZ), Heidelberg, Germany; 3 Center for Molecular Biology of Heidelberg University (ZMBH), DKFZ-ZMBH Alliance, Heidelberg, Germany; 4 Genome Biology Unit, European Molecular Biology Laboratory (EMBL), Heidelberg, Germany; 5 The Donnelly Centre for Cellular and Biomolecular Research, University of Toronto, Toronto, ON, Canada; 6 Department of Molecular Genetics, University of Toronto, Toronto, ON, Canada; 7 Cell Morphogenesis and Signal Transduction, German Cancer Research Center (DKFZ), DKFZ-ZMBH Alliance, Heidelberg, Germany

**Keywords:** selective protein degradation, ubiquitin-proteasome system, proteostasis, protein quality control, fluorescent timers, N-degron pathways, GID ubiquitin ligase

## Abstract

Selective protein degradation by the ubiquitin-proteasome system (UPS) is involved in all cellular processes. However, the substrates and specificity of most UPS components are not well understood. Here we systematically characterized the UPS in *Saccharomyces cerevisiae*. Using fluorescent timers, we determined how loss of individual UPS components affects yeast proteome turnover, detecting phenotypes for 76% of E2, E3, and deubiquitinating enzymes. We exploit this dataset to gain insights into N-degron pathways, which target proteins carrying N-terminal degradation signals. We implicate Ubr1, an E3 of the Arg/N-degron pathway, in targeting mitochondrial proteins processed by the mitochondrial inner membrane protease. Moreover, we identify Ylr149c/Gid11 as a substrate receptor of the glucose-induced degradation-deficient (GID) complex, an E3 of the Pro/N-degron pathway. Our results suggest that Gid11 recognizes proteins with N-terminal threonines, expanding the specificity of the GID complex. This resource of potential substrates and relationships between UPS components enables exploring functions of selective protein degradation.

## Highlights

• Global analysis of proteome turnover in budding yeast
• Proteome turnover profiles for most components of the ubiquitin-proteasome system
• Substrates and functions for Ubr1 and GID, two E3s of the N-degron pathways
• Identification of a GID receptor for substrates with a threonine N terminus

## Graphical abstract

**Figure:**
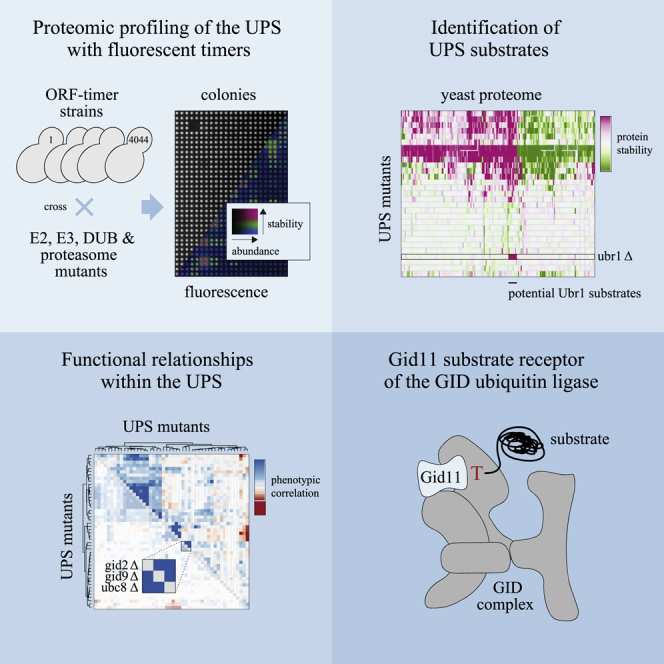

## Teaser

The ubiquitin-proteasome system (UPS) is key for selective protein degradation in eukaryotes. To understand its specificity, Kong et al. systematically analyze the effect of UPS mutants on yeast proteome turnover and explore the resulting resource of potential substrates and relationships between UPS components to gain insights into N-degron pathways.

## Introduction

The functional state of a cell is ultimately defined by the state of its proteome; i.e., the abundance, localization, turnover, and mobility of all proteins and their organization in complexes and organelles. Proteome integrity is maintained by a complex proteostasis network that regulates protein synthesis, folding, transport, and degradation (Balch et al., 2008; Balchin et al., 2016; Wolff et al., 2014). Numerous protein quality control systems operate throughout the protein life cycle and contribute to proteome homeostasis by preventing, detecting, and removing abnormal proteins. In eukaryotes, the ubiquitin-proteasome system (UPS) plays a key role in selective protein degradation, where a cascade of ubiquitin-activating (E1), ubiquitin-conjugating (E2), and ubiquitin-protein ligase (E3) enzymes marks proteins with ubiquitin. The functional outcome of ubiquitination (e.g., proteasomal or lysosomal degradation, change in localization, or protein-protein interactions) depends on the type of modification (mono- versus polyubiquitination) and the type of polyubiquitin chains (Hershko and Ciechanover, 1998; Kleiger and Mayor, 2014; Zheng and Shabek, 2017). Deubiquitinating enzymes (DUBs), which remove ubiquitin from target proteins and replenish the pool of free ubiquitin, are involved at various stages of the targeting and degradation processes (Mevissen and Komander, 2017; Oh et al., 2018).

Specificity in the UPS is provided by E3s. The human genome encodes more than 600 E3s, and even a simple organism, such as the budding yeast *S. cerevisiae*, has ∼100 E3s, which are thought to recognize and ubiquitinate distinct sets of proteins (Finley et al., 2012; Pickart, 2001; Zheng and Shabek, 2017). Substrate recognition appears to involve E3 binding, directly or via cofactors, to short linear motifs in the substrate, known as degradation signals or degrons if ubiquitination leads to degradation (Ella et al., 2019; Ravid and Hochstrasser, 2008; Zheng and Shabek, 2017). A prominent class of degrons is located at protein N termini. These N-degrons, defined by the first few N-terminal residues, are recognized by different E3s called N-recognins (Varshavsky, 2011, 2019).

In the Arg/N-degron pathway, the budding yeast E3 Ubr1 recognizes and targets for proteasomal degradation proteins with positively charged (R, K, H) or bulky hydrophobic (W, L, F, Y, I) N-terminal residues (Bachmair and Varshavsky, 1989; Bachmair et al., 1986). In addition, proteins with N-terminal N or Q residues can be processed by the N-terminal amidase Nta1, yielding N termini with D or E residues, followed by their arginylation by the N-terminal arginyl-transferase Ate1, producing N termini that start with an arginine and are thus targets of Ubr1 (Baker and Varshavsky, 1995; Balzi et al., 1990). Ubr1 can also recognize N termini where the unacetylated initiator methionine is followed by a bulky hydrophobic residue (Kim et al., 2014). Despite its well-established specificity, few substrates are known for the Arg/N-degron pathway because they are usually generated through endoproteolysis and, thus, difficult to identify (Varshavsky, 2011).

The GID (glucose-induced degradation-deficient) complex is a multisubunit E3 conserved in eukaryotes (Francis et al., 2013). In yeast, it functions as the N-recognin of the Pro/N-degron pathway, where it targets the gluconeogenic enzymes fructose-1,6-bisphosphatase Fbp1, malate dehydrogenase Mdh2, phosphoenolpyruvate carboxykinase Pck1, and isocitrate lyase Icl1 for proteasomal degradation when switching from ethanol to glucose as a carbon source (Chen et al., 2017; Hämmerle et al., 1998; Regelmann et al., 2003; Santt et al., 2008). These substrates carry N-degrons with a proline as the N-terminal or second residue for recognition by the receptor subunit Gid4 (Chen et al., 2017; Hämmerle et al., 1998). Gid4 expression is induced during the switch of carbon source, ensuring timely inactivation of gluconeogenesis (Menssen et al., 2018; Santt et al., 2008). The GID E3 likely has additional functions because the core complex is present under various conditions, and a second substrate receptor, Gid10, is induced by starvation or osmotic stress (Chen et al., 2017; Melnykov et al., 2019; Qiao et al., 2020).

Because of its major roles in recycling of unnecessary or abnormal proteins, the UPS is involved in essentially all cellular processes (Hershko and Ciechanover, 1998; Kleiger and Mayor, 2014; Zheng and Shabek, 2017). Failure in selective protein degradation and quality control are associated with various diseases, including cancer and neurodegenerative disorders. Moreover, proteome homeostasis declines with age (Balch et al., 2008; Balchin et al., 2016; Labbadia and Morimoto, 2015). Despite the central role of the UPS in cell physiology, the functions of many UPS components are unclear, and the substrate specificities of E3s and DUBs are not well defined.

**Figure 1:**
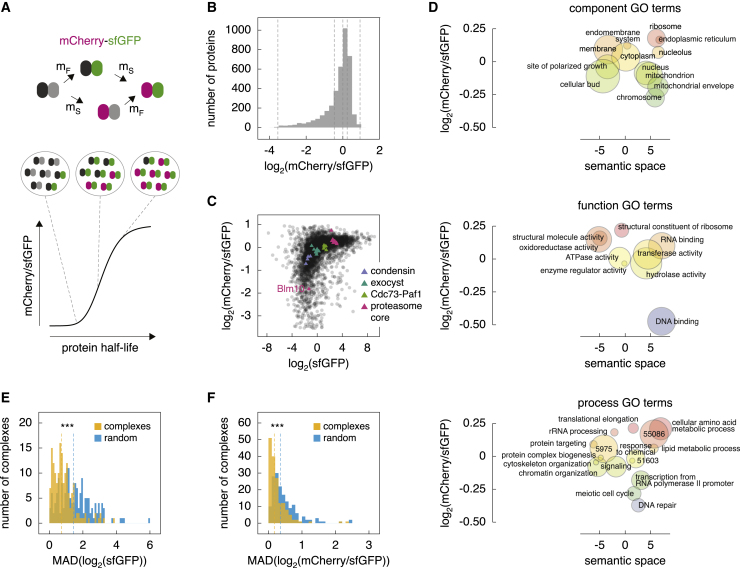
Turnover of the yeast proteome (A) Cartoon of the mCherry-sfGFP timer (top). Because of different maturation kinetics of mCherry (slow, m_S_) and sfGFP (fast, m_F_), the mCherry/sfGFP ratio reports the stability of timer-tagged proteins in the steady state (bottom). (B) Median-centered distribution of mCherry/sfGFP ratios in the tFT library, representing protein stability in the yeast proteome. Shown are fluorescence measurements of colonies, median of 2 biological replicates, each with 4 technical replicates per protein. Dashed lines, quantiles used in downstream analyses (Figures S1M and S1N). (C) Relationship between sfGFP intensities (protein abundance) and mCherry/sfGFP ratios in the tFT library. Example protein complexes are highlighted. (D) Median mCherry/sfGFP ratios of proteins in the tFT library mapped to Gene Ontology (GO) terms. GO term 5975, carbohydrate metabolic process; 51603, proteolysis involved in cellular protein catabolic process; 55086, nucleobase-containing small molecule metabolic process. Similar GO terms are closer in semantic space (Supek et al., 2011). (E and F) Distributions of median absolute deviations (MADs) of sfGFP intensities (E) or mCherry/sfGFP ratios (F) for complexes in the tFT library. Random samples of the proteome, drawn in sets of N (where N is the number of complex subunits), are shown for comparison (n = 100 random draws). Dashed lines, medians of the distributions. ^∗∗∗^p < 0.001 in a Wilcoxon rank-sum test. See also Figure S1 and Table S1.

Here we used budding yeast to systematically assess the role of UPS components in proteome homeostasis. Besides the ∼100 E3s and accessory subunits, the yeast UPS consists of a single E1, 11 E2s, 21 DUBs, the proteasome, and regulatory factors (Finley et al., 2012). We examined how inactivation of UPS components affects proteome abundance and turnover using tandem fluorescent protein timers (tFTs). A tFT is a tag composed of two fluorescent proteins with different kinetics of fluorophore maturation, such as mCherry and superfolder GFP (sfGFP) (Figure 1a). The mCherry/sfGFP ratio of fluorescence intensities is a measure of protein turnover in the steady state, increasing as a function of stability of the tFT-tagged protein (Figure 1A; Khmelinskii et al., 2012), whereas the sfGFP signal is a measure of protein abundance. Using a proteome-wide library of strains expressing tFT-tagged proteins (Khmelinskii et al., 2014), we profiled the yeast proteome in 132 mutants, including most E2, E3, and DUB enzymes. We exploit the resulting dataset to define functions for various UPS components and to gain insight into N-degron pathways.

## Results

### Yeast proteome turnover

We used the tFT library to characterize *S. cerevisiae* proteome abundance and turnover. The library consists of 4,044 strains, each with one open reading frame (ORF) tagged chromosomally with the mCherry-sfGFP timer (Khmelinskii et al., 2014; Figure 1A). We grew the library as an ordered array of colonies and measured their mCherry and sfGFP intensities (STAR Methods). sfGFP intensities provided reproducible estimates of protein abundance (Figures S1A and S1B; Table S1; Pearson correlation coefficient [r] = 0.99 between 2 library replicates), in line with independent measurements (Ghaemmaghami et al., 2003; de Godoy et al., 2008; Newman et al., 2006; Figures S1C–S1E). mCherry/sfGFP ratios provided reproducible estimates of protein turnover (Figure S1F; Table S1), although the associated error was higher because of combined uncertainties in measuring mCherry and sfGFP intensities (r = 0.89 between 2 replicates). The distribution of mCherry/sfGFP ratios was skewed toward unstable proteins (Figures 1B and 1C), suggesting that degradation is frequently used to tune protein abundance. This is consistent with observations in yeast and other organisms (Belle et al., 2006; Boisvert et al., 2012; Cambridge et al., 2011; Kristensen et al., 2013; Schwanhäusser et al., 2011), although we cannot exclude a contribution of the non-linear relationship between mCherry/sfGFP ratios and protein half-life to this trend (Figure S1G). The correlation between mCherry/sfGFP ratios and protein half-lives determined with cycloheximide chases of strains expressing proteins fused to a tandem affinity purification (TAP) tag (Belle et al., 2006) or pulse SILAC (stable isotope labeling by amino acids in cell culture) mass spectrometry (Christiano et al., 2014) was low (Figures S1H and S1I; r = 0.26 or 0.32). The correlation between the cycloheximide chase and pulse SILAC datasets was even lower (Figure S1J; r = 0.18). Multiple factors contribute to these discrepancies, including different growth conditions used in each study, the effect of cycloheximide on cell physiology, the potential effect of bulky tFT and TAP tags on protein turnover, and the inherent bias of mass spectrometry against low-abundance proteins.

Nevertheless, the distribution of mCherry/sfGFP ratios revealed global features of proteome turnover. First, mCherry/sfGFP ratios correlated with protein abundance so that low-abundance proteins were less stable (Figure 1C). Second, protein stability varied with protein localization and function. For instance, DNA binding proteins and proteins involved in cell cycle progression or localized to the mitotic spindle exhibited faster turnover, whereas abundant components of housekeeping machinery, such as ribosomes or metabolic enzymes, were stable (Figures 1D and S1K), consistent with previous observations (Belle et al., 2006; Boisvert et al., 2012; Cambridge et al., 2011; Kristensen et al., 2013; Schwanhäusser et al., 2011). Yet, mCherry/sfGFP ratios should be compared with caution between subcellular compartments because the tFT readout can be affected by the intracellular environment (Khmelinskii and Knop, 2014). For example, mCherry/sfGFP ratios of secretory proteins varied with location of their C termini (Kim et al., 2006; Figure S1L). Third, protein abundance and stability within protein complexes were more similar than expected at random (Figures 1E and 1F), highlighting that complex subunits are co-regulated and that assembly into a complex can stabilize individual subunits, equalizing their turnover (Dephoure et al., 2014; Li et al., 2014; McShane et al., 2016; Taggart and Li, 2018). Interestingly, some subunits exhibited clearly distinct and faster turnover (e.g., the proteasome activator Blm10 was less stable compared with other proteasomal subunits; Figure 1C), suggesting a regulatory role of protein turnover in complex assembly or function.

Finally, we searched for sequence features correlated with protein half-life. Unstable proteins were enriched in cysteine, serine, and asparagine but depleted of alanine, glycine, and valine residues (Figure S1M). These trends are reminiscent of those observed by Belle et al. (2006) and were present even after excluding disordered regions from the analysis (Figure S1N). However, their significance remains unclear. Protein stability was negatively correlated with the presence and number of long disordered regions (Figure S1O), which can increase the efficiency of proteasomal degradation by serving as initiation sites (van der Lee et al., 2014). These results demonstrate that abundance and turnover of the yeast proteome can be analyzed with the tFT library.

### Functional profiling of the UPS

**Figure 2:**
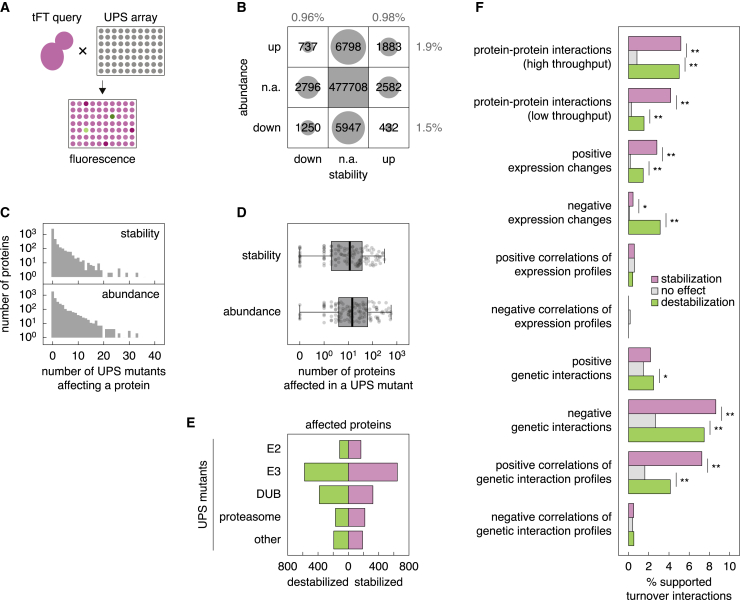
Influence of UPS components on proteome abundance and stability (A) Cartoon of screens to profile the yeast UPS. Each strain in the tFT library (tFT query) was crossed with an array of mutants in UPS components (UPS array), followed by mCherry and sfGFP fluorescence measurements of colonies. (B) Summary of phenotypic outcomes (changes of protein abundance and stability; n.a., not affected) across all tested mutant-tFT pairs at 1% FDR (false discovery rate). The percentage of mutant-tFT pairs with each phenotype is indicated. (C) Number of mutants affecting protein stability or abundance for the 3,806 tested tFT queries. Only significant interactions (1% FDR, absolute stability or abundance score > 4) were considered (C–E). (D) Number of proteins affected in terms of stability or abundance in 132 mutants in the UPS array. Centerlines mark the medians, box limits indicate the 25^th^ and 75^th^ percentiles, and whiskers extend to minimum and maximum values. (E) Number of proteins destabilized or stabilized in UPS mutants grouped by function (Table S2). (F) Overlap between turnover interactions, grouped according to change in protein stability at 1% FDR and external datasets. ^∗^p < 0.05 and ^∗∗^p < 0.01 in a Fisher’s exact test. See also Figures S2–S4 and Tables S2 and S3.

To gain insights into UPS functions, we examined how impairing individual UPS components affects yeast proteome abundance and turnover. We crossed the tFT library with an array of strains carrying knockout alleles of non-essential UPS components (Winzeler et al., 1999) or temperature-sensitive alleles of essential UPS factors (Li et al., 2011; Figure 2A; UPS array). The UPS array encompassed almost all known E2s, E3s, and DUBs, including substrate adaptors of E3s such as Rsp5 and SCF (Skp, Cullin, F-box-containing complex), several proteasomal subunits, and the autophagy factors Atg8 and Atg12 (Table S2). For large complexes, mutants of only one or a few core subunits were included in the array. We performed each cross in 4 technical replicates, grew the resulting haploids carrying tFT and UPS mutant alleles as ordered colony arrays, and measured their mCherry and sfGFP fluorescence. In total, we performed ∼2.5 million crosses, corresponding to more than 620,000 mutant-tFT pairs.

After correcting the data for crossing efficiency and plate and batch effects, we determined the effect of each of 132 UPS mutants on the abundance (sfGFP intensity) and stability (mCherry/sfGFP ratio) of each tFT-tagged protein (STAR Methods). Our screening procedure was robust, as shown by two sets of control screens. First, two tFT strains (*UBI4-tFT*, which should be stabilized in the absence of Ubr1, as detailed below, and the randomly chosen *YJR096W-tFT*) were screened in every batch a total of 24 times with reproducible results (Figure S2A). Second, a random set of 96 tFT strains was screened twice with good correlation between the replicates (Figures S2B and S2C; r > 0.7).

Overall, we detected significant changes in protein abundance or stability in ∼4.5% of mutant-tFT pairs (3.4% for abundance, 1.9% for stability; Figure 2B; Table S3). This frequency is similar to that of protein-protein interactions (Yu et al., 2008) or genetic interactions (Costanzo et al., 2016). Changes in protein abundance generally correlated with changes in stability, where stabilization was more frequently accompanied by an increase rather than a decrease in abundance, and vice versa (Figure 2B). Instances of anticorrelated changes in protein abundance and stability could reflect more complex regulation or be caused by the higher error associated with estimating changes in mCherry/sfGFP ratios. Globally, proteins that were stabilized in the screen were less stable in wild-type cells (Figure S2D), further arguing for the validity of our approach. On average, the abundance or stability of a given protein were affected in only ∼1–2 mutants (Figures 2C, S2E, and S2F). Notably, in total, only ∼56% of proteins were affected in at least one of the tested mutants (33% and 52% with changes in stability and abundance, respectively), which could be explained by condition-specific proteome turnover and redundancies in the UPS. Supporting this notion, mutants of 6 of 16 adaptors of the Rsp5 E3 had no effect on proteome turnover (Figure S2G), consistent with their high redundancy and conditional functions (Nikko and Pelham, 2009). Overall, the number of proteins affected by a given mutant varied greatly (Figures 2D and S2G–S2I). Cumulatively more proteins were affected by mutants of E3s compared with DUBs or E2 enzymes (Figures 2E and S2J), suggesting higher levels of redundancy between E2 enzymes or DUBs compared with E3s. It is also possible that some ubiquitination events are not accessible to DUBs and, thus, not affected in any DUB mutant.

Hereafter, we refer to mutant-tFT pairs where the stability or abundance of the tFT-tagged protein is affected significantly as turnover or abundance interactions, respectively. To understand the nature of these interactions, we compared them with various datasets: protein-protein interactions, collected from high-throughput and low-throughput experiments in the BioGRID (biological general repository for interaction datasets) (Oughtred et al., 2019); a dataset of genome-wide changes in mRNA expression and correlations of expression profiles determined for deletion mutants of ∼25% of all protein-coding genes, including UPS components (Kemmeren et al., 2014); and a genome-wide dataset of genetic interactions, measured by comparing the fitness of single and double mutants and corresponding correlations of genetic interaction profiles determined for ∼90% of all yeast genes (Costanzo et al., 2016). Turnover and abundance interactions were supported by protein-protein interactions between UPS factors and the affected proteins (Figures 2F and S2K). This is expected when substrates of selective protein degradation are affected in mutants of the corresponding targeting machinery. Indeed, we observed stabilization of well-defined substrates of various E3s in the screen (Figure S3A). Turnover and abundance interactions were also supported by changes in gene expression, and, interestingly, by genetic interactions and correlations of genetic interaction profiles (Figures 2F and S2K), which are an indication of functional similarity (Costanzo et al., 2016). This suggests that a fraction of interactions occurred between functionally related factors. Supporting this notion, we observed a significant degree of self-regulation in the UPS; the frequency of turnover interactions within the UPS was 3.2% compared with 1.9% for the whole proteome (Figures 2B, S3B, and S3C).

Next, we used turnover interactions to explore functions of various UPS components in more detail.

**Figure 3:**
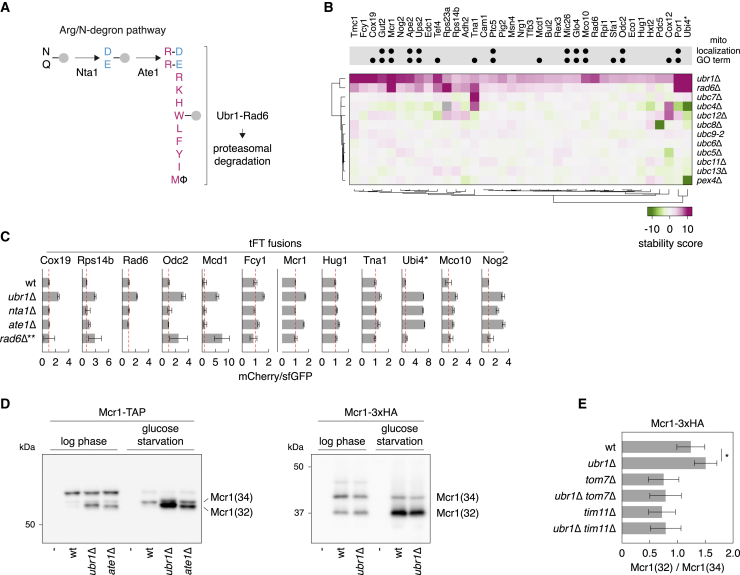
Ubr1-dependent protein turnover (A) Scheme of the Arg/N-degron pathway, which targets proteins with the indicated N-terminal residues for degradation. Φ, large hydrophobic residues (W, L, F, Y, I). (B) Heatmap of protein stability changes in the absence of Ubr1 (screens in Figure 2). Changes in mCherry/sfGFP ratios are color coded from green (decrease) to magenta (increase). Only proteins stabilized in the *ubr1*Δ mutant (1% FDR, stability score >4) are shown; their behavior in E2 mutants is included for comparison. Proteins localized to mitochondria based on GFP tagging (Huh et al., 2003) or mapped to the GO term mitochondrion are marked. ^∗^, Ubi4-tFT is not stabilized in the *ubr1*Δ mutant. Processing of the Ubi4-tFT fusion by DUBs releases free tFT with an N-terminal asparagine, which is the substrate of the Arg/N-degron pathway (B and C). (C) mCherry/sfGFP ratios of colonies expressing tFT fusions and lacking components of the Arg/N-degron pathway (mean ± SD, n = 4). Hereafter, red dashed lines mark mCherry/sfGFP ratios in the wild type (WT). ^∗∗^, protein stability measurements in the *rad6*Δ mutant are confounded by its fitness defect; this effect is partially corrected for in the screen (B). (D) Immunoblots of strains expressing Mcr1-TAP (left) or Mcr1-3xHA (right). Samples were collected from log-phase cultures or after 48 h of growth in low-glucose medium (glucose starvation). (E) Quantification of Mcr1(32) and Mcr1(34) relative abundance by immunoblotting of strains expressing Mcr1-3xHA (mean ± SD, n = 2 biological replicates each with 3 technical replicates). ^∗^p < 0.04 in a one-sided unpaired t test. See also Figure S4.

**Ltn1**. Ltn1 is an E3 that functions in ribosome-associated protein quality control (RQC). It is involved in targeting for proteasomal degradation stalled nascent polypeptides resulting from translation of mRNAs lacking a stop codon, which leads to translation of poly(A) tails into polylysine tracts, or from translation of mRNAs encoding a strong polybasic tract (Bengtson and Joazeiro, 2010; Brandman et al., 2012; Joazeiro, 2019). In these cases, electrostatic interactions between polybasic tracts and the ribosome exit tunnel are thought to cause ribosome stalling (Lu and Deutsch, 2008). Interestingly, Ltn1 appears to control the levels of Rqc1, another RQC factor. This control depends on a polybasic stretch located in the N-terminal portion of Rqc1, raising the possibility that Rqc1 is an Ltn1 substrate (Brandman et al., 2012). Indeed, we observed strong stabilization of Rqc1 in the absence of Ltn1 (Figure S4A). Surprisingly, of 62 yeast proteins that contain a strong polybasic stretch (Brandman et al., 2012), only Rqc1 and Nop12 were stabilized in *ltn1*Δ cells (Figure S4A). Therefore, polybasic stretches in endogenous proteins do not commonly lead to Ltn1-dependent degradation, at least under our experimental conditions, in agreement with a recent report (Barros et al., 2021).
**Ubc13-Mms2**. It is worth noting that a given UPS mutant, on average, stabilized and destabilized a similar number of proteins (Figure S2G), indicating that a significant fraction of turnover and abundance interactions is likely explained by indirect effects or adaptation of mutant strains to long-term loss of UPS factors. Nevertheless, gene set enrichment analysis suggested that, for ∼48% (63 of 132) of UPS mutants, the phenotypes were specific because the affected proteins were associated with defined signatures (Figure S4B). For instance, the Asi, Hrd1, and Tul1 E3s are involved in turnover of proteins in the endomembrane system (Foresti et al., 2014; Khmelinskii et al., 2014; Reggiori and Pelham, 2002; Ruggiano et al., 2014; Yang et al., 2018). Accordingly, proteins affected in the corresponding mutants were enriched in transmembrane domains and localized to the endoplasmic reticulum or vacuole. Interestingly, we observed a similar trend for Ubc13 (Figure S4B). Together with Mms2, Ubc13 forms a heteromeric E2 enzyme involved in replication of damaged DNA (Hofmann and Pickart, 1999). However, a second role of Ubc13-Mms2 in sorting of membrane proteins has been reported recently (Renz et al., 2020). Indeed, 13 of 25 proteins affected in *ubc13*Δ cells (an *mms2*Δ mutant was not included in the screen) had a transmembrane domain compared with 17% in the tFT library (Figure S4C), indicating that sorting of membrane proteins is a key function of Ubc13-Mms2 under unstressed conditions.
**Ubr1**. The E3 Ubr1 can target for proteasomal degradation proteins carrying N-degrons (Varshavsky, 2011). In the Arg/N-degron pathway, such N-degrons are typically exposed through endoproteolytic cleavage and, depending on the identity of the new N-terminal residue, recognized by Ubr1 directly or upon modification by the N-terminal amidase Nta1 and/or the N-terminal arginyl-transferase Ate1 (Varshavsky, 2011; Figure 3A). Two substrates of the Arg/N-degron pathways were among the proteins stabilized in the absence of Ubr1 or the cognate E2 enzyme Rad6: the cohesin subunit Mcd1, which is subject to proteolytic cleavage by the protease Esp1/separase, exposing a destabilizing arginine residue at the N terminus (Rao et al., 2001), and the N-tFT protein generated in the *UBI4-tFT* strain (Figure 3B). Ubi4 encodes a polyubiquitin precursor that is efficiently processed into free ubiquitin by DUBs (Özkaynak et al., 1984). In addition to free ubiquitin, processing of tFT-tagged Ubi4 results in a free tFT moiety with an N-terminal asparagine residue (N-tFT), which is then modified by Nta1 and Ate1 before Ubr1-mediated degradation. Accordingly, the mCherry/sfGFP ratio of the *UBI4-tFT* strain was increased upon deletion of *NTA1*, *ATE1*, or *UBR1* (Figure 3C). Interestingly, the same phenotype was observed in the autophagy mutants *atg8*Δ and *atg12*Δ (Figure S2A), although the reasons for this stabilization are unclear.

Multiple potential Ubr1 substrates were also stabilized in the *nta1*Δ or *ate1*Δ mutants (Figures 3B and 3C), suggesting that these proteins carry N-degrons exposed via proteolytic processing. We thus used immunoblotting to search for potential proteolytic fragments in strains expressing C-terminally TAP-tagged proteins. During logarithmic growth, we observed accumulation of an Mcr1 fragment in *ubr1*Δ cells. This phenotype was exacerbated upon glucose starvation (Figure 3D), a condition that is closer to cells in a colony (Cáp et al., 2012). Mcr1 is a mitochondrial NADH-cytochrome b5 reductase that exists in two isoforms: a full-length, 34-kDa protein inserted into the outer mitochondrial membrane (Mcr1(34)) and a shorter, 32-kDa isoform located in the intermembrane space (Mcr1(32)) (Hahne et al., 1994). This shorter isoform results from Mcr1 proteolysis by the mitochondrial inner membrane peptidase Imp1, which exposes a glutamate residue at the Mcr1(32) N terminus (Hahne et al., 1994), making it a potential Ate1 substrate (Figure 3A). Accordingly, Mcr1-tFT was stabilized in *ate1*Δ cells, and Mcr1(32) accumulated in the absence of Ate1 or upon proteasome inhibition (Figures 3C, 3D, and S4D).

We asked whether the bulky tFT and TAP tags might interfere with mitochondrial import of Mcr1 and explain these phenotypes. Mcr1(32) fused a small 3× hemagglutinin (HA) tag also accumulated in *ubr1*Δ cells, as evidenced by a higher Mcr1(32)/Mcr1(34) ratio in the *ubr1*Δ mutant compared with the wild-type, although to a substantially reduced extent relative to the TAP-tagged variant (Figures 3D and 3E). Inhibiting Mcr1-3×HA import into mitochondria (thus preventing its processing by Imp1) by deletion of the Tom7 and Tim11 subunits of the translocases of the outer and inner membrane complexes, respectively, prevented Mcr1(32) accumulation in the absence of Ubr1 (Figure 3E). These results suggest that incomplete mitochondrial import of Mcr1 (e.g., caused by a bulky tag) leads to release of N-terminally processed Mcr1(32) into the cytosol, where Ubr1 and Ate1 localize (Huh et al., 2003; Varshavsky, 2011), followed by its Ate1/Ubr1-dependent degradation. Although Mcr1(32) accumulation can be modulated by the tag, we cannot formally exclude that altered Imp1 activity in Arg/N-degron mutants contributes to this phenotype. Nevertheless, considering the preference of Imp1 for aspartate and glutamate residues at the P’1 position (Luo et al., 2006), which becomes the new N terminus upon proteolysis, it seems likely that Ubr1 plays a more general role in quality control of mitochondrial protein import (Bragoszewski et al., 2015). An analogous pathway appears to operate in human cells, as exemplified by regulation of PINK1 (PTEN-induced kinase 1) in mitophagy. Whereas PINK1 accumulates in the outer membrane of damaged mitochondria and promotes mitophagy, under normal conditions, PINK1 is processed by the mitochondrial inner membrane rhomboid protease PARL (presenilin associated rhomboid like) and released into the cytosol, where it is targeted for degradation by the Arg/N-degron pathway (Greene et al., 2012; Harper et al., 2018; Jin et al., 2010; Meissner et al., 2011; Yamano and Youle, 2013). It will be interesting to examine whether the Arg/N-degron pathway plays a broader role in shaping the human mitochondrial proteome. This analysis demonstrates how protein turnover and abundance interactions can be used to identify new functions of selective protein degradation machinery.

### Correlations of proteome turnover profiles

**Figure 4:**
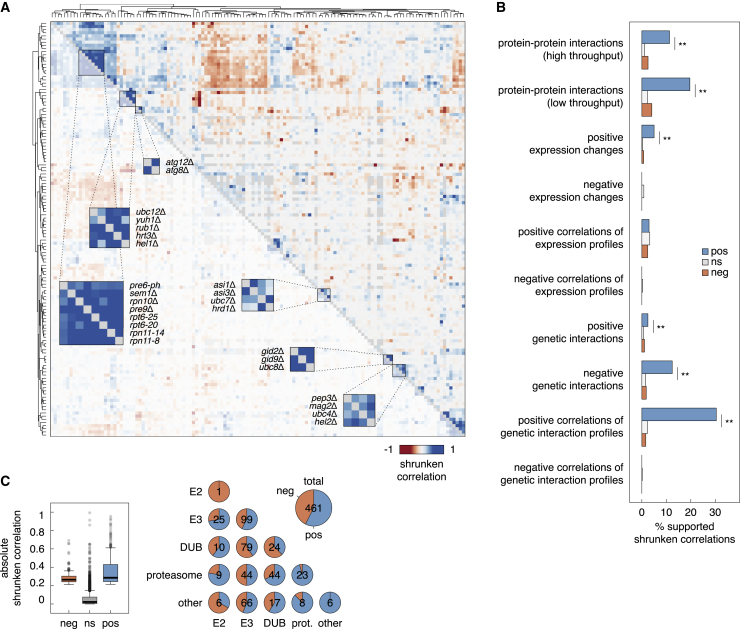
Correlations of proteome turnover profiles (A) Heatmap of correlations of proteome turnover profiles for all tested mutants in the UPS array (screens in Figure 2). For each pair of mutants, a shrunken correlation was calculated based on the set of proteins with altered stability (1% FDR, absolute stability score >4) in at least one of the mutants. Select clusters of correlating mutants are highlighted. (B) Overlap between shrunken correlations of proteome turnover profiles and external datasets. Correlations of proteome turnover profiles from (A) were grouped according to significance (1% FDR) and sign (pos, positive; ns, not significant; neg, negative). ^∗∗^p < 0.01 in a Fisher’s exact test. (C) Magnitude of shrunken correlations of proteome turnover profiles grouped according to significance and sign as in (B) (left) and number of significant correlations between UPS mutants grouped by function (Table S2) (right). See also Figures S5 and S6 and Table S4.

Next we explored our dataset to gain insights into functional relationships between UPS components. We calculated correlations of proteome turnover profiles between pairs of UPS mutants, adjusted for the number of affected proteins (Figures 4A and S5A–S5C; Table S4; STAR Methods). Up to 30% of positive correlations of proteome turnover profiles were supported by protein-protein interactions (Oughtred et al., 2019) between UPS components, genetic interactions, and correlations of genetic interaction profiles (Costanzo et al., 2016; Figure 4B), indicating that UPS components with similar proteome turnover profiles are likely to interact physically or to be involved in the same process. Correlations of proteome turnover profiles provided stronger evidence of functional similarity compared with correlations of genetic interactions profiles because proteome turnover is likely a more informative phenotype to dissect functions of UPS components compared with fitness-based genetic interactions (Costanzo et al., 2016). This is exemplified by correlations between mutants of the Asi E3 and the Ubc7 E2 enzyme, which participate in protein quality control at the inner nuclear membrane (Foresti et al., 2014; Khmelinskii et al., 2014; Figure S5D). We also expected to observe negative correlations of proteome turnover profiles because of the existence of opposing activities in the UPS, for instance, E3s and DUBs. Indeed, 43% of significant correlations in our dataset were negative, including 47 of 79 significant correlations between E3s and DUBs and 14 of 44 significant correlations between DUBs and the proteasome (Figure 4C; Table S4). Factors with anticorrelated phenotypes are less likely to be in one complex because negative correlations of proteome turnover profiles were not supported by protein-protein interactions (Figure 4B). These anticorrelations appeared to not be captured by correlations of fitness-based genetic interaction profiles, possibly because of the less specific phenotype or because of reduced sensitivity of the fitness assay toward positive genetic interactions (Baryshnikova et al., 2010a).

**Figure 5:**
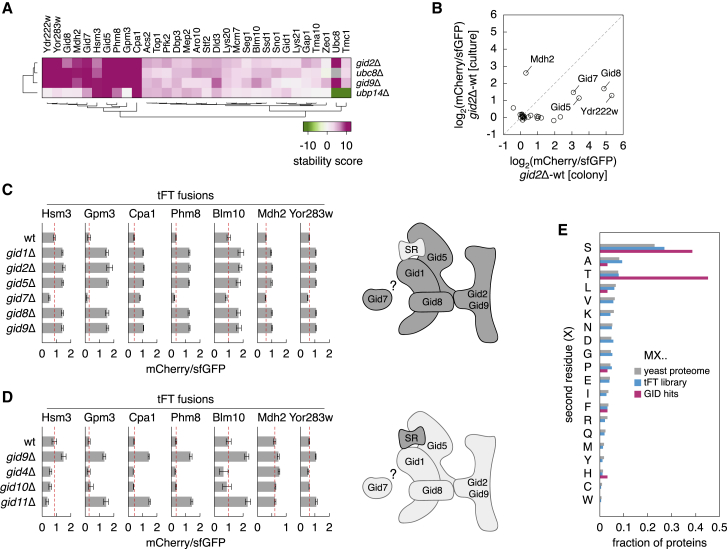
Protein turnover by the GID complex (A) Heatmap of protein stability changes in the absence of GID components (screens in Figure 2). Only proteins stabilized in at least one of the mutants (*gid2*Δ, *gid9*Δ, and *ubc8*Δ; 1% FDR, stability score >4) are shown; their behavior in the *ubp14*Δ mutant is included for comparison. (B) Differences in mCherry/sfGFP ratios between *gid2*Δ and WT cells for tFT-tagged proteins from (A). Flow cytometry measurements of log-phase cultures and plate reader measurements of colonies (mean, n = 4). Proteins stabilized in the *gid2*Δ background in cultures are indicated. (C and D) mCherry/sfGFP ratios of colonies expressing tFT fusions and lacking GID components (mean ± SD, n = 4) and cartoon of the GID complex; SR, substrate receptor (right; adapted from Qiao et al., 2020). (E) Frequency of residues at the second position in potential GID substrates from (A) (GID hits). See also Figure S7 and Table S5.

Based on this analysis, we conclude that correlations of proteome turnover profiles are a measure of functional similarity that could be used to identify complexes or pathways in the UPS. Supporting this notion, mutants of proteasomal components (Pre6, Sem1, Rpn10, Pre9, Rpt6, and Rpn11) exhibited similar proteome turnover profiles and clustered together (Figure 4A). The same behavior was observed for the two mutants in autophagy genes, *atg8*Δ and *atg12*Δ; for mutants lacking subunits of the GID complex, Gid2 and Gid9; or for mutants in Asi and Hrd1 E3s, which have overlapping roles in protein abundance and quality control at the inner nuclear membrane and the endoplasmic reticulum (Foresti et al., 2014; Khmelinskii et al., 2014; Ruggiano et al., 2014; Figure 4A). Most correlations between DUBs and the proteasome were positive because of two DUBs, Rpn11 and Ubp6 (Figure 4C; Table S4), which are involved in recycling ubiquitin from ubiquitin-protein conjugates at the proteasome (Hanna et al., 2006; Leggett et al., 2002; Verma et al., 2002; Yao and Cohen, 2002). Correlations of proteome turnover profiles also revealed new functional relationships, for example:

**Hel2-Mag2**. Hel2 was initially identified as an E3 that targets excess histones for degradation (Singh et al., 2012). In addition, Hel2 and its human homolog ZNF598 are involved in RQC, where Hel2-mediated ubiquitination of the 40S small ribosomal subunit within a collided di-ribosome is required to trigger RQC (Ikeuchi et al., 2019; Juszkiewicz and Hegde, 2017; Juszkiewicz et al., 2018; Matsuo et al., 2017; Sitron et al., 2017; Sundaramoorthy et al., 2017; Winz et al., 2019). Accordingly, turnover of histones and components of the 40S ribosome was affected in the *hel2*Δ mutant (Figure S6A). This phenotype correlated with phenotypes of mutants lacking Ubc4, the E2 enzyme that interacts with Hel2 (Singh et al., 2012), and Mag2 (Figures 4A and S6A). Mag2 is a poorly characterized RING E3 that, together with Hel2 and the E3 Rsp5, was recently implicated in degradation of non-functional 18S rRNA in a process that also involves ubiquitination of the 40S small ribosomal subunit (Sugiyama et al., 2019). Lack of Mag2 resulted in stabilization of several ribosome components, specifically of the 40S ribosome (Figure S6B). However, this phenotype was anticorrelated with that of *rsp5* mutants (Figure S6A), suggesting that, besides its role in degradation of non-functional 18S rRNA, Mag2 could also be involved in RQC.
**Hel1-SCF**^**Hrt3**^. Another set of correlations involved components of the cullin-RING E3 SCF (the cullin 1 Cdc53 and the F-box substrate adaptor Hrt3), the neddylation machinery (the ubiquitin-like modifier Rub1/Nedd8, the cognate E2 Ubc12, and the DUB Yuh1), the RING-IBR-RING (RBR) E3 of the Ariadne family Hel1, the RING E3 Nam7, and the DUB Ubp10 (Figures 4A and S6C–S6F). Although neddylation of cullins is required for robust activity of cullin-RING E3s, the neddylation machinery is not essential in yeast (Lammer et al., 1998; Liakopoulos et al., 1998; Willems et al., 2004). Accordingly, few proteins were affected in the *rub1*Δ and *yuh1*Δ strains compared with the *cdc53-1* mutant (Figures S2G and S2H). The phenotypes of the *rub1*Δ and *yuh1*Δ mutants were largely restricted to stabilization of the translation elongation factor 2 (eEF2), which, in yeast, is encoded by two paralogs, *EFT1* and *EFT2.* In fact, the whole set of correlations was mostly driven by changes in the stability of Eft1 and Eft2 (Figures S6C–S6F). Interestingly, Eft1-tFT was less affected in logarithmically growing cultures compared with colonies (Figure S6G), indicating conditional regulation of eEF2. Using Eft1-tFT stability in colonies as a readout, we further assessed the relationships between these correlating UPS components. Eft1 destabilization in the *ubp10*Δ and *nam7*Δ mutants depended on Hel1 and Hrt3, placing these factors in one pathway (Figure S6H). Deletion of *HRT3* or *HEL1* stabilized Eft1-tFT to a similar extent, and no further stabilization was detected in the *hrt3*Δ *hel1*Δ double mutant, suggesting that Hel1 and SCF^Hrt3^ cooperate in substrate ubiquitination. In human cells and in *C. elegans*, cullin-RING E3s associate and work together with an Ariadne family RBR, ARIH1/HHARI (human homolog of *Drosophila* Ariadne-1) (Dove et al., 2017; Scott et al., 2016). Our results point toward similar cooperation between Hel1 and SCF^Hrt3^ in yeast, possibly in conditional control of translation.
**GID complex**. The GID E3 is involved in degradation of gluconeogenic enzymes when switching from gluconeogenesis to glycolysis (Regelmann et al., 2003; Santt et al., 2008). Only one of its substrates, the malate dehydrogenase Mdh2 (Hung et al., 2004; Santt et al., 2008), was in the tFT library. Mdh2 was stabilized in the absence of Gid2 or Gid9, the two RING subunits, and in cells lacking Ubc8, the E2 that works with GID (Figure 5A). Ubc8 appears to function exclusively with the GID E3 under the screen conditions because the phenotypes of *ubc8*Δ, *gid2*Δ and *gid9*Δ mutants were almost identical (Figures 4A and S7A). The DUB Ubp14 has been implicated previously in degradation of GID substrates (Eisele et al., 2006; Regelmann et al., 2003). However, not all potential GID substrates were affected in the *ubp14*Δ mutant (Figure 5A). The *ubp14*Δ and *gid2*Δ phenotypes were not additive (Figure S7B), indicating that Ubp14 promotes protein degradation with the GID E3 but in a substrate-specific manner.

In total, 31 proteins were stabilized in the *gid2*Δ, *gid9*Δ, or *ubc8*Δ mutants (Figure 5A). These included several GID subunits and three potential GID substrates (Aro10, Tma10, and Stf2) identified in a recent proteomic study (Karayel et al., 2020). For most tFT fusions, their stabilization was detectable in colonies, which consist of cells in different metabolic states (Cáp et al., 2012), but not in logarithmically growing cultures with glucose as a carbon source (Figure 5B). This is consistent with the idea that GID regulates protein turnover in metabolic transitions or in response to stress (Melnykov et al., 2019) and highlights how colony-based proteome profiling can reveal conditional phenotypes. Metabolic heterogeneity of colonies also complicates direct comparison with pulse SILAC proteomic profiling of the UPS (Christiano et al., 2020). GID-dependent turnover of Mdh2, Ydr222w, and several GID subunits in log-phase cultures (Figure 5B) suggests that the GID E3 is also active under steady-state conditions, consistent with previous observations (Menssen et al., 2018).

Next we examined the role of other GID components in turnover of potential GID substrates. All tested tFT fusions were stabilized in mutants lacking any single GID subunit except Gid7 (Figure 5C). In contrast, loss of Ipf1 or Moh1, which co-purify with the GID complex (Ho et al., 2002; Subbotin and Chait, 2014), did not affect any tFT fusions (Figures S7C and S7D). Lack of protein stabilization in the *gid7*Δ mutant is consistent with the reported structure of a functional GID complex, which does not include Gid7 (Qiao et al., 2020). Nevertheless, it is possible that a variant GID complex containing Gid7 exists, considering that Gid7 is involved in Gid4-depedent turnover of Fbp1 (Regelmann et al., 2003), and correlations of genetic interaction profiles indicate that *GID7* is functionally related to core GID subunits (Costanzo et al., 2016; Figure S7E). Moreover, turnover of Gid7-tFT was affected in various *gid* mutants, similar to other GID subunits (Figure S7D).

GID recognizes substrates via N-degrons using the interchangeable receptor subunits Gid4 and Gid10 (Chen et al., 2017; Melnykov et al., 2019; Qiao et al., 2020). Although Gid4 recognizes substrates such as Mdh2 via N-degrons with an N-terminal proline (Chen et al., 2017; Hämmerle et al., 1998), the partially overlapping specificity of Gid10 is less understood (Melnykov et al., 2019). In the case of Mdh2, proline is exposed at the N terminus after co-translational removal of the initiator methionine by methionine aminopeptidases (MetAPs), whose substrates comprise N termini with small residues (A, S, T, V, C, G, P) after the initiator methionine (Moerschell et al., 1990; Varland et al., 2015). As expected, Mdh2 was stabilized in the absence of Gid4 in the tFT assay. However, deletion of *GID4* or *GID10* did not affect any tested potential GID substrates (Figure 5D).

**Figure 6:**
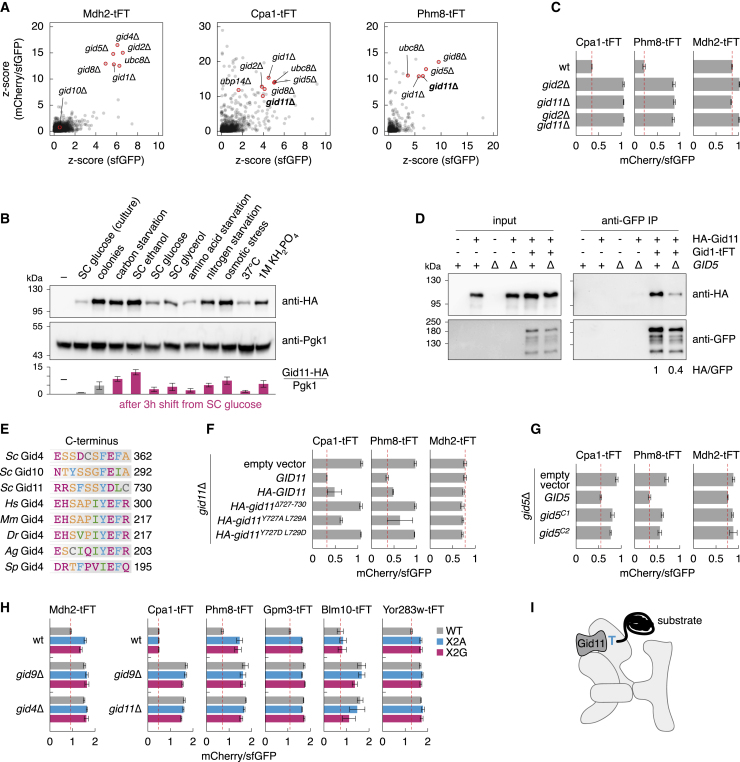
Gid11-dependent turnover of proteins with an N-terminal threonine (A) Genome-wide screens for factors involved in turnover of Cpa1 and Phm8. Only gene deletions with positive *Z* scores are shown. (B) Immunoblot of strains expressing chromosomally tagged Gid11-HA (top) and quantification of relative Gid11-HA expression levels (bottom, mean ± SD, n ≥ 3). Samples were collected from log-phase cultures in synthetic complete medium with glucose as a carbon source (SC glucose) from colonies or after a 3-h shift from SC glucose into the indicated environment (red). (C) mCherry/sfGFP ratios of colonies expressing tFT fusions and lacking *GID2* and/or *GID11* (mean ± SD, n = 4). (D) Co-immunoprecipitation of overexpressed HA-Gid11 and chromosomally tagged Gid1-tFT. The relative amount of co-immunoprecipitated HA-Gid11, normalized to precipitated Gid1-tFT, was reduced to 0.43 ± 0.15 (mean ± SD, n = 3) in *gid5*Δ cells compared with the WT. (E) C termini of GID receptors from different organisms. Sc, *S. cerevisiae*; Hs, *Homo sapiens*; Mm, *Mus musculus*; Dr, *Danio rerio*; Ag, *Anopheles gambiae*; Sp, *Schizosaccharomyces pombe*. (F and G) mCherry/sfGFP ratios of colonies expressing tFT fusions (mean ± SD, n = 3 [F] or n = 4 [G]). Dashed lines mark mCherry/sfGFP ratios in *gid11*Δ or *gid5*Δ mutants complemented with WT *GID11* or *GID5*, respectively. (H) mCherry/sfGFP ratios of colonies expressing tFT-tagged proteins, either WT or with the second residue X mutated to alanine (X2A) or glycine (X2G) (mean ± SD, n ≥ 3). (I) Model of Gid11 as a receptor for substrates with an N-terminal threonine, exposed after removal of the initiator methionine by MetAPs. Gid11-dependent protein turnover requires all core GID subunits but not Gid7. See also Figure S7 and Tables S6 and S7.

Interestingly, 26 of the 31 potential GID substrates have a serine or a threonine after the initiator methionine (Figure 5E; Table S5). We thus asked whether an unknown GID receptor was involved in their recognition. To identify such a receptor, we performed genetic screens with two potential substrates, the carbamoyl phosphate synthetase Cpa1 (N terminus MSSAA) and the nucleotidase Phm8 (N terminus MTIAK). We crossed *CPA1-tFT* and *PHM8-tFT* strains with a genome-wide knockout library (Winzeler et al., 1999) and measured the mCherry and sfGFP fluorescence of the resulting colonies to identify mutants that increased the abundance and stability of each fusion. In the control screen with Mdh2-tFT, we identified mutants of *UBC8* and of all known GID subunits except *GID9* (absent from the knockout library), *GID7* and *GID10* (Figure 6A). Among the top hits for Cpa1-tFT and Phm8-tFT were mutants of several GID subunits and a strain lacking *YLR149C*, a gene of unknown function that we named *GID11* (Figure 6A; Table S6). Gid11 is a WD40/YVTN repeat-like domain protein conserved across yeasts (Figure S7F; Table S7). Gid11 expression was higher in colonies compared with log-phase cultures with glucose as a carbon source and was upregulated by using ethanol as a carbon source or by various stresses, including carbon starvation, nitrogen starvation, and hyperosmotic stress (Figure 6B). This is consistent with the conditional nature of GID phenotypes (Figure 5B).

Although the genetic interaction profile of *GID11* correlated poorly with other *GID* genes (Figure S7E), deletion of *GID11* stabilized Cpa1-tFT and Phm8-tFT to the same extent as deletion of *GID2*, and no further stabilization was seen in the *gid2*Δ *gid11*Δ double mutant (Figure 6C). This suggests that Gid11 functions with the GID E3. We thus tested whether Gid11 interacts with GID subunits in co-immunoprecipitation experiments. Using strains expressing tFT-tagged GID subunits and HA-Gid11 overexpression during logarithmic growth, we detected an interaction between Gid11 and Gid1 (Figures 6D and S7G). This interaction was dependent on Gid5 (Figure 6D), which also recruits the Gid4 and Gid10 receptors to the GID complex (Melnykov et al., 2019; Menssen et al., 2012; Qiao et al., 2020).

Gid4 and Gid10 interact with Gid5 via C-terminal anchors that end with a conserved ΦEΦX motif (where Φ denotes a hydrophobic residue) (Qiao et al., 2020; Figure 6E). Gid11 has a similar C-terminal YDLC motif that only differs in the acidic residue D instead of E (Figures 6E and S7F), raising the possibility of an analogous mode of interaction between Gid11 and Gid5. To test this, we first examined how mutations in the Gid11 C terminus affect its function. Although plasmid-borne expression of HA-Gid11 could complement a *gid11*Δ mutant and restore turnover of Cpa1-tFT and Phm8-tFT, deleting the last 4 residues in Gid11 (Gid11^Δ727–730^) or replacing the C-terminal hydrophobic residues with charged ones (Gid11^Y727D L729D^) resulted in non-functional Gid11 variants (Figure 6F). Second, the Gid4 C-terminal anchor interacts with the C-terminal domain of Gid5. Gid5 variants with mutations at this interaction interface, such as Gid5^C1^ (W606A, H610A, and Y613A) and Gid5^C2^ (W606A, Y613A, and Q649A), show impaired Gid4-dependent ubiquitination and turnover of gluconeogenic enzymes (Qiao et al., 2020). The Gid5^C1^ and Gid5^C2^ mutants were also defective in Gid11-dependent turnover of Cpa1 and Phm8 (Figure 6G). Finally, fusing the last 20 residues of Gid11 to luciferase (HA-Luc-Gid11^C20^) was sufficient to recruit it to the GID complex, as evidenced by co-immunoprecipitation of HA-Luc-Gid11^C20^ with Gid1 (Figure S7H). These results argue that Gid11 is recruited to the GID complex by a C-terminal anchor that interacts with Gid5, similar to the Gid4 and Gid10 receptors.

Next we determined the spectrum of Gid11 substrates. Using the tFT assay, we tested whether turnover of potential GID substrates (Figure 5A) depends on *GID11*. Remarkably, 12 of the 14 proteins with an N-terminal threonine were stabilized in the absence of Gid11 to the same extent as in a *gid9*Δ mutant (Figures 5D and S7I). The only exceptions were two GID subunits, Gid5 and Gid8. In contrast, of the 16 proteins with residues other than threonine after the initiator methionine, only Cpa1 showed Gid11-dependent turnover (Figure S7I). Therefore, we tested how Gid11-dependent protein turnover depends on the substrate N terminus. As controls, we mutated the N-terminal proline of Mdh2 to alanine (Mdh2^P2A^) or glycine residues (Mdh2^P2G^), which do not prevent removal of the initiator methionine by MetAPs (Moerschell et al., 1990; Varland et al., 2015). Both mutations preclude Gid4 binding to the Mdh2 N terminus and largely abolished Gid4-dependent turnover of Mdh2-tFT (Chen et al., 2017; Hämmerle et al., 1998; Figure 6H). The same mutations in Cpa1 and Blm10 had no obvious effect on their turnover. It is possible that Cpa1 and Blm10 (N terminus MTANN) stabilization in *gid* mutants is indirect or that their recognition is more complex. Nevertheless, Phm8, Gpm3, and Yor283w variants with the N-terminal threonine mutated to alanine or glycine were stable compared with wild-type proteins and were not stabilized further by deletion of *GID9* or *GID11* (Figure 6H), indicating that their N termini carry degrons recognized by the GID complex. In addition, we replaced the N terminus of Phm8 with N termini of other GID substrates. Only sequences with an N-terminal threonine allowed Gid11-dependent turnover of Phm8 (Figure S7J). These results suggest that Gid11 is a substrate receptor of the GID complex that recognizes degrons with an N-terminal threonine (Figure 6I). The precise N-degron motif bound by Gid11 and the role of MetAPs and N-terminal acetyltransferases, which should modify such N termini, in Gid11-dependent protein turnover remain to be determined.

The vertebrate GID/CTLH (C-terminal to LisH) complex is linked to a variety of processes, including cilium function, the cell cycle, and metabolism (Boldt et al., 2016; Lampert et al., 2018; Leal-Esteban et al., 2018; Liu and Pfirrmann, 2019; Liu et al., 2020; Pfirrmann et al., 2015; Texier et al., 2014). Yeast and human Gid4 appear to recognize similar N-degrons, although no substrates of human Gid4 are known (Chen et al., 2017; Dong et al., 2018, 2020), and N termini of known substrates do not fit the Gid4 consensus (Lampert et al., 2018). It will therefore be important to understand how the expanded specificity of the GID complex described here translates outside of yeast.

## Discussion

As the key system of selective protein degradation, the UPS is connected to all cellular processes by destroying unnecessary or abnormal proteins at the right time and place (Hershko and Ciechanover, 1998; Kleiger and Mayor, 2014; Zheng and Shabek, 2017). This work provides a rich dataset to explore different functions of this system from the perspective of a protein of interest or with a UPS component as a starting point.

The proteomic approach applied here, although arguably more laborious compared with mass spectrometry-based proteomics (Christiano et al., 2020), has several advantages. First, because protein abundance and stability were measured in yeast colonies, which consist of metabolically heterogeneous populations (Cáp et al., 2012), our proteome profiling is sampling multiple environmental conditions in one experiment. This is evidenced by various conditional phenotypes identified in the screen. Second, the strains generated for proteome profiling could be used in other downstream analyses, including studies of noise in protein degradation or analysis of protein localization changes upon perturbation of selective protein degradation. Although proteome profiling is ideally suited to investigate mechanisms of selective protein degradation, this approach could be extended to other cellular processes as an unbiased phenotypic assay to characterize gene functions. Ultimately, integrating different phenotypic profiles, including genetic interactions and changes in transcriptome, proteome, and intracellular organization, should refine functional predictions and help dissect complex cellular processes.

### Limitations of study

Several protein classes known to be affected by C-terminal tagging, such as tail-anchored or glycosylphosphatidylinositol-anchored proteins, are excluded from the tFT library (Khmelinskii et al., 2014). Tagging could still impair turnover of some proteins (e.g., by blocking C-terminal degrons; Koren et al., 2018; Lin et al., 2018) or promote turnover of others because of the large size of the tFT tag, as seen with Ubr1-dependent Mcr1 turnover. In addition, potential substrates identified for various UPS components should be validated independently to rule out indirect effects or adaption of UPS mutants. Finally, the number of identified potential substrates is likely limited by redundancies within the UPS.

## STAR★Methods

### Key resources table

**Table:** 

REAGENT or RESOURCE	SOURCE	IDENTIFIER
**Antibodies**		

Mouse monoclonal anti-HA (clone 12CA5)	In-house	N/A
Mouse monoclonal anti-GFP (clones 7.1 and 13.1)	Sigma-Aldrich	Cat#11814460001; RRID: AB_390913
Rabbit Peroxidase anti-Peroxidase	Sigma-Aldrich	Cat#P1291; RRID: AB_1079562
Mouse monoclonal anti-Pgk1 (clone 22C5D8)	Thermo Fisher Scientific	Cat#459250; RRID: AB_2532235
Rabbit Peroxidase anti-Peroxidase	Dako	Cat#Z0113
Goat polyclonal anti-Mouse IgG, HRP-conjugated	Thermo Fisher Scientific	Cat#G-21040; RRID: AB_2536527
Goat polyclonal anti-Mouse IgG, HRP-conjugated	Dianova GmbH	Cat#115-035-003
Rabbit polyclonal anti-GFP	Abcam	Cat#ab290; RRID: AB_303395
Dynabeads M-280 Sheep Anti-Rabbit IgG	Thermo Fisher Scientific	Cat#11203D; RRID: AB_2783009

**Chemicals, peptides, and recombinant proteins**		

MG132	Enzo Life Sciences	Cat#BML-PI102-0025
Complete EDTA-free protease inhibitor cocktail	Sigma-Aldrich	Cat#4693159001

**Critical commercial assays**		

Pierce ECL Plus Western Blotting Substrate	Thermo Fisher Scientific	Cat#32132

**Deposited data**		

R vignette reproducing the analysis of the screens profiling the ubiquitin-proteasome system and containing the screen dataset	This study	https://heidata.uni-heidelberg.de/citation?persistentId=doi:10.11588/data/Q3TSLH
Unprocessed immunoblot images	This study	https://data.mendeley.com/datasets/rv8f9bp5b2

**Experimental models: organisms/strains**		

*Saccharomyces cerevisiae* strain BY4741: S288c MATa his3Δ1 leu2Δ0 met15Δ0 ura3Δ0	Brachmann et al., 1998	N/A
*Saccharomyces cerevisiae* strain Y8205: S288c MATalpha his3Δ1 leu2Δ0 met15Δ0 ura3Δ0 can1Δ::STE2pr-spHIS5 lyp1Δ::STE3pr-LEU2	Tong and Boone, 2007	N/A
*Saccharomyces cerevisiae* strain Y7092: S288c MATalpha his3Δ1 leu2Δ0 met15Δ0 ura3Δ0 can1Δ::STE2pr-SpHIS5 lyp1Δ	Tong and Boone, 2007	N/A
*Saccharomyces cerevisiae* strain BY4743: S288c MATa/alpha his3Δ1/his3Δ1 leu2Δ0/leu2Δ0 MET15/met15Δ0 LYS2/lys2Δ0 ura3Δ0/ura3Δ0	Brachmann et al., 1998	N/A
YMaM330: Y8205 leu2Δ::GAL1pr-I-SCEI-natNT2	Khmelinskii et al., 2014	N/A
tFT library: YMaM330 ORF-mCherry-SceIsite-SpCYC1term-ScURA3-SceIsite-mCherryΔN-sfGFP	Khmelinskii et al., 2014	N/A
UPS array: BY4741 orf::kanMX	This study	N/A
UPS array control_1: BY4741 his3Δ::kanMX	This study	N/A
UPS array control_2: BY4741 ura3Δ::kanMX	This study	N/A
AK1230: BY4741 ubr1Δ::kanMX6	This study	N/A
AK1231: BY4741 nta1Δ::kanMX6	This study	N/A
AK1232: BY4741 ate1Δ::kanMX6	This study	N/A
AK1233: BY4741 rad6Δ::kanMX6	This study	N/A
YIK138: BY4741 MCR1-TAP-HisMX	Ghaemmaghami et al., 2003	N/A
YIK101: BY4741 MCR1-TAP-HisMX ubr1Δ::natNT2	This study	N/A
YIK174: BY4741 MCR1-TAP-HisMX ate1Δ::natNT2	This study	N/A
YLZY0093: BY4741 MCR1-TAP-HisMX pdr5Δ::natNT2	This study	N/A
YIK179: BY4741 MCR1-3HA-kanMX	This study	N/A
YIK182: BY4741 MCR1-3HA-kanMX ubr1Δ::natNT2	This study	N/A
YIK189: BY4741 MCR1-3HA-kanMX tom7Δ::hphNT1	This study	N/A
YIK190: BY4741 MCR1-3HA-kanMX ubr1Δ::natNT2 tom7Δ::hphNT1	This study	N/A
YIK191: BY4741 MCR1-3HA-kanMX tim11Δ::hphNT1	This study	N/A
YIK192: BY4741 MCR1-3HA-kanMX ubr1Δ::natNT2 tim11Δ::hphNT1	This study	N/A
YBB228: YMaM330 EFT1-mCherry-sfGFP	This study	N/A
AK1293: YMaM330 EFT1-mCherry-sfGFP hel1Δ::kanMX6	This study	N/A
AK1312: YMaM330 EFT1-mCherry-sfGFP yuh1Δ::kanMX6	This study	N/A
AK1294: YMaM330 EFT1-mCherry-sfGFP ubc12Δ::kanMX6	This study	N/A
AK1295: YMaM330 EFT1-mCherry-sfGFP rub1Δ::kanMX6	This study	N/A
AK1296: YMaM330 EFT1-mCherry-sfGFP hrt3Δ::kanMX6	This study	N/A
AK1311: YMaM330 EFT1-mCherry-sfGFP ubp10Δ::kanMX6	This study	N/A
AK1297: YMaM330 EFT1-mCherry-sfGFP nam7Δ::kanMX6	This study	N/A
AK1326: BY4741 hel1Δ::hphNT1	This study	N/A
AK1322: BY4741 yuh1Δ::hphNT1	This study	N/A
AK1327: BY4741 ubc12Δ::hphNT1	This study	N/A
AK1328: BY4741 rub1Δ::hphNT1	This study	N/A
AK1329: BY4741 hrt3Δ::hphNT1	This study	N/A
AK1321: BY4741 ubp10Δ::hphNT1	This study	N/A
AK1330: BY4741 nam7Δ::hphNT1	This study	N/A
YMaM1074: YMaM330 HSM3-mCherry-sfGFP	This study	N/A
AK1248: YMaM330 HSM3-mCherry-sfGFP gid1Δ::kanMX6	This study	N/A
YMaM1110: YMaM330 HSM3-mCherry-sfGFP gid2Δ::kanMX6	This study	N/A
AK1249: YMaM330 HSM3-mCherry-sfGFP gid5Δ::kanMX6	This study	N/A
AK1250: YMaM330 HSM3-mCherry-sfGFP gid7Δ::kanMX6	This study	N/A
AK1251: YMaM330 HSM3-mCherry-sfGFP gid8Δ::kanMX6	This study	N/A
AK1252: YMaM330 HSM3-mCherry-sfGFP gid9Δ::kanMX6	This study	N/A
YMaM1079: YMaM330 GPM3-mCherry-sfGFP	This study	N/A
AK1254: YMaM330 GPM3-mCherry-sfGFP gid1Δ::kanMX6	This study	N/A
YMaM1115: YMaM330 GPM3-mCherry-sfGFP gid2Δ::kanMX6	This study	N/A
AK1255: YMaM330 GPM3-mCherry-sfGFP gid5Δ::kanMX6	This study	N/A
AK1256: YMaM330 GPM3-mCherry-sfGFP gid7Δ::kanMX6	This study	N/A
AK1257: YMaM330 GPM3-mCherry-sfGFP gid8Δ::kanMX6	This study	N/A
AK1258: YMaM330 GPM3-mCherry-sfGFP gid9Δ::kanMX6	This study	N/A
YMaM1080: YMaM330 CPA1-mCherry-sfGFP	This study	N/A
AK1260: YMaM330 CPA1-mCherry-sfGFP gid1Δ::kanMX6	This study	N/A
YMaM1116: YMaM330 CPA1-mCherry-sfGFP gid2Δ::kanMX6	This study	N/A
AK1261: YMaM330 CPA1-mCherry-sfGFP gid5Δ::kanMX6	This study	N/A
AK1262: YMaM330 CPA1-mCherry-sfGFP gid7Δ::kanMX6	This study	N/A
AK1263: YMaM330 CPA1-mCherry-sfGFP gid8Δ::kanMX6	This study	N/A
AK1264: YMaM330 CPA1-mCherry-sfGFP gid9Δ::kanMX6	This study	N/A
YMaM1082: YMaM330 PHM8-mCherry-sfGFP	This study	N/A
AK1265: YMaM330 PHM8-mCherry-sfGFP gid1Δ::kanMX6	This study	N/A
YMaM1118: YMaM330 PHM8-mCherry-sfGFP gid2Δ::kanMX6	This study	N/A
AK1266: YMaM330 PHM8-mCherry-sfGFP gid5Δ::kanMX6	This study	N/A
AK1267: YMaM330 PHM8-mCherry-sfGFP gid7Δ::kanMX6	This study	N/A
AK1268: YMaM330 PHM8-mCherry-sfGFP gid8Δ::kanMX6	This study	N/A
AK1269: YMaM330 PHM8-mCherry-sfGFP gid9Δ::kanMX6	This study	N/A
YMaM1090: YMaM330 BLM10-mCherry-sfGFP	This study	N/A
AK1270: YMaM330 BLM10-mCherry-sfGFP gid1Δ::kanMX6	This study	N/A
YMaM1126: YMaM330 BLM10-mCherry-sfGFP gid2Δ::kanMX6	This study	N/A
AK1271: YMaM330 BLM10-mCherry-sfGFP gid5Δ::kanMX6	This study	N/A
AK1272: YMaM330 BLM10-mCherry-sfGFP gid7Δ::kanMX6	This study	N/A
AK1273: YMaM330 BLM10-mCherry-sfGFP gid8Δ::kanMX6	This study	N/A
AK1274: YMaM330 BLM10-mCherry-sfGFP gid9Δ::kanMX6	This study	N/A
YMaM1091: YMaM330 MDH2-mCherry-sfGFP	This study	N/A
AK1276: YMaM330 MDH2-mCherry-sfGFP gid1Δ::kanMX6	This study	N/A
YMaM1127: YMaM330 MDH2-mCherry-sfGFP gid2Δ::kanMX6	This study	N/A
AK1277: YMaM330 MDH2-mCherry-sfGFP gid5Δ::kanMX6	This study	N/A
AK1278: YMaM330 MDH2-mCherry-sfGFP gid7Δ::kanMX6	This study	N/A
AK1279: YMaM330 MDH2-mCherry-sfGFP gid8Δ::kanMX6	This study	N/A
AK1280: YMaM330 MDH2-mCherry-sfGFP gid9Δ::kanMX6	This study	N/A
YMaM1094: YMaM330 YOR283W-mCherry-sfGFP	This study	N/A
AK1281: YMaM330 YOR283W-mCherry-sfGFP gid1Δ::kanMX6	This study	N/A
YMaM1130: YMaM330 YOR283W-mCherry-sfGFP gid2Δ::kanMX6	This study	N/A
AK1282: YMaM330 YOR283W-mCherry-sfGFP gid5Δ::kanMX6	This study	N/A
AK1283: YMaM330 YOR283W-mCherry-sfGFP gid7Δ::kanMX6	This study	N/A
AK1284: YMaM330 YOR283W-mCherry-sfGFP gid8Δ::kanMX6	This study	N/A
AK1285: YMaM330 YOR283W-mCherry-sfGFP gid9Δ::kanMX6	This study	N/A
AK1253: YMaM330 HSM3-mCherry-sfGFP gid4Δ::kanMX6	This study	N/A
YKEK067: YMaM330 HSM3-mCherry-sfGFP gid10Δ::kanMX6	This study	N/A
YKEK084: YMaM330 HSM3-mCherry-sfGFP ylr149cΔ::kanMX6	This study	N/A
AK1259: YMaM330 GPM3-mCherry-sfGFP gid4Δ::kanMX6	This study	N/A
YKEK068: YMaM330 GPM3-mCherry-sfGFP gid10Δ::kanMX6	This study	N/A
YKEK085: YMaM330 GPM3-mCherry-sfGFP ylr149cΔ::kanMX6	This study	N/A
YMaM1145: YMaM330 CPA1-mCherry-sfGFP gid4Δ::kanMX6	This study	N/A
YKEK069: YMaM330 CPA1-mCherry-sfGFP gid10Δ::kanMX6	This study	N/A
AK1335: YMaM330 CPA1-mCherry-sfGFP ylr149cΔ::hphNT1	This study	N/A
AK1338: YMaM330 CPA1-mCherry-sfGFP gid2Δ::kanMX6 ylr149cΔ::hphNT1	This study	N/A
YMaM1146: YMaM330 PHM8-mCherry-sfGFP gid4Δ::kanMX6	This study	N/A
YKEK070: YMaM330 PHM8-mCherry-sfGFP gid10Δ::kanMX6	This study	N/A
AK1336: YMaM330 PHM8-mCherry-sfGFP ylr149cΔ::hphNT1	This study	N/A
AK1339: YMaM330 PHM8-mCherry-sfGFP gid2Δ::kanMX6 ylr149cΔ::hphNT1	This study	N/A
AK1275: YMaM330 BLM10-mCherry-sfGFP gid4Δ::kanMX6	This study	N/A
YKEK071: YMaM330 BLM10-mCherry-sfGFP gid10Δ::kanMX6	This study	N/A
YKEK088: YMaM330 BLM10-mCherry-sfGFP ylr149cΔ::kanMX6	This study	N/A
YMaM1148: YMaM330 MDH2-mCherry-sfGFP gid4Δ::kanMX6	This study	N/A
YKEK072: YMaM330 MDH2-mCherry-sfGFP gid10Δ::kanMX6	This study	N/A
AK1337: YMaM330 MDH2-mCherry-sfGFP ylr149cΔ::hphNT1	This study	N/A
AK1340: YMaM330 MDH2-mCherry-sfGFP gid2Δ::kanMX6 ylr149cΔ::hphNT1	This study	N/A
AK1286: YMaM330 YOR283W-mCherry-sfGFP gid4Δ::kanMX6	This study	N/A
YKEK073: YMaM330 YOR283W-mCherry-sfGFP gid10Δ::kanMX6	This study	N/A
YKEK090: YMaM330 YOR283W-mCherry-sfGFP ylr149cΔ::kanMX6	This study	N/A
AK1240: BY4741 gid2Δ::kanMX6	This study	N/A
AK1241: BY4741 gid9Δ::kanMX6	This study	N/A
AK1243: BY4741 gid4Δ::kanMX6	This study	N/A
AK1245: BY4741 moh1Δ::kanMX6	This study	N/A
AK1244: BY4741 ipf1Δ::kanMX6	This study	N/A
AK1242: BY4741 ubc8Δ::kanMX6	This study	N/A
YMaM1205: Y7092 can1Δ::STE3pr-LEU2-GAL1pr-NLS-I-SCEI	Meurer et al., 2018	N/A
YJJF0017: Y7092 can1Δ::STE3pr-SpHIS5-TEFterm-GAL1pr-NLS-I-SCEI	This study	N/A
YLZY0017: YJJF0017 CPA1-hph	This study	N/A
YLZY0002: YJJF0017 CPA1-mCherry-sfGFP-hph	This study	N/A
YLZY0007: YJJF0017 CPA1-mCherry-sfGFP-hph gid2Δ::natNT2	This study	N/A
YLZY0006: YJJF0017 CPA1-mCherry-sfGFP-hph ubp14Δ::natNT2	This study	N/A
YLZY0013: YJJF0017 CPA1-mCherry-sfGFP-hph gid2Δ::natNT2 ubp14Δ::kanMX6	This study	N/A
YLZY0018: YJJF0017 PHM8-hph	This study	N/A
YLZY0003: YJJF0017 PHM8-mCherry-sfGFP-hph	This study	N/A
YLZY0009: YJJF0017 PHM8-mCherry-sfGFP-hph gid2Δ::natNT2	This study	N/A
YLZY0008: YJJF0017 PHM8-mCherry-sfGFP-hph ubp14Δ::natNT2	This study	N/A
YLZY0015: YJJF0017 PHM8-mCherry-sfGFP-hph gid2Δ::natNT2 ubp14Δ::kanMX6	This study	N/A
CPA1_tFT library: YMaM330 CPA1-mCherry-SceIsite-SpCYC1term-ScURA3-SceIsite-mCherryΔN-sfGFP	Khmelinskii et al., 2014	N/A
PHM8_tFT library: YMaM330 PHM8-mCherry-SceIsite-SpCYC1term-ScURA3-SceIsite-mCherryΔN-sfGFP	Khmelinskii et al., 2014	N/A
MDH2_tFT library: YMaM330 MDH2-mCherry-SceIsite-SpCYC1term-ScURA3-SceIsite-mCherryΔN-sfGFP	Khmelinskii et al., 2014	N/A
KO library: BY4743 ORF/orfΔ::kanMX	Winzeler et al., 1999	N/A
YKEK114: YMaM330 gid9Δ::kanMX	This study	N/A
YKEK115: YMaM330 gid4Δ::kanMX	This study	N/A
YKEK075: YMaM330 gid10Δ::kanMX	This study	N/A
YKEK092: YMaM330 ylr149cΔ::kanMX	This study	N/A
YKEK146: YMaM330 YLR149C-3HA-kanMX4	This study	N/A
YLZY0089: YMaM330 gid5Δ::hphNT1	This study	N/A
YMaM1083: YMaM330 GID1-mCherry-sfGFP	This study	N/A
YLZY0091: YMaM330 GID1-mCherry-sfGFP gid5Δ::hphNT1	This study	N/A
YMaM1075: YMaM330 GID8-mCherry-sfGFP	This study	N/A
YMaM1085: YMaM330 GID5-mCherry-sfGFP	This study	N/A
YMaM1088: YMaM330 GID7-mCherry-sfGFP	This study	N/A
YMaM1100: YMaM330 UBC8-mCherry-sfGFP	This study	N/A
YKEK148: YMaM330 CPA1(S2A)-mCherry-sfGFP	This study	N/A
YKEK149: YMaM330 CPA1(S2G)-mCherry-sfGFP	This study	N/A
YKEK151: YMaM330 CPA1(S2A)-mCherry-sfGFP gid9Δ::kanMX6	This study	N/A
YKEK152: YMaM330 CPA1(S2G)-mCherry-sfGFP gid9Δ::kanMX6	This study	N/A
YKEK160: YMaM330 CPA1(S2A)-mCherry-sfGFP ylr149cΔ::hphNT1	This study	N/A
YKEK161: YMaM330 CPA1(S2G)-mCherry-sfGFP ylr149cΔ::hphNT1	This study	N/A
YKEK196: YMaM330 BLM10(T2A)-mCherry-sfGFP	This study	N/A
YKEK197: YMaM330 BLM10(T2G)-mCherry-sfGFP	This study	N/A
YKEK199: YMaM330 BLM10(T2A)-mCherry-sfGFP gid9Δ::kanMX6	This study	N/A
YKEK200: YMaM330 BLM10(T2G)-mCherry-sfGFP gid9Δ::kanMX6	This study	N/A
YKEK202: YMaM330 BLM10(T2A)-mCherry-sfGFP ylr149cΔ::hphNT1	This study	N/A
YKEK203: YMaM330 BLM10(T2G)-mCherry-sfGFP ylr149cΔ::hphNT1	This study	N/A
GID_hits array: YMaM330 ORF-mCherry-SceIsite-SpCYC1term-ScURA3-SceIsite-mCherryΔN-sfGFP	This study	N/A
YKEK178: BY4741 gid10Δ::kanMX6	This study	N/A
YKEK179: BY4741 ylr149cΔ::kanMX6	This study	N/A

**Recombinant DNA**		

pFA6a-kanMX6	Wach et al., 1994	N/A
pFA6a-hphNT1	Janke et al., 2004	Euroscarf: P30347
pFA6a-natNT2	Janke et al., 2004	Euroscarf: P30346
pYM1: pFA6a-3HA-kanMX4	Knop et al., 1999	N/A
pRS413-GPDpr-MDH2-mCherry-sfGFP	This study	N/A
pRS413-GPDpr-MDH2(P2A)-mCherry-sfGFP	This study	N/A
pRS413-GPDpr-MDH2(P2G)-mCherry-sfGFP	This study	N/A
pRS413-GPDpr-PHM8-mCherry-sfGFP	This study	N/A
pRS413-GPDpr-PHM8(T2A)-mCherry-sfGFP	This study	N/A
pRS413-GPDpr-PHM8(T2G)-mCherry-sfGFP	This study	N/A
pRS413-GPDpr	This study	N/A
pRS413-GPDpr-YLR149C	This study	N/A
pRS413-GPDpr-HA-YLR149C	This study	N/A
pRS413-GPDpr-HA-YLR149C(Δ727-730)	This study	N/A
pRS413-GPDpr-HA-YLR149C(Y727A L729A)	This study	N/A
pRS413-GPDpr-HA-YLR149C(Y727D L729D)	This study	N/A
pRS316	Sikorski and Hieter, 1989	N/A
pRS316-GID5	This study	N/A
pRS316-GID5(W606A H610A Y613A)	This study	N/A
pRS316-GID5(W606A Y613A Q649A)	This study	N/A
pRS413-GPDpr-GPM3-mCherry-sfGFP	This study	N/A
pRS413-GPDpr-GPM3(T2A)-mCherry-sfGFP	This study	N/A
pRS413-GPDpr-GPM3(T2G)-mCherry-sfGFP	This study	N/A
pRS413-GPDpr-YOR283W-mCherry-sfGFP	This study	N/A
pRS413-GPDpr-YOR283W(T2A)-mCherry-sfGFP	This study	N/A
pRS413-GPDpr-YOR283W(T2G)-mCherry-sfGFP	This study	N/A
pRS413-GPDpr-HA-Luc	This study	N/A
pRS413-GPDpr-HA-Luc-GID11^C20^	This study	N/A
pRS413-GPDpr-HA-Luc-GID4^C20^	This study	N/A
pRS413-GPDpr-PHM8(HSM3^2-5^)-mCherry-sfGFP	This study	N/A
pRS413-GPDpr-PHM8(GPM3^2-5^)-mCherry-sfGFP	This study	N/A
pRS413-GPDpr-PHM8(CPA1^2-5^)-mCherry-sfGFP	This study	N/A
pRS413-GPDpr-PHM8(BLM10^2-5^)-mCherry-sfGFP	This study	N/A
pRS413-GPDpr-PHM8(MDH2^2-5^)-mCherry-sfGFP	This study	N/A
pRS413-GPDpr-PHM8(YOR283W^2-5^)-mCherry-sfGFP	This study	N/A

**Software and algorithms**		

ImageJ	Schneider et al., 2012	https://imagej.nih.gov/ij/
ImageLab	Bio-Rad	http://www.bio-rad.com/en-us/product/image-lab-software?ID=KRE6P5E8Z
R	R Foundation for Statistical Computing	https://www.R-project.org/
Phobius	Käll et al., 2004	N/A
TMHMM	Krogh et al., 2001	N/A
InterPro database	Mitchell et al., 2019	N/A
SignalP	Petersen et al., 2011	N/A
MUSCLE	Edgar, 2004	https://www.ebi.ac.uk/Tools/msa/muscle/
JalView	Waterhouse et al., 2009	https://www.jalview.org/
Revigo	Supek et al., 2011	http://revigo.irb.hr/

### Resource availability

#### Lead contact

Information and requests for resources and reagents should be directed to the Lead Contact, Anton Khmelinskii (a.khmelinskii@imb-mainz.de).

#### Materials availability

All unique/stable reagents generated in this study are available from the Lead Contact with a completed Materials Transfer Agreement.

#### Data and code availability

The R vignette reproducing the analysis of the screens profiling the ubiquitin-proteasome system and containing the screen dataset is deposited in the heiData repository:

https://heidata.uni-heidelberg.de/citation?persistentId=doi:10.11588/data/Q3TSLH

Unprocessed immunoblot images are available at the Mendeley Data repository:

https://data.mendeley.com/datasets/rv8f9bp5b2

### Experimental model and subject details

All yeast strains used in this study are listed in the Key resources table and are derivatives of BY4741, Y8205, Y7092 or BY4743. Yeast genome manipulations (gene tagging and gene deletion) were performed using PCR targeting and lithium acetate transformation (Janke et al., 2004). All experiments were performed at 30°C in synthetic complete (SC) medium with 2% (w/v) glucose as carbon source, unless stated otherwise.

### Method details

#### Immunoblotting

(i) For log phase experiments with *MCR1-TAP* and *MCR1-3xHA* strains, cells were grown to 6x10^6^-1x10^7^ cells/ml in SC medium with 2% (w/v) glucose. 1 mL samples were mixed with 150 μL of 1.85 M NaOH and 10 μL of 2-mercaptoethanol and flash-frozen in liquid nitrogen.
(ii) For glucose starvation experiments with *MCR1-TAP* and *MCR1-3xHA* strains, cells from a dense pre-culture were inoculated into SC medium with 0.1% (w/v) glucose to a density of 1x10^6^ cells/ml and grown for 48 h to 4x10^7^ cells/ml. 250 μL samples were processed as above.
(iii) For proteasome inhibition experiments with the *MCR1-TAP pdr5*Δ strain, log phase cultures were treated with MG132 (BML-PI102-0025, Enzo Life Sciences) to 80 μg/ml final concentration or DMSO as control for 90 min, followed by cell harvesting as above.
(iv) For conditional analysis of Gid11-HA expression, cells were first grown to 8x10^6^-1x10^7^ cells/ml in SC medium with 2% (w/v) glucose. Then, 10% of the culture was harvested as control, while the remaining was washed once with water and resuspended in growth media with different compositions: (1) carbon starvation medium (SC without carbon sources), (2) SC with 2% (v/v) ethanol, (3) SC with 2% (w/v) glucose, (4) SC with 2% (v/v) glycerol, (5) amino acid starvation medium (SC with 2% (w/v) glucose but without amino acids), (6) nitrogen starvation medium (SC with 2% (w/v) glucose but without nitrogen base), (7) osmotic stress medium (SC with 2% (w/v) glucose and 1.4 M NaCl) and (8) SC with 2% (w/v) glucose and 1 M KH_2_PO_4_. Cells were incubated at 30°C (also 37°C for cells resuspended in SC glucose) for 3 h before harvesting. In addition, yeast colonies were harvested from an agar plate incubated at 30°C for 48 h.

For (i) and (ii), samples were thawed on ice and whole cell protein extracts were prepared by precipitation with 150 μL of 55% (w/v) of trichloroacetic acid, followed by centrifugation to remove the supernatant. The pellet was resuspended in 50-100 μL of HU buffer (8 M urea, 5% SDS, 200 mM Tris-HCl pH 6.8, 1 mM EDTA, 1.5% DTT and phenol blue as coloring and pH indicator) per 1x10^7^ cells (Knop et al., 1999), followed by SDS-PAGE and immunoblotting. For TAP-tagged strains, membranes were probed with rabbit peroxidase anti-peroxidase (PAP) antibodies (Z0113, Dako). For HA-tagged strains, membranes were probed with mouse anti-HA antibodies (12CA5) and HRP-conjugated goat anti-mouse antibodies (Dianova 115-035-003). Membranes were imaged on a LAS-4000 system (Fuji). Quantification was performed in ImageJ (Schneider et al., 2012).

For (iii) and (iv), samples were processed as above, followed by SDS-PAGE and immunoblotting. Membranes were probed with rabbit peroxidase anti-peroxidase (PAP) antibodies (P1291, Sigma-Aldrich) to detect Mcr1-TAP, or with mouse anti-HA antibodies (12CA5) followed by HRP-conjugated anti-mouse antibodies (G-21040, Thermo Fisher Scientific) to detect Gid11-HA. The loading control Pgk1 was detected using mouse anti-Pgk1 antibodies (459250, Thermo Fisher Scientific) followed by the same anti-mouse secondary antibodies. The ChemiDoc MP imaging system (Bio-Rad) was used to image the membranes after addition of the Pierce ECL Plus Western Blotting Substrate (32132, Thermo Fisher Scientific). Quantification was performed using ImageLab (Bio-Rad).

#### Flow cytometry

Strains were grown to saturation in 96-well plates, diluted into fresh medium, and grown for 8 h to 2-8 × 10^6^ cells/ml. Fluorescence measurements were performed on a BD FACSCanto RUO (BD Biosciences) equipped with a high-throughput sampler loader, a 488-nm laser with a combination of 505 nm long-pass and 530/30 nm band pass emission filters for sfGFP detection and a 561 nm laser with a combination of 600 nm long-pass and 610/20 nm band pass emission filters for mCherry detection. Populations were gated for single cells in the G1 phase of the cell cycle using the first peak in the side scatter width (SSC-W) histogram. At least 13000 cells were analyzed for each strain. Median intensities of cellular autofluorescence were subtracted from each channel and the mCherry/sfGFP ratio was calculated.

#### Measurements of proteome abundance and turnover with the tFT library

The tFT library, taken through marker excision in the tagged loci (Khmelinskii et al., 2011), was arranged in 1536-colony format using a pinning robot (RoToR, Singer Instruments), with 4 technical replicates of each tFT-tagged strain next to replicates of an untagged control strain, dummy colonies on the outer rows and columns to minimize the influence of nutrient access on colony size and fluorescence and, on each plate, a set of reference strains spanning the full range of protein abundances and stabilities in the tFT library. Fluorescence intensities of the final colonies were measured after 24 h of growth using Infinite M1000 or Infinite M1000 Pro plate readers (Tecan) equipped with stackers for automated plate loading (Tecan) and custom temperature control chambers. Measurements in mCherry (587/10 nm excitation, 610/10 nm emission, optimal detector gain) and sfGFP (488/10 nm excitation, 510/10 nm emission, optimal detector gain) channels were performed at 400 Hz frequency of the flash lamp, with ten flashes averaged for each measurement.

After removing measurements of border colonies, mCherry and sfGFP fluorescence intensities were corrected for spatial effects by robust local regression using measurements of the control strain, corrected for cellular autofluorescence by subtracting the median of the control strain on each plate, log-transformed and scaled across plates by the median of the reference strains on each plate. Technical replicates were summarized by taking the median. Distribution of fluorescence intensities and mCherry/sfGFP ratios were median-centered.

For the gene ontology analysis, all proteins tagged in the tFT library were mapped to GO Slim terms. GO terms with 200 or fewer proteins were subsequently removed. In addition, GO terms with high variability in log_2_(mCherry/sfGFP) were excluded. For unbiased identification of GO terms with high variability, the dependence of log_2_(mCherry/sfGFP) median absolute deviation (MAD) on median log_2_(mCherry/sfGFP) per term was removed by linear regression, and terms with corrected MAD(log_2_(mCherry/sfGFP)) above an arbitrary threshold of 0.1 were subsequently removed. The result was filtered for redundant GO Slim terms and visualized using Revigo (Supek et al., 2011).

#### Profiling of the ubiquitin-proteasome system

Each strain in the tFT library (Khmelinskii et al., 2014) was crossed to the UPS array (Table S2) using synthetic genetic array (SGA) methodology (Baryshnikova et al., 2010b; Tong et al., 2001). Crosses were performed in 1536-colony format, with 4 technical replicates placed next to each other. Screens were conducted in batches of 192 tFT queries. Two queries (*UBI4-tFT* and *YJR096W-tFT*) were repeated in every batch (Figure S2A). Each screen plate consisted of two queries crossed to the UPS array and a set of strains spanning the full range of protein abundances and stabilities in the tFT library, used as a reference across all plates. Mating, sporulation, selection of haploids carrying both a tFT-tagged allele and a mutant allele, followed by marker excision in the tFT-tagged locus were performed by sequential pinning of yeast colonies on appropriate selective media using pinning robots (BioMatrix, S&P Robotics) (Baryshnikova et al., 2010b; Khmelinskii et al., 2011). Plates were photographed to determine colony sizes. Fluorescence intensities of the final colonies were measured after 24 h of growth at 30°C as detailed above. This temperature was chosen to only partially inhibit growth of all temperature-sensitive mutants. Measurements were filtered for failed crosses based on colony size after haploid selection. Fluorescence intensity measurements were log-transformed and median effects for each tFT query were subtracted. Spatial effects on plates were corrected by local regression. The UPS array contained two negative control strains (*ura3*Δ::*kanMX* and *his3*Δ::*kanMX*; Table S2). Absolute fluorescence intensities of all tFT queries in the wild-type background were scaled across plates using the reference strains before calculating mCherry/sfGFP ratios. For each tFT query, corrected sfGFP and mCherry intensities in each mutant were compared to the mean of negative controls. For each mutant, the dependence of changes in fluorescence on absolute fluorescence intensity and screen order was removed by a local polynomial fit. The same correction was applied to mCherry/sfGFP ratios. Finally, a moderated t test implemented in the R/Bioconductor package limma (Ritchie et al., 2015) was used to test for interaction effects and to compute p values, adjusted for multiple testing using the method of Benjamini-Hochberg.

Stability measurements of individual proteins in low throughput (Figures 3C, 5C, 5D, 5G, S6G, S6H, S7B–S7D, S7I, and S7J) were performed with strains constructed independently (Key resources table) or obtained through independent crosses using identical procedures on a pinning robot (RoToR, Singer Instruments). For each tFT-tagged protein, fluorescence intensities of colonies were corrected for autofluorescence, using measurements from neighboring negative controls, and normalized for plate effects, using measurements from neighboring wild-type colonies.

#### Genome-wide screens for factors affecting protein stability

tFT query strains (*MDH2-tFT*, *CPA1-tFT* and *PHM8-tFT*, Key resources table) were crossed with a heterozygous diploid genome-wide library of yeast gene deletion mutants (Winzeler et al., 1999). Crosses were performed in 1536-colony format, with 4 technical replicates of each cross arranged next to each other. Mating, sporulation, selection of haploids carrying both a tFT-tagged allele and a gene deletion, followed by marker excision in the tFT-tagged locus were performed by sequential pinning of yeast colonies on appropriate selective media using a pinning robot (RoToR, Singer Instruments) (Baryshnikova et al., 2010b; Khmelinskii et al., 2011). Plates were photographed to determine colony sizes. Fluorescence intensities of the final colonies were measured after 24 h of growth as detailed above. Measurements of colony size after haploid selection, corrected for spatial effects by local regression, were used to identify and remove failed crosses or measurements from empty positions on the plates. Fluorescence intensity measurements were log-transformed and corrected for spatial effects before calculating mCherry/sfGFP ratios. For each query, changes in protein abundance (sfGFP intensity) and stability (mCherry/sfGFP ratio) were estimated by calculating z-scores (Dederer et al., 2019). Technical replicates were summarized by calculating the mean and standard deviation. P values were computed using a t test and adjusted for multiple testing using the method of Benjamini-Hochberg.

#### Analysis of Gid11 conservation

Gid11 homologs in different yeast species were obtained from Huerta-Cepas et al. (2014) and Wapinski et al. (2007) or identified by sequence homology to *S. cerevisiae* Gid11 (Table S7). The topology of the evolutionary tree was adapted from Dujon (2010). Multiple sequence alignment of putative Gid11 sequences was performed using MUSCLE (Edgar, 2004) and a histogram of alignment conservation was calculated with JalView (Waterhouse et al., 2009).

#### Co-immunoprecipitation

Strains were grown in 100 mL SC medium lacking histidine with 2% (w/v) glucose to ∼1x10^7^ cells/ml. Cells were harvested by centrifugation and lysed in 600 μL of IP buffer (50 mM Tris-HCl pH 7.5, 100 mM NaCl, 1% (v/v) Triton X-100, 2 mM EDTA and protease inhibitors (4693159001, Sigma-Aldrich)) by vortexing at 4°C in the presence of acid-washed glass beads. Lysates were centrifuged at 4°C for 10 min and 400 μL of supernatant was collected, of which 300 μL was then added to 60 μL of Dynabeads M-280 Sheep Anti-Rabbit IgG (11203D, Thermo Fisher Scientific) previously conjugated with 1 μL of rabbit anti-GFP antibodies (ab290, Abcam). Immunoprecipitation of tFT-tagged proteins was allowed to proceed for 2 h at 4°C with gentle rocking. After that, beads were washed three times with 1 mL of IP buffer, resuspended in 50 μL of IP buffer plus 17 μL of 4X Laemmli SDS sample buffer (250 mM Tris-HCl pH 6.8, 8% (w/v) SDS, 40% (v/v) glycerol, 5% (v/v) 2-mercaptoethanol, and 0.1% (w/v) bromophenol blue), and incubated at 99°C for 10 min, followed by SDS-PAGE and immunoblotting as described above. Input samples were prepared by mixing 30 μL of lysates with 20 μL of IP buffer and 17 μL of 4X Laemmli SDS sample buffer and processed similarly. Membranes were probed with mouse anti-GFP antibodies (11814460001, Sigma-Aldrich) followed by HRP-conjugated anti-mouse antibodies (G-21040, Thermo Fisher Scientific) to detect tFT-tagged proteins, or with mouse anti-HA antibodies (12CA5) followed by the same secondary antibodies to detect the presence of any co-immunoprecipitated HA-tagged proteins.

### Quantification and statistical analysis

Quantification and statistical analysis procedures are detailed for every experiment in Method details. Bar plots throughout the manuscript represent mean ± standard deviation (n ≥ 3).

## Supplemental information

Document S1. Figures S1–S7

Table S1. Yeast proteome abundance and stability measured with the tFT library, related to Figure 1

Table S2. List of mutants in the UPS array, related to Figure 2

Table S3. List of significant changes in protein abundance and stability in UPS mutants, related to Figure 2

Table S4. List of significant correlations of proteome turnover profiles, related to Figure 4

Table S5. N-terminal sequences of potential GID substrates, related to Figure 5

Table S6. Abundance and stability of Mdh2-tFT, Cpa1-tFT, and Phm8-tFT in a genome-wide library of yeast gene knockouts, related to Figure 6

Table S7. Sequences of putative Gid11 homologs in different yeast species, related to Figure 6

Document S2. Article plus supplemental information
