# Supplementary material for: Timer-based proteomic profiling of the ubiquitin-proteasome system reveals a substrate receptor of the GID ubiquitin ligase
Source: Mol Cell. 2021 Jun 3;81(11):2460–2476.e11. doi: 10.1016/j.molcel.2021.04.018 (PMC8189435; doi:10.1016/j.molcel.2021.04.018)
Supplement: Document S1. Figures S1–S7 [file mmc1.pdf]

Molecular Cell, Volume *81*

Supplemental information

Timer-based proteomic profiling

# of the ubiquitin-proteasome system reveals

a substrate receptor of the GID ubiquitin ligase

Ka-Yiu Edwin Kong, Bernd Fischer, Matthias Meurer, Ilia Kats, Zhaoyan Li, Frank Rühle, Joseph D. Barry, Daniel Kirrmaier, Veronika Chevyreva, Bryan-Joseph San Luis, Michael Costanzo, Wolfgang Huber, Brenda J. Andrews, Charles Boone, Michael Knop, and Anton Khmelinskii

Figure S1

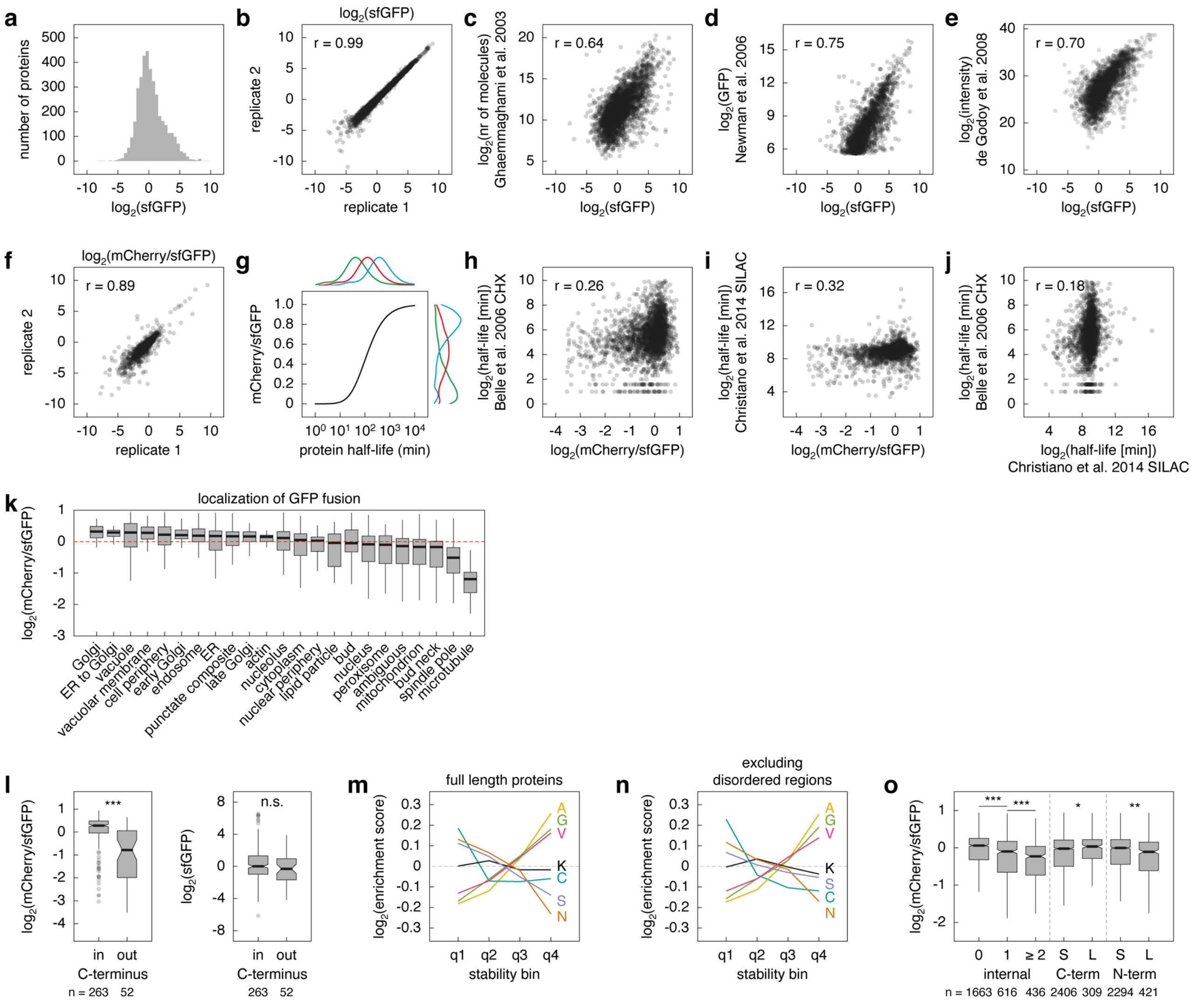

Figure S1. Measurements of proteome abundance and turnover with the tFT library, related to Figure 1

a – Distribution of protein abundance in the yeast proteome, determined using the tFT library. sfGFP fluorescence measurements of 4004 strains expressing different proteins C-terminally tagged with the mCherry-sfGFP timer at their endogenous chromosomal loci, grown on agar medium with 2 biological replicates each with 4 technical replicates per protein, summarized by their median value (Table S1).
b – Comparison of sfGFP fluorescence intensities between two biological replicates of the tFT library.
c, **d**, **e** – Comparison between protein abundance estimates with the tFT library and protein abundance measurements by (**c**) immunoblotting of strains expressing TAP-tagged proteins (Ghaemmaghami et al., 2003), (**d**) flow cytometry of strains expressing GFP-tagged proteins (Newman et al., 2006) and (**e**) mass spectrometry (de Godoy et al., 2008).
f – Comparison of protein stability estimates between two biological replicates of the tFT library.
g – Theoretical relationship between the mCherry/sfGFP ratio and protein half-life calculated using experimentally determined maturation parameters of mCherry and sfGFP (Khmelinskii et al., 2012). To assess if the non-linear dependence of the mCherry/sfGFP ratio on protein half-life could affect the distribution of mCherry/sfGFP ratios in the tFT library, distributions of mCherry/sfGFP ratios (right) were calculated for three distributions of protein half-lives (top, red curve – distribution of protein half-lives in *S. cerevisiae* experimentally determined by cycloheximide chases of strains expressing TAP-tagged proteins, with an average half-life of ~43 min (Belle et al., 2006), green and blue – shifts of the red curve towards faster or slower average protein turnover, respectively). The distribution of mCherry/sfGFP ratios in the tFT library could be skewed towards low mCherry/sfGFP ratios if the average half-life of yeast proteins is significantly higher than ~43 min (blue curves).
h, **i** – Comparison between protein stability estimates with the tFT library and (**h**) protein stability measurements by cycloheximide chase of strains expressing TAP-tagged proteins (Belle et al., 2006) or (**i**) using pulse-SILAC mass spectrometry (Christiano et al., 2014).
j – Comparison between protein stability measurements by cycloheximide chase of strains expressing TAP-tagged proteins (Ghaemmaghami et al., 2003) and measurements using pulse-SILAC mass spectrometry (Christiano et al., 2014).

k – mCherry/sfGFP ratios of proteins with different subcellular localizations, determined by fluorescence microscopy of strains expressing GFP-tagged proteins (Huh et al., 2003). Dashed line marks the median of mCherry/sfGFP ratios in the tFT library.

l – mCherry/sfGFP ratios (left) and sfGFP intensities (right) of secretory proteins with the C-terminus located in the cytosol (in) or in the ER lumen (out) (Kim et al., 2006). ***, p-value < 0.001; n.s., not significant in a Wilcoxon rank-sum test. Differences in local environment between ER lumen and cytosol likely contribute to the observed difference in mCherry/sfGFP ratios between the two groups of proteins.

m, n – Enrichment scores of the indicated amino acids (amino acid frequency in a selected group relative to the whole tFT library) for proteins in the tFT library split into four quantiles according to their mCherry/sfGFP ratios (q1 – lowest stability, q4 – highest stability), as indicated in Fig. 1b. Full protein sequences (**m**) or sequences excluding long disordered segments (**n**) retrieved from (van der Lee et al., 2014) were considered.

o – mCherry/sfGFP ratios of proteins with different levels of disorder: 0, 1 or ≥ 2 internal disordered segments (defined as continuous stretches of ≥ 40 disordered residues), long (L) or short (S) disordered stretches (defined as stretches of ≤ 30 or > 30 disordered residues, respectively) at the N- or at the C-terminus (van der Lee et al., 2014). C-terminal disordered stretches considered here are in the context of native untagged proteins. Upon C-terminal tagging with the tFT, these disordered stretches become internal. *, **, ***, p-value < 0.05, 0.01, 0.001 in a Wilcoxon rank-sum test, respectively.

Figure S2

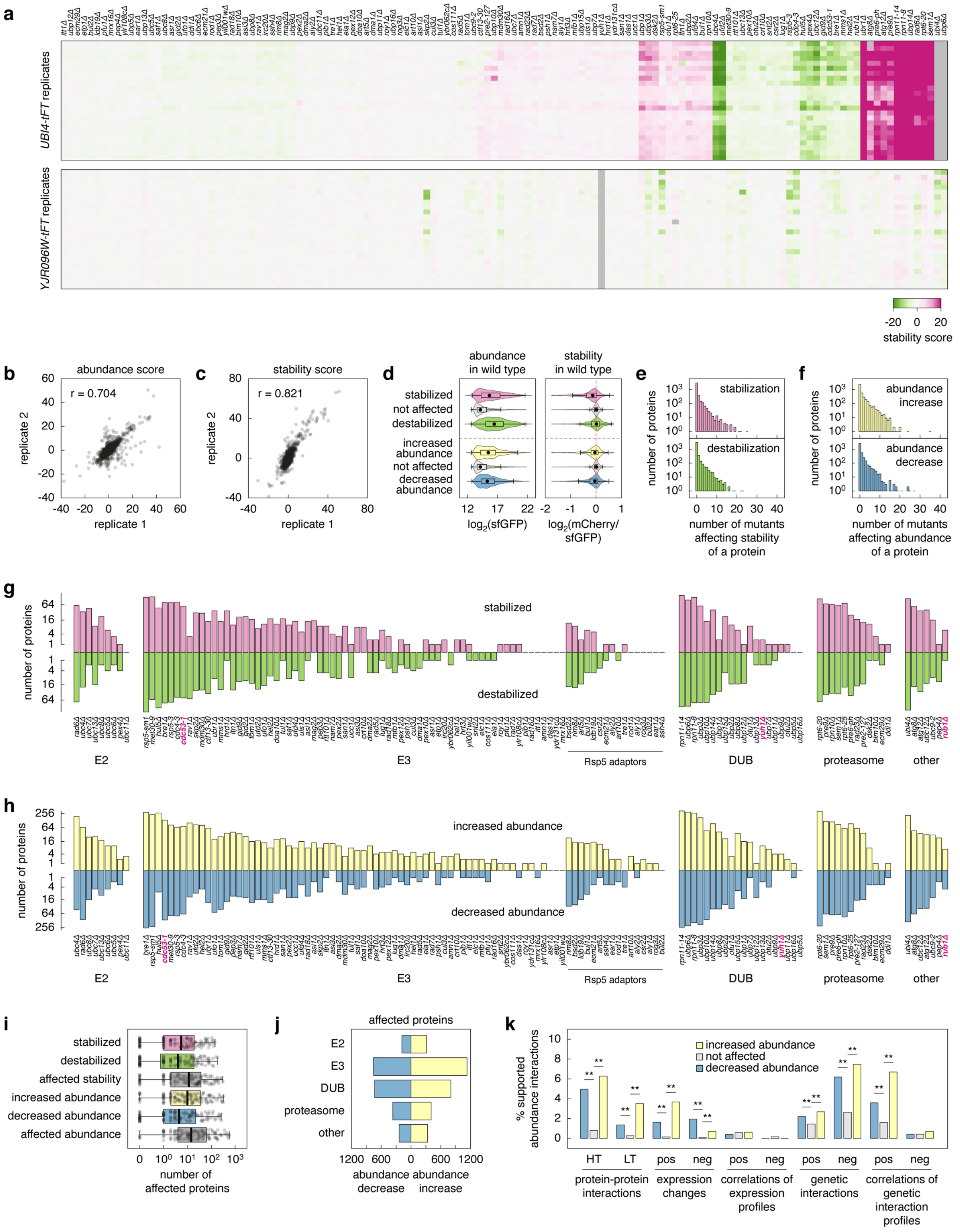

Figure S2. Overview of tFT-tagged proteins affected in mutants of UPS components, related to Figure 2

a – Reproducibility of screens to functionally profile the ubiquitin-proteasome system. Scaled changes in mCherry/sfGFP ratios (stability score) for two tFT queries, *UBI4-tFT* (top) and *YJR096W-tFT* (bottom), across all tested UPS mutants, assayed 24 times throughout the screens.

b, **c** – Reproducibility of screens to functionally profile the ubiquitin-proteasome system. Comparison of scaled changes in sfGFP intensities (abundance score, **b**) and mCherry/sfGFP ratios (stability score, **c**) for 96 tFT-tagged proteins that were independently screened against the UPS array in two biological replicates. Each data point represents the mean of 4 technical replicates.
d – Distributions of protein abundance (sfGFP levels) and stability (mCherry/sfGFP intensity ratio) of tFT-tagged proteins in the wild type background. Proteins were grouped according to their behavior in terms of abundance or stability in mutants of ubiquitin-proteasome system components. Proteins were considered to be significantly affected in terms of abundance or stability for mutant-tFT interactions with an absolute abundance or stability score > 4 at 1% false discovery rate (**d**-**j**).
e, **f** – Distributions of the number of mutants affecting stability (**e**) or abundance (**f**) of a protein for the 3806 tested tFT queries.
g, **h** – Number of proteins affected in terms of stability (**g**) or abundance (**h**) in the 132 mutants in the UPS array. *cdc53-1*, *yuh1*Δ, *rub1*Δ strains and mutants of Rsp5 adaptors are highlighted for clarity.
i – Box plot of the number of proteins affected in terms of stability or abundance in the 132 mutants in the UPS array. Centerlines mark the medians, box limits indicate the 25th and 75th percentiles, and whiskers extend to minimum and maximum values.
j – Distribution of the total number of proteins that increased or decreased in abundance in mutants of ubiquitin-conjugating enzymes (E2), ubiquitin-protein ligases (E3), deubiquitinating enzymes (DUB), proteasomal components (proteasome) or other factors (Table S2).
k – Overlap between abundance interactions and different types of interactions obtained from external datasets (STAR Methods). Abundance interactions were grouped according to impact of the mutant on the abundance of the tFT-tagged protein at 1% false discovery rate. pos – positive, neg – negative; **, p-value < 0.01 in a Fisher's exact test.

Figure S3

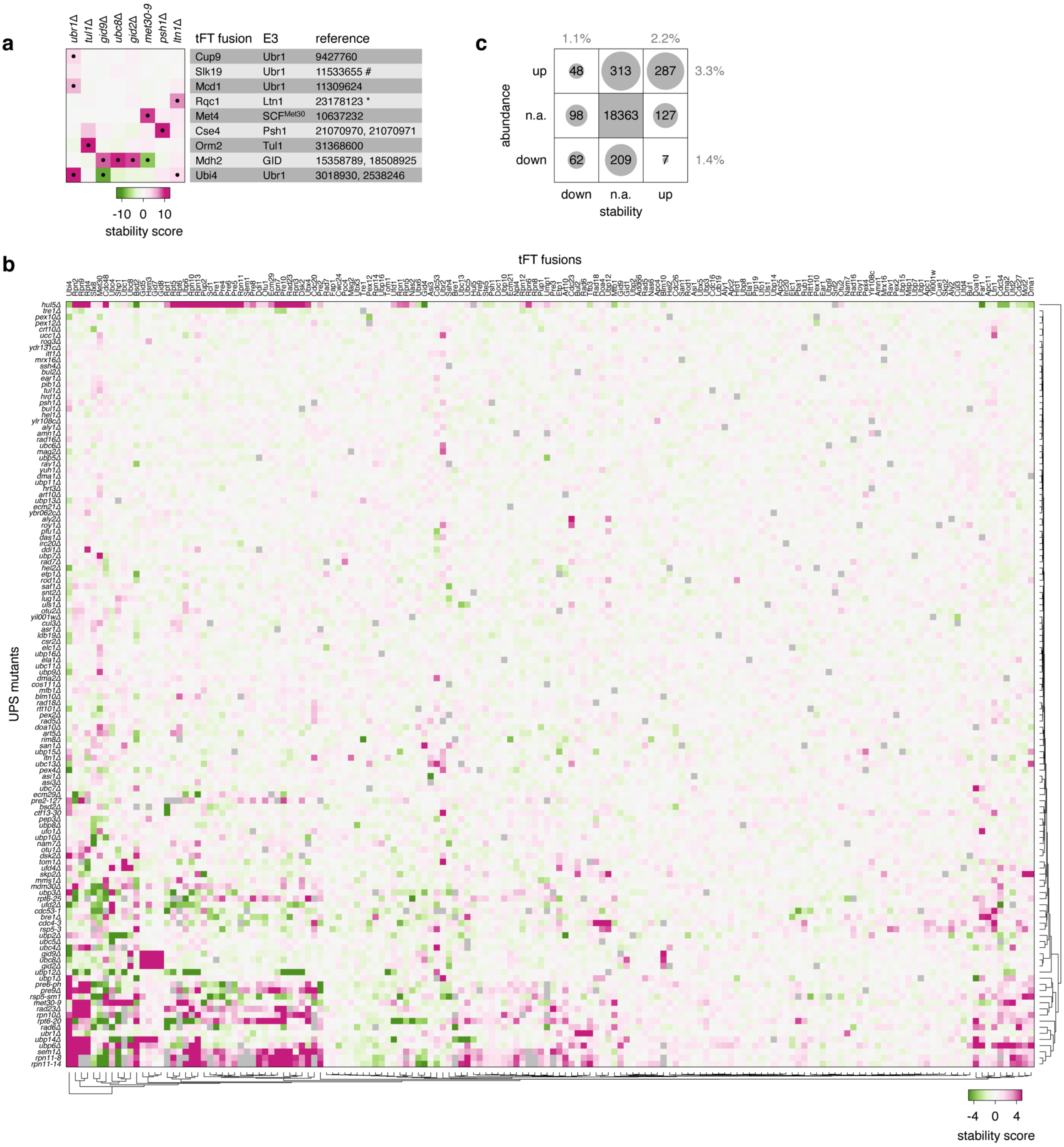

tFT fusion	E3	reference
Cup9	Ubr1	9427760
Slk19	Ubr1	11533655 #
Mcd1	Ubr1	11309624
Rqc1	Ltn1	23178123 *
Met4	SCF^{Met30}	10637232
Cse4	Psh1	21070970, 21070971
Orm2	Tul1	31368600
Mdh2	GID	15358789, 18508925
Ubi4	Ubr1	3018930, 2538246

Figure S3. Self-regulation in the UPS, related to Figure 2

a – Heatmap of protein stability changes for known substrates of various ubiquitin-protein ligases (E3) in the indicated UPS mutants. Stability scores are color-coded from green (stability decrease) to magenta (stability increase). Significant changes in protein stability at 1% false discovery rate are marked (●). For each substrate, the cognate E3 and the PubMed IDs of the studies describing the enzyme-substrate relationship are listed.
#, Slk19 was shown to not be a Ubr1 substrate. *, Rqc1 was suggested to be a potential Ltn1 substrate.
b – Heatmap of protein stability changes for tFT-tagged UPS components in the indicated UPS mutants. Stability scores are color-coded from green (stability decrease) to magenta (stability increase).
c – Summary of phenotypic outcomes in terms of protein abundance and stability for tFT-tagged UPS components across all tested mutant-tFT pairs at 1% false discovery rate. n.a. – protein abundance or stability not affected. The percentage of mutant-tFT pairs with each phenotype is indicated.

Figure S4

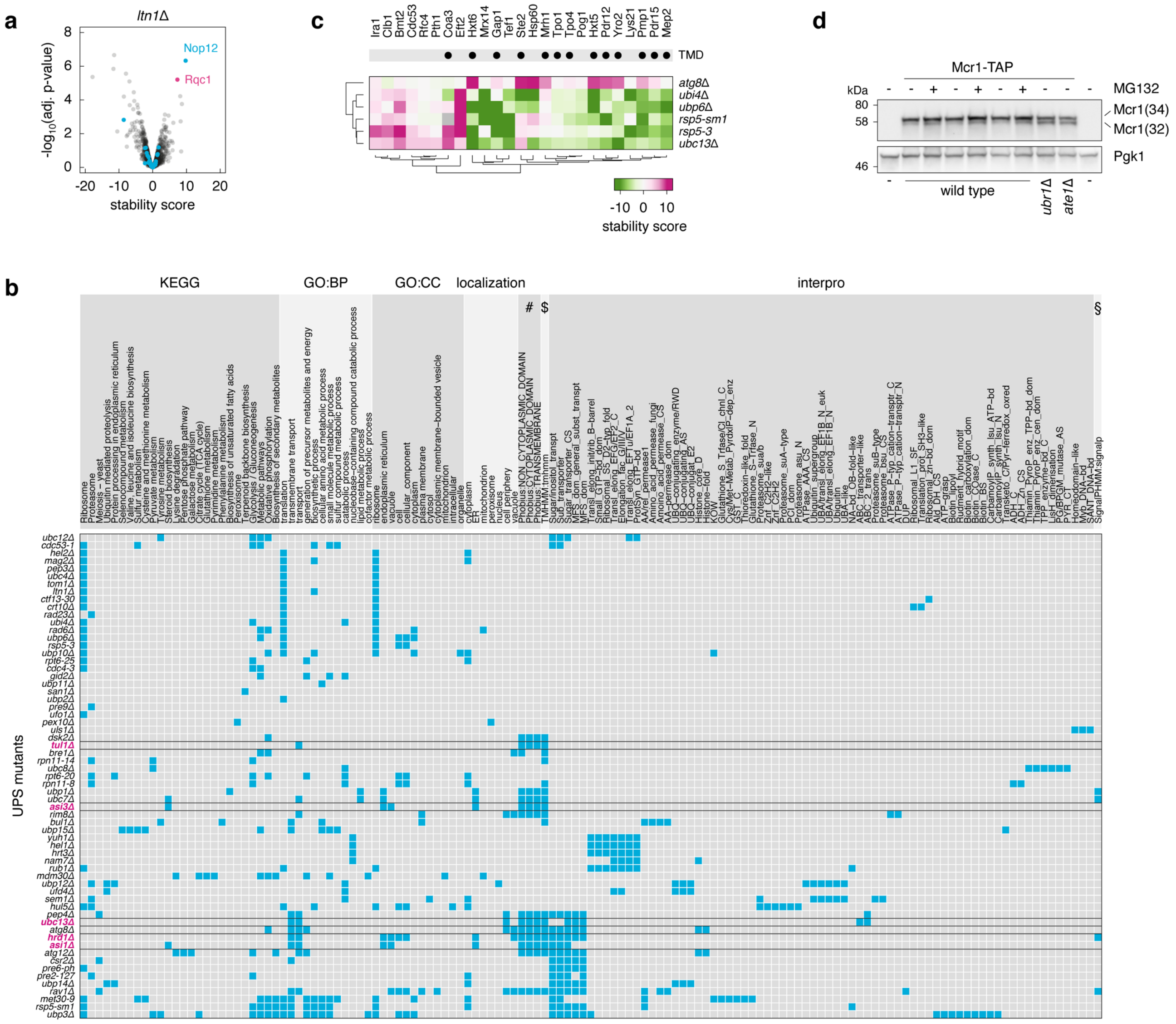

Figure S4. Turnover interactions inform on UPS functions, related to Figures 2 and 3

a – Volcano plot of changes in protein stability in the absence of the E3 Ltn1. Proteins containing a strong polybasic stretch (Brandman et al., 2012) are highlighted in blue.

b – Summary of gene set enrichment analysis for proteins with altered stability in UPS mutants. Blue – proteins in a given category are overrepresented among those affected by a given mutant, gray – no overrepresentation. KEGG – pathways in the Kyoto Encyclopedia of Genes and Genomes; GO:BP – biological process GO terms; GO Slim CC – cellular component GO terms; localization – protein localization as determined by microscopy of strains expressing GFP-tagged proteins (Huh et al., 2003); #, $ – proteins with predicted transmembrane domains using Phobius (Käll et al., 2004) or TMHMM (Krogh et al., 2001); interpro – protein families or domains in the InterPro database (Mitchell et al., 2019); § – proteins with predicted signals peptides using SignalP (Petersen et al., 2011). Only mutants with at least one significant association were included in the heatmap. Profiles of *tul1*Δ, *asi3*Δ, *ubc13*Δ, *hrd1*Δ and *asi1*Δ mutants are highlighted for clarity.

c – Summary heatmap of protein stability changes in the absence of the Ubc13 ubiquitin-conjugating enzyme. Stability scores are color-coded from green (stability decrease) to magenta (stability increase). Only proteins with a significant change in stability in the *ubc13*Δ mutant (1% false discovery rate and absolute stability score > 4) are shown. Proteins with transmembrane domains (TMDs) are indicated. Included for comparison are mutants with phenotypes correlated to that of *ubc13*Δ (Fig. 4a): positive correlation – *ubi4*Δ, *ubp6*Δ, *rsp5-sm1*, *rsp5-3* and negative correlation – *atg8*Δ. Because sfGFP has a higher pKa value than mCherry, the mCherry-sfGFP timer is pH-sensitive and the mCherry/sfGFP ratio increases with decreasing pH (Khmelinskii and Knop, 2014). Therefore, for transmembrane proteins degraded in the vacuole, e.g., Gap1, blocking protein degradation can result in a decrease in the mCherry/sfGFP ratio.

d – Accumulation of Mcr1(32) upon proteasome inhibition with MG132 for 90 min. Pgk1 was used as loading control.

Figure S5

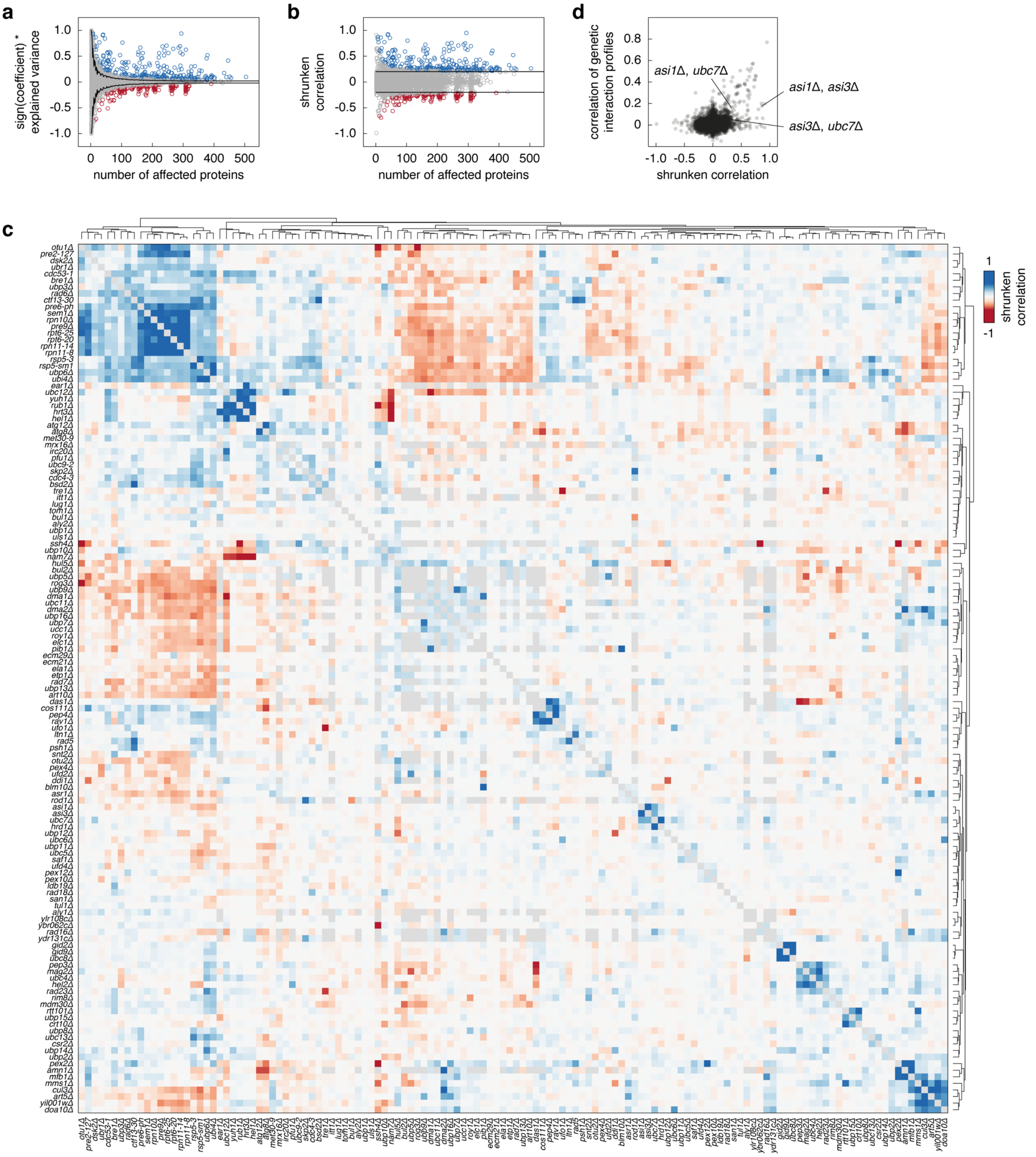

Figure S5. Correlation analysis of proteome turnover profiles, related to Figure 4

a, b – Calculation of correlations of protein turnover profiles. For each mutant, tFT-tagged proteins with significantly altered stability are selected. To assess if proteome turnover profiles of two mutants are correlated, a linear model is fitted on the union of sets of proteins affected in each mutant and p-values are adjusted by the method of Benjamini-Hochberg that controls for the false discovery rate. The explained variance is then used as a measure for the goodness-of-fit. Since a different number of proteins is used for each correlation, pairs of mutants with a small number of affected proteins can achieve a high value of explained variance while the p-value is still poor (**a**). Therefore, the explained variance was corrected for the number of affected proteins used in the test to obtain a shrunken correlation used hereafter (**b**) (STAR Methods). Red – significant negative correlations, blue – significant positive correlations (1% false discovery rate). The explained variance of negatively correlated pairs of mutants is multiplied by -1.

c – Annotated heatmap of shrunken correlations of proteome turnover profiles for all tested mutants in the UPS array.

d – Comparison of correlations of genetic interaction profiles between genes in the UPS array and shrunken correlations of proteome turnover profiles from **c**. For genetic interaction profiles, correlations are typically considered significant for Pearson correlation coefficients > 0.2 (Costanzo et al., 2016). Absolute shrunken correlation coefficients < 0.2 are not significant (**b**). Significant negative and some positive shrunken correlations are not supported by correlations of genetic interaction profiles, as exemplified by correlations between *asi1*Δ, *asi3*Δ and *ubc7*Δ mutants.

Figure S6

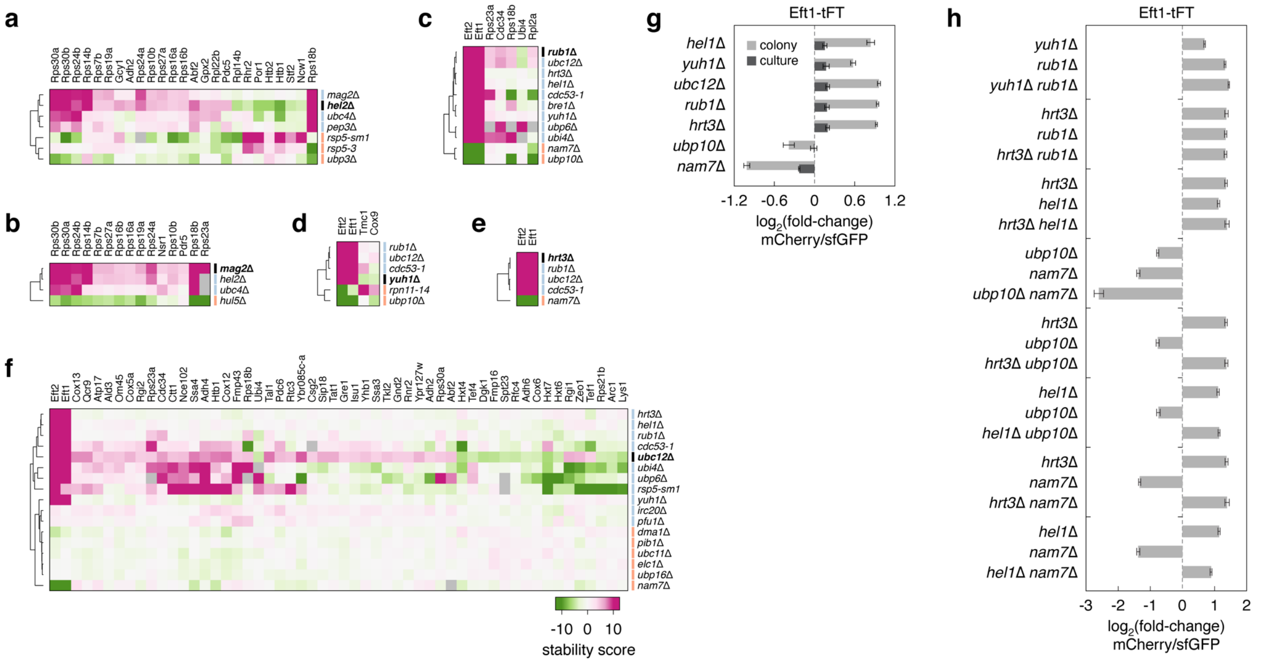

Figure S6. Protein turnover changes underlying correlations of proteome turnover profiles, related to Figure 4

a-f – Summary heatmaps of protein stability changes in the absence of the indicated components of the ubiquitin-proteasome system. Stability scores are color-coded from green (stability decrease) to magenta (stability increase). In each heatmap, only proteins with a significant change in stability in the mutant highlighted in bold (1% false discovery rate and absolute stability score > 4) are shown and their behavior in mutants with correlated phenotypes (blue – mutants with positive correlation of proteome turnover profile, orange – negative correlation) is included for comparison.

g, h – Differences in mCherry/sfGFP ratios between the indicated mutants and wild type strains expressing Eft1-tFT (mean ± s.d., $n = 4$). Comparison between measurements of log phase cultures by flow cytometry and plate reader measurements of colonies (**g**); plate reader measurements of colonies (**h**).

Figure S7

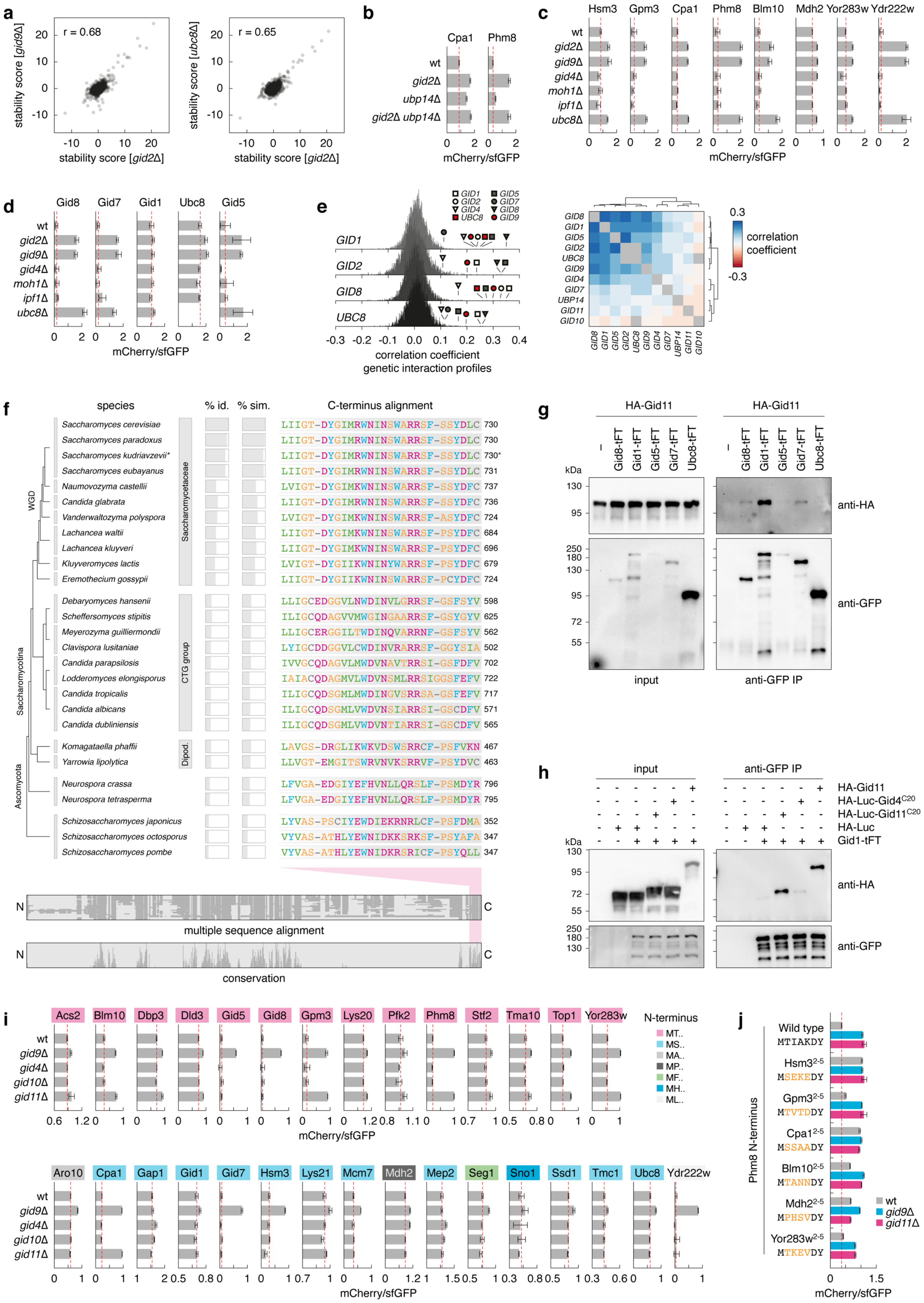

a
r = 0.68
r = 0.65
stability score [gid9Δ]
stability score [ubc8Δ]
stability score [gid2Δ]
b
Cpa1
Phm8
wt
gid2Δ
ubp14Δ
gid2Δ ubp14Δ
mCherry/sfGFP
c
Hsm3
Gpm3
Cpa1
Phm8
Blm10
Mdh2
Yor283w
Ydr222w
wt
gid2Δ
gid9Δ
gid4Δ
moh1Δ
ipf1Δ
ubc8Δ
mCherry/sfGFP
d
Gid8
Gid7
Gid1
Ubc8
Gid5
mCherry/sfGFP
e
GID1
GID2
GID8
UBC8
correlation coefficient
genetic interaction profiles
correlation coefficient
f
species
% id.
% sim.
C-terminus alignment
WGD
Saccharomycetaceae
CTG group
Dipod.
Saccharomycotina
Ascomycota
Saccharomyces cerevisiae
Saccharomyces paradoxus
Saccharomyces kudriavzevii*
Saccharomyces eubayanus
Naumovozyma castellii
Candida glabrata
Vanderwaltozyma polyspora
Lachancea waltii
Lachancea kluyveri
Kluyveromyces lactis
Eremothecium gossypii
Debaryomyces hansenii
Scheffersomyces stipitis
Meyerozyma guilliermondii
Clavispora lusitaniae
Candida parapsilosis
Lodderomyces elongisporus
Candida tropicalis
Candida albicans
Candida dubliniensis
Komagataella phaffii
Yarrowia lipolytica
Neurospora crassa
Neurospora tetrasperma
Schizosaccharomyces japonicus
Schizosaccharomyces octosporus
Schizosaccharomyces pombe
multiple sequence alignment
conservation
g
HA-Gid11
Gid8-tFT
Gid1-tFT
Gid5-tFT
Gid7-tFT
Ubc8-tFT
kDa
anti-HA
anti-GFP
input
anti-GFP IP
h
input
anti-GFP IP
HA-Gid11
HA-Luc-Gid4C20
HA-Luc-Gid11C20
HA-Luc
Gid1-tFT
anti-HA
anti-GFP
i
N-terminus
mCherry/sfGFP
j
Phm8 N-terminus
Wild type MTIAKDY
Hsm3 2-5 MSEKEDY
Gpm3 2-5 MTVTDDY
Cpa1 2-5 MSSAADY
Blm10 2-5 MTANNDY
Mdh2 2-5 MPHSVDY
Yor283w 2-5 MTKEVDY
wt
gid9Δ
gid11Δ
mCherry/sfGFP

Figure S7. Analysis of protein turnover by the GID complex, related to Figures 5 and 6

a – Comparison of changes in mCherry/sfGFP ratios (stability score) in strains lacking *GID2*, *GID9* or *UBC8* for the 3806 tested tFT queries.

b-d – mCherry/sfGFP ratios of colonies expressing tFT-tagged proteins and carrying the indicated mutations (mean ± s.d., *n* = 4).

e – Histograms of Pearson correlation coefficients (PCC) calculated between the genetic interaction profiles of the indicated GID genes and 75% of all yeast genes, obtained from a previously published genome-scale genetic interaction map (Costanzo et al., 2016) (left). Correlating GID genes (PCC > 0.1) are marked on the plots. Heatmap of Pearson correlation coefficients calculated between the genetic interaction profiles of all GID genes (right).

f – Gid11 conservation across yeasts. Left – putative Gid11 homologs in different yeast species (Table S7). The topology of the evolutionary tree was adapted from (Dujon, 2010). The branch lengths are arbitrary. WGD – whole-genome duplication, Dipod. – Dipodascaceae. Percentage of sequence identity and similarity (% id. and % sim., respectively) relative to *S. cerevisiae* Gid11 were calculated. *, the annotated Gid11 sequence of *Saccharomyces kudriavzevii* is 640 residues long, shorter than those of closely related species. This appears to be due to a premature stop codon caused by a single nucleotide deletion in codon 631. Here we reverted this single nucleotide deletion, which results in a protein 730 residues long. Bottom – multiple sequence alignment of putative Gid11 sequences and histogram of alignment conservation. Sequences were ordered following the evolutionary tree, from *S. cerevisiae* Gid11 (top) to *S. pombe* Gid11 (bottom). Gaps in the multiple sequence alignment are indicated in light gray. Right – C-terminal portion of the multiple sequence alignment, highlighting the C-terminal Φ[D/E]ΦX motif.

g – Co-immunoprecipitation analysis of the interaction between overexpressed HA-Gid11 and chromosomally tFT-tagged GID subunits or the ubiquitin-conjugating enzyme Ubc8.

h – Co-immunoprecipitation analysis of the interaction between endogenously tagged Gid1-tFT and HA-luciferase (HA-Luc), HA-Luc-Gid4^{C20} or HA-Luc-Gid11^{C20} (HA-Luc fused to the C-terminal 20 residues of Gid4 or Gid11, respectively). A strain with overexpressed HA-Gid11 was included for comparison.

i – mCherry/sfGFP ratios of colonies expressing tFT-tagged potential GID substrates (Fig. 5a) and lacking GID components (mean ± s.d., *n* = 3). Proteins are color-coded according to the identity of the residue after the initiator methionine. Note that for some tFT fusions the x-axis does not start at zero to clearly visualize the small but reproducible effects of *gid* mutants.

j – mCherry/sfGFP ratios of colonies expressing tFT-tagged Phm8 variants with different N-termini (mean ± s.d., *n* = 4). The sequence of each N-terminus and the potential GID substrate from which the first four residues after the initiator methionine were derived are indicated.
