# Supplementary material for: Timer-based proteomic profiling of the ubiquitin-proteasome system reveals a substrate receptor of the GID ubiquitin ligase
Source: Mol Cell. 2021 Jun 3;81(11):2460–2476.e11. doi: 10.1016/j.molcel.2021.04.018 (PMC8189435; doi:10.1016/j.molcel.2021.04.018)
Supplement: Document S2. Article plus supplemental information [file mmc9.pdf]

Molecular Cell

Resource

Timer-based proteomic profiling of the ubiquitin-proteasome system reveals a substrate receptor of the GID ubiquitin ligase

Graphical abstract

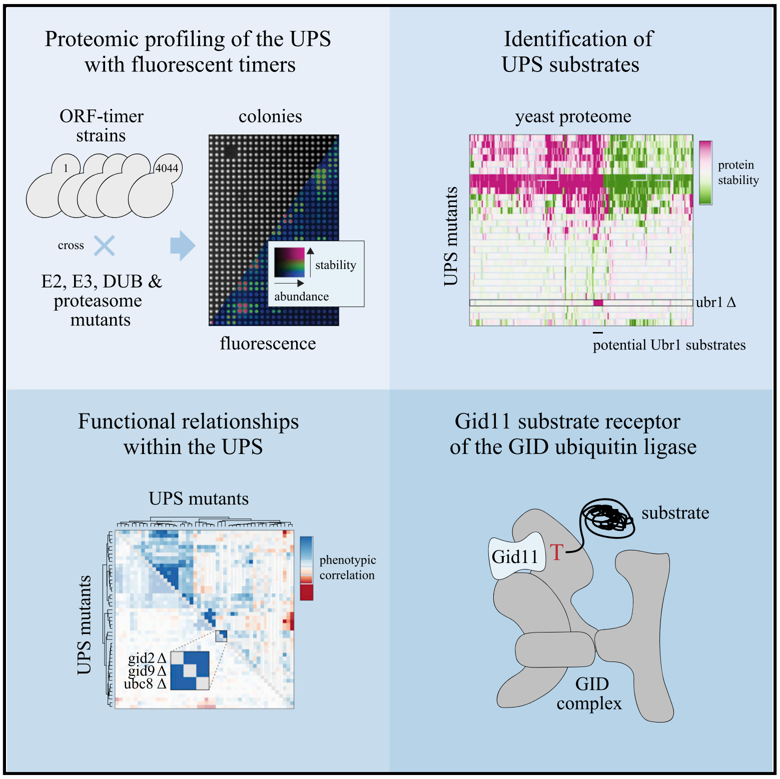

Highlights

- Global analysis of proteome turnover in budding yeast
- Proteome turnover profiles for most components of the ubiquitin-proteasome system
- Substrates and functions for Ubr1 and GID, two E3s of the N-degron pathways
- Identification of a GID receptor for substrates with a threonine N terminus

Authors

Ka-Yiu Edwin Kong, Bernd Fischer, Matthias Meurer, ..., Charles Boone, Michael Knop, Anton Khmelinskii

Correspondence

m.knop@zmbh.uni-heidelberg.de (M.K.), a.khmelinskii@imb-mainz.de (A.K.)

In brief

The ubiquitin-proteasome system (UPS) is key for selective protein degradation in eukaryotes. To understand its specificity, Kong et al. systematically analyze the effect of UPS mutants on yeast proteome turnover and explore the resulting resource of potential substrates and relationships between UPS components to gain insights into N-degron pathways.

Kong et al., 2021, Molecular Cell 81, 2460–2476
June 3, 2021 © 2021 The Author(s). Published by Elsevier Inc.
https://doi.org/10.1016/j.molcel.2021.04.018

CellPress
OPEN ACCESS

Molecular Cell

Resource

Timer-based proteomic profiling of the ubiquitin-proteasome system reveals a substrate receptor of the GID ubiquitin ligase

Ka-Yiu Edwin Kong,[1,9] Bernd Fischer,[2,9,10] Matthias Meurer,[3,9] Ilia Kats,[3,8] Zhaoyan Li,[1] Frank Rühle,[1] Joseph D. Barry,[4] Daniel Kirrmaier,[3,7] Veronika Chevyreva,[3] Bryan-Joseph San Luis,[5,6] Michael Costanzo,[5,6] Wolfgang Huber,[4] Brenda J. Andrews,[5,6] Charles Boone,[5,6] Michael Knop,[3,7,*] and Anton Khmelinskii[1,11,*]

[1]Institute of Molecular Biology (IMB), Mainz, Germany
[2]Computational Genome Biology, German Cancer Research Center (DKFZ), Heidelberg, Germany
[3]Center for Molecular Biology of Heidelberg University (ZMBH), DKFZ-ZMBH Alliance, Heidelberg, Germany
[4]Genome Biology Unit, European Molecular Biology Laboratory (EMBL), Heidelberg, Germany
[5]The Donnelly Centre for Cellular and Biomolecular Research, University of Toronto, Toronto, ON, Canada
[6]Department of Molecular Genetics, University of Toronto, Toronto, ON, Canada
[7]Cell Morphogenesis and Signal Transduction, German Cancer Research Center (DKFZ), DKFZ-ZMBH Alliance, Heidelberg, Germany
[8]Present address: Computational Genomics and System Genetics, German Cancer Research Center (DKFZ), Heidelberg, Germany
[9]These authors contributed equally
[10]Deceased
[11]Lead contact
*Correspondence: m.knop@zmbh.uni-heidelberg.de (M.K.), a.khmelinskii@imb-mainz.de (A.K.)
https://doi.org/10.1016/j.molcel.2021.04.018

SUMMARY

Selective protein degradation by the ubiquitin-proteasome system (UPS) is involved in all cellular processes. However, the substrates and specificity of most UPS components are not well understood. Here we systematically characterized the UPS in *Saccharomyces cerevisiae*. Using fluorescent timers, we determined how loss of individual UPS components affects yeast proteome turnover, detecting phenotypes for 76% of E2, E3, and deubiquitinating enzymes. We exploit this dataset to gain insights into N-degron pathways, which target proteins carrying N-terminal degradation signals. We implicate Ubr1, an E3 of the Arg/N-degron pathway, in targeting mitochondrial proteins processed by the mitochondrial inner membrane protease. Moreover, we identify Ylr149c/Gid11 as a substrate receptor of the glucose-induced degradation-deficient (GID) complex, an E3 of the Pro/N-degron pathway. Our results suggest that Gid11 recognizes proteins with N-terminal threonines, expanding the specificity of the GID complex. This resource of potential substrates and relationships between UPS components enables exploring functions of selective protein degradation.

INTRODUCTION

The functional state of a cell is ultimately defined by the state of its proteome; i.e., the abundance, localization, turnover, and mobility of all proteins and their organization in complexes and organelles. Proteome integrity is maintained by a complex proteostasis network that regulates protein synthesis, folding, transport, and degradation (Balch et al., 2008; Balchin et al., 2016; Wolff et al., 2014). Numerous protein quality control systems operate throughout the protein life cycle and contribute to proteome homeostasis by preventing, detecting, and removing abnormal proteins. In eukaryotes, the ubiquitin-proteasome system (UPS) plays a key role in selective protein degradation, where a cascade of ubiquitin-activating (E1), ubiquitin-conjugating (E2), and ubiquitin-protein ligase (E3) enzymes marks proteins with ubiquitin. The functional outcome of ubiquitination (e.g., proteasomal or lysosomal degradation, change in localization, or protein-protein interactions) depends on the type of modification (mono- versus polyubiquitination) and the type of polyubiquitin chains (Hershko and Ciechanover, 1998; Kleiger and Mayor, 2014; Zheng and Shabek, 2017). Deubiquitinating enzymes (DUBs), which remove ubiquitin from target proteins and replenish the pool of free ubiquitin, are involved at various stages of the targeting and degradation processes (Mevissen and Komander, 2017; Oh et al., 2018).

Specificity in the UPS is provided by E3s. The human genome encodes more than 600 E3s, and even a simple organism, such as the budding yeast *S. cerevisiae*, has ~100 E3s, which are thought to recognize and ubiquitinate distinct sets of proteins (Finley et al., 2012; Pickart, 2001; Zheng and Shabek, 2017). Substrate recognition appears to involve E3 binding, directly or via cofactors, to short linear motifs in the substrate, known as degradation signals or degrons if ubiquitination leads to degradation (Ella et al., 2019; Ravid and Hochstrasser, 2008; Zheng and Shabek, 2017). A prominent class of degrons is located at

 © 2021 The Author(s). Published by Elsevier Inc.
This is an open access article under the CC BY license (http://creativecommons.org/licenses/by/4.0/).

CellPress
OPEN ACCESS

protein N termini. These N-degrons, defined by the first few N-terminal residues, are recognized by different E3s called N-recognins (Varshavsky, 2011, 2019).

In the Arg/N-degron pathway, the budding yeast E3 Ubr1 recognizes and targets for proteasomal degradation proteins with positively charged (R, K, H) or bulky hydrophobic (W, L, F, Y, I) N-terminal residues (Bachmair and Varshavsky, 1989; Bachmair et al., 1986). In addition, proteins with N-terminal N or Q residues can be processed by the N-terminal amidase Nta1, yielding N termini with D or E residues, followed by their arginylation by the N-terminal arginyl-transferase Ate1, producing N termini that start with an arginine and are thus targets of Ubr1 (Baker and Varshavsky, 1995; Balzi et al., 1990). Ubr1 can also recognize N termini where the unacetylated initiator methionine is followed by a bulky hydrophobic residue (Kim et al., 2014). Despite its well-established specificity, few substrates are known for the Arg/N-degron pathway because they are usually generated through endoproteolysis and, thus, difficult to identify (Varshavsky, 2011).

The GID (glucose-induced degradation-deficient) complex is a multisubunit E3 conserved in eukaryotes (Francis et al., 2013). In yeast, it functions as the N-recognin of the Pro/N-degron pathway, where it targets the gluconeogenic enzymes fructose-1,6-bisphosphatase Fbp1, malate dehydrogenase Mdh2, phosphoenolpyruvate carboxykinase Pck1, and isocitrate lyase Icl1 for proteasomal degradation when switching from ethanol to glucose as a carbon source (Chen et al., 2017; Hämmerle et al., 1998; Regelmann et al., 2003; Santt et al., 2008). These substrates carry N-degrons with a proline as the N-terminal or second residue for recognition by the receptor subunit Gid4 (Chen et al., 2017; Hämmerle et al., 1998). Gid4 expression is induced during the switch of carbon source, ensuring timely inactivation of gluconeogenesis (Menssen et al., 2018; Santt et al., 2008). The GID E3 likely has additional functions because the core complex is present under various conditions, and a second substrate receptor, Gid10, is induced by starvation or osmotic stress (Chen et al., 2017; Melnykov et al., 2019; Qiao et al., 2020).

Because of its major roles in recycling of unnecessary or abnormal proteins, the UPS is involved in essentially all cellular processes (Hershko and Ciechanover, 1998; Kleiger and Mayor, 2014; Zheng and Shabek, 2017). Failure in selective protein degradation and quality control are associated with various diseases, including cancer and neurodegenerative disorders. Moreover, proteome homeostasis declines with age (Balch et al., 2008; Balchin et al., 2016; Labbadia and Morimoto, 2015). Despite the central role of the UPS in cell physiology, the functions of many UPS components are unclear, and the substrate specificities of E3s and DUBs are not well defined.

Here we used budding yeast to systematically assess the role of UPS components in proteome homeostasis. Besides the ~100 E3s and accessory subunits, the yeast UPS consists of a single E1, 11 E2s, 21 DUBs, the proteasome, and regulatory factors (Finley et al., 2012). We examined how inactivation of UPS components affects proteome abundance and turnover using tandem fluorescent protein timers (tFTs). A tFT is a tag composed of two fluorescent proteins with different kinetics of fluorophore maturation, such as mCherry and superfolder GFP (sfGFP) (Figure 1a). The mCherry/sfGFP ratio of fluorescence intensities is a measure of protein turnover in the steady state, increasing as a function of stability of the tFT-tagged protein (Figure 1A; Khmelinskii et al., 2012), whereas the sfGFP signal is a measure of protein abundance. Using a proteome-wide library of strains expressing tFT-tagged proteins (Khmelinskii et al., 2014), we profiled the yeast proteome in 132 mutants, including most E2, E3, and DUB enzymes. We exploit the resulting dataset to define functions for various UPS components and to gain insight into N-degron pathways.

RESULTS

Yeast proteome turnover

We used the tFT library to characterize *S. cerevisiae* proteome abundance and turnover. The library consists of 4,044 strains, each with one open reading frame (ORF) tagged chromosomally with the mCherry-sfGFP timer (Khmelinskii et al., 2014; Figure 1A). We grew the library as an ordered array of colonies and measured their mCherry and sfGFP intensities (STAR Methods). sfGFP intensities provided reproducible estimates of protein abundance (Figures S1A and S1B; Table S1; Pearson correlation coefficient [r] = 0.99 between 2 library replicates), in line with independent measurements (Ghaemmaghami et al., 2003; de Godoy et al., 2008; Newman et al., 2006; Figures S1C–S1E). mCherry/sfGFP ratios provided reproducible estimates of protein turnover (Figure S1F; Table S1), although the associated error was higher because of combined uncertainties in measuring mCherry and sfGFP intensities (r = 0.89 between 2 replicates). The distribution of mCherry/sfGFP ratios was skewed toward unstable proteins (Figures 1B and 1C), suggesting that degradation is frequently used to tune protein abundance. This is consistent with observations in yeast and other organisms (Belle et al., 2006; Boisvert et al., 2012; Cambridge et al., 2011; Kristensen et al., 2013; Schwanhäusser et al., 2011), although we cannot exclude a contribution of the nonlinear relationship between mCherry/sfGFP ratios and protein half-life to this trend (Figure S1G). The correlation between mCherry/sfGFP ratios and protein half-lives determined with cycloheximide chases of strains expressing proteins fused to a tandem affinity purification (TAP) tag (Belle et al., 2006) or pulse SILAC (stable isotope labeling by amino acids in cell culture) mass spectrometry (Christiano et al., 2014) was low (Figures S1H and S1I; r = 0.26 or 0.32). The correlation between the cycloheximide chase and pulse SILAC datasets was even lower (Figure S1J; r = 0.18). Multiple factors contribute to these discrepancies, including different growth conditions used in each study, the effect of cycloheximide on cell physiology, the potential effect of bulky tFT and TAP tags on protein turnover, and the inherent bias of mass spectrometry against low-abundance proteins.

Nevertheless, the distribution of mCherry/sfGFP ratios revealed global features of proteome turnover. First, mCherry/sfGFP ratios correlated with protein abundance so that low-abundance proteins were less stable (Figure 1C). Second, protein stability varied with protein localization and function. For instance, DNA binding proteins and proteins involved in cell cycle progression or localized to the mitotic spindle exhibited faster turnover, whereas abundant components of

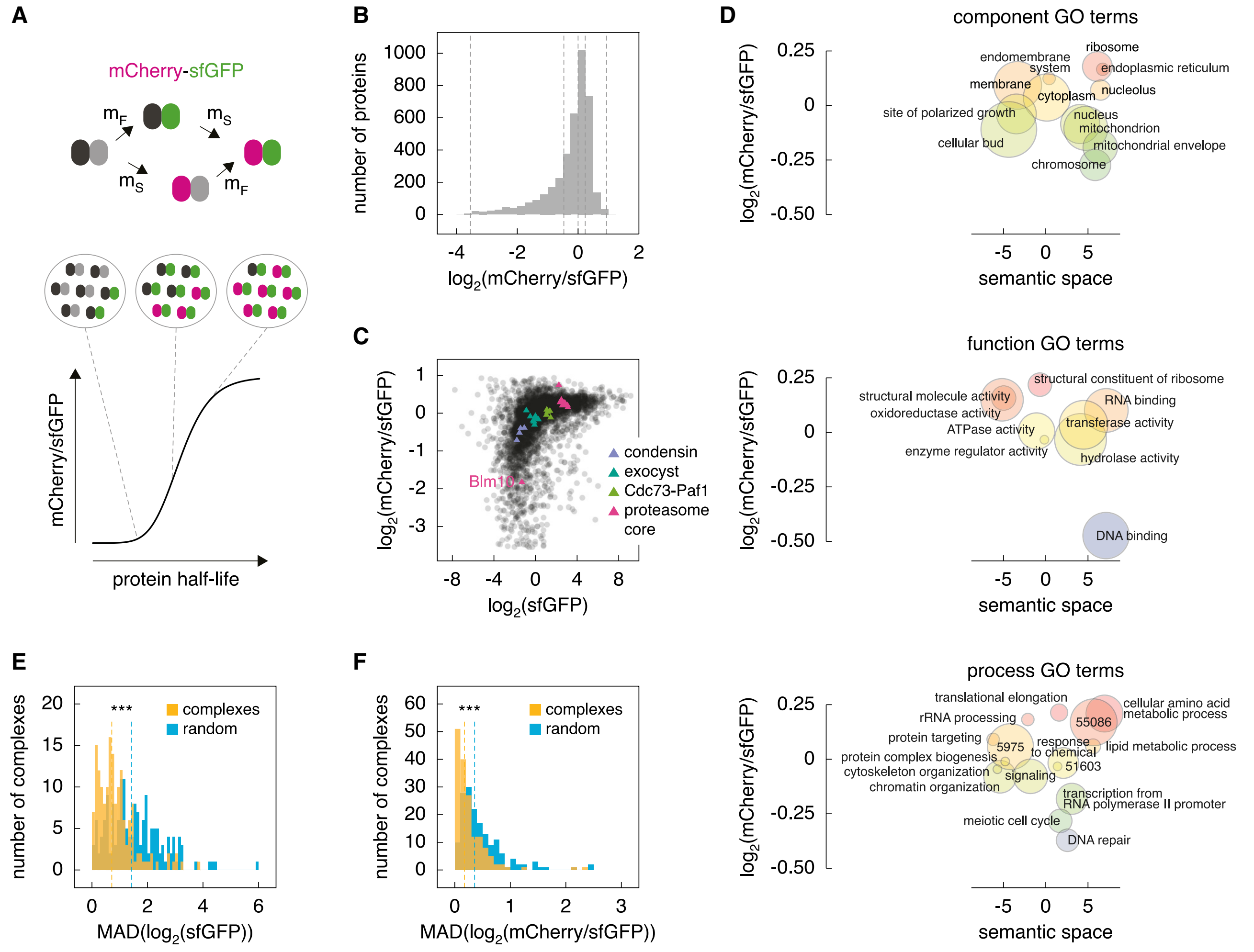

Figure 1. Turnover of the yeast proteome

(A) Cartoon of the mCherry-sfGFP timer (top). Because of different maturation kinetics of mCherry (slow, m_S) and sfGFP (fast, m_F), the mCherry/sfGFP ratio reports the stability of timer-tagged proteins in the steady state (bottom).

(B) Median-centered distribution of mCherry/sfGFP ratios in the tFT library, representing protein stability in the yeast proteome. Shown are fluorescence measurements of colonies, median of 2 biological replicates, each with 4 technical replicates per protein. Dashed lines, quantiles used in downstream analyses (Figures S1M and S1N).

(C) Relationship between sfGFP intensities (protein abundance) and mCherry/sfGFP ratios in the tFT library. Example protein complexes are highlighted.

(D) Median mCherry/sfGFP ratios of proteins in the tFT library mapped to Gene Ontology (GO) terms. GO term 5975, carbohydrate metabolic process; 51603, proteolysis involved in cellular protein catabolic process; 55086, nucleobase-containing small molecule metabolic process. Similar GO terms are closer in semantic space (Supek et al., 2011).

(E and F) Distributions of median absolute deviations (MADs) of sfGFP intensities (E) or mCherry/sfGFP ratios (F) for complexes in the tFT library. Random samples of the proteome, drawn in sets of N (where N is the number of complex subunits), are shown for comparison (n = 100 random draws). Dashed lines, medians of the distributions. ***$p < 0.001$ in a Wilcoxon rank-sum test.

See also Figure S1 and Table S1.

housekeeping machinery, such as ribosomes or metabolic enzymes, were stable (Figures 1D and S1K), consistent with previous observations (Belle et al., 2006; Boisvert et al., 2012; Cambridge et al., 2011; Kristensen et al., 2013; Schwanhäusser et al., 2011). Yet, mCherry/sfGFP ratios should be compared with caution between subcellular compartments because the tFT readout can be affected by the intracellular environment (Khmelinskii and Knop, 2014). For example, mCherry/sfGFP ratios of secretory proteins varied with location of their C termini (Kim et al., 2006; Figure S1L). Third, protein abundance and stability within protein complexes were more similar than expected at random (Figures 1E and 1F), highlighting that complex subunits are co-regulated and that assembly into a complex can stabilize individual subunits, equalizing their turnover (Dephoure et al., 2014; Li et al., 2014; McShane et al., 2016; Taggart and Li, 2018). Interestingly, some subunits exhibited clearly distinct and faster turnover (e.g., the proteasome activator Blm10 was less stable compared with other proteasomal subunits;

Figure 1C), suggesting a regulatory role of protein turnover in complex assembly or function.

Finally, we searched for sequence features correlated with protein half-life. Unstable proteins were enriched in cysteine, serine, and asparagine but depleted of alanine, glycine, and valine residues (Figure S1M). These trends are reminiscent of those observed by Belle et al. (2006) and were present even after excluding disordered regions from the analysis (Figure S1N). However, their significance remains unclear. Protein stability was negatively correlated with the presence and number of long disordered regions (Figure S1O), which can increase the efficiency of proteasomal degradation by serving as initiation sites (van der Lee et al., 2014). These results demonstrate that abundance and turnover of the yeast proteome can be analyzed with the tFT library.

Functional profiling of the UPS

To gain insights into UPS functions, we examined how impairing individual UPS components affects yeast proteome abundance and turnover. We crossed the tFT library with an array of strains carrying knockout alleles of non-essential UPS components (Winzeler et al., 1999) or temperature-sensitive alleles of essential UPS factors (Li et al., 2011; Figure 2A; UPS array). The UPS array encompassed almost all known E2s, E3s, and DUBs, including substrate adaptors of E3s such as Rsp5 and SCF (Skp, Cullin, F-box-containing complex), several proteasomal subunits, and the autophagy factors Atg8 and Atg12 (Table S2). For large complexes, mutants of only one or a few core subunits were included in the array. We performed each cross in 4 technical replicates, grew the resulting haploids carrying tFT and UPS mutant alleles as ordered colony arrays, and measured their mCherry and sfGFP fluorescence. In total, we performed ~2.5 million crosses, corresponding to more than 620,000 mutant-tFT pairs.

After correcting the data for crossing efficiency and plate and batch effects, we determined the effect of each of 132 UPS mutants on the abundance (sfGFP intensity) and stability (mCherry/sfGFP ratio) of each tFT-tagged protein (STAR Methods). Our screening procedure was robust, as shown by two sets of control screens. First, two tFT strains (*UBI4-tFT*, which should be stabilized in the absence of Ubr1, as detailed below, and the randomly chosen *YJR096W-tFT*) were screened in every batch a total of 24 times with reproducible results (Figure S2A). Second, a random set of 96 tFT strains was screened twice with good correlation between the replicates (Figures S2B and S2C; $r > 0.7$).

Overall, we detected significant changes in protein abundance or stability in ~4.5% of mutant-tFT pairs (3.4% for abundance, 1.9% for stability; Figure 2B; Table S3). This frequency is similar to that of protein-protein interactions (Yu et al., 2008) or genetic interactions (Costanzo et al., 2016). Changes in protein abundance generally correlated with changes in stability, where stabilization was more frequently accompanied by an increase rather than a decrease in abundance, and vice versa (Figure 2B). Instances of anticorrelated changes in protein abundance and stability could reflect more complex regulation or be caused by the higher error associated with estimating changes in mCherry/sfGFP ratios. Globally, proteins that were stabilized in the screen were less stable in wild-type cells (Figure S2D), further arguing for the validity of our approach. On average, the abundance or stability of a given protein were affected in only ~1–2 mutants (Figures 2C, S2E, and S2F). Notably, in total, only ~56% of proteins were affected in at least one of the tested mutants (33% and 52% with changes in stability and abundance, respectively), which could be explained by condition-specific proteome turnover and redundancies in the UPS. Supporting this notion, mutants of 6 of 16 adaptors of the Rsp5 E3 had no effect on proteome turnover (Figure S2G), consistent with their high redundancy and conditional functions (Nikko and Pelham, 2009). Overall, the number of proteins affected by a given mutant varied greatly (Figures 2D and S2G–S2I). Cumulatively more proteins were affected by mutants of E3s compared with DUBs or E2 enzymes (Figures 2E and S2J), suggesting higher levels of redundancy between E2 enzymes or DUBs compared with E3s. It is also possible that some ubiquitination events are not accessible to DUBs and, thus, not affected in any DUB mutant.

Hereafter, we refer to mutant-tFT pairs where the stability or abundance of the tFT-tagged protein is affected significantly as turnover or abundance interactions, respectively. To understand the nature of these interactions, we compared them with various datasets: protein-protein interactions, collected from high-throughput and low-throughput experiments in the BioGRID (biological general repository for interaction datasets) (Oughtred et al., 2019); a dataset of genome-wide changes in mRNA expression and correlations of expression profiles determined for deletion mutants of ~25% of all protein-coding genes, including UPS components (Kemmeren et al., 2014); and a genome-wide dataset of genetic interactions, measured by comparing the fitness of single and double mutants and corresponding correlations of genetic interaction profiles determined for ~90% of all yeast genes (Costanzo et al., 2016). Turnover and abundance interactions were supported by protein-protein interactions between UPS factors and the affected proteins (Figures 2F and S2K). This is expected when substrates of selective protein degradation are affected in mutants of the corresponding targeting machinery. Indeed, we observed stabilization of well-defined substrates of various E3s in the screen (Figure S3A). Turnover and abundance interactions were also supported by changes in gene expression, and, interestingly, by genetic interactions and correlations of genetic interaction profiles (Figures 2F and S2K), which are an indication of functional similarity (Costanzo et al., 2016). This suggests that a fraction of interactions occurred between functionally related factors. Supporting this notion, we observed a significant degree of self-regulation in the UPS; the frequency of turnover interactions within the UPS was 3.2% compared with 1.9% for the whole proteome (Figures 2B, S3B, and S3C).

Next, we used turnover interactions to explore functions of various UPS components in more detail.

Ltn1. Ltn1 is an E3 that functions in ribosome-associated protein quality control (RQC). It is involved in targeting for proteasomal degradation stalled nascent polypeptides resulting from translation of mRNAs lacking a stop codon, which leads to translation of poly(A) tails into polylysine tracts, or from translation of mRNAs encoding a strong polybasic tract

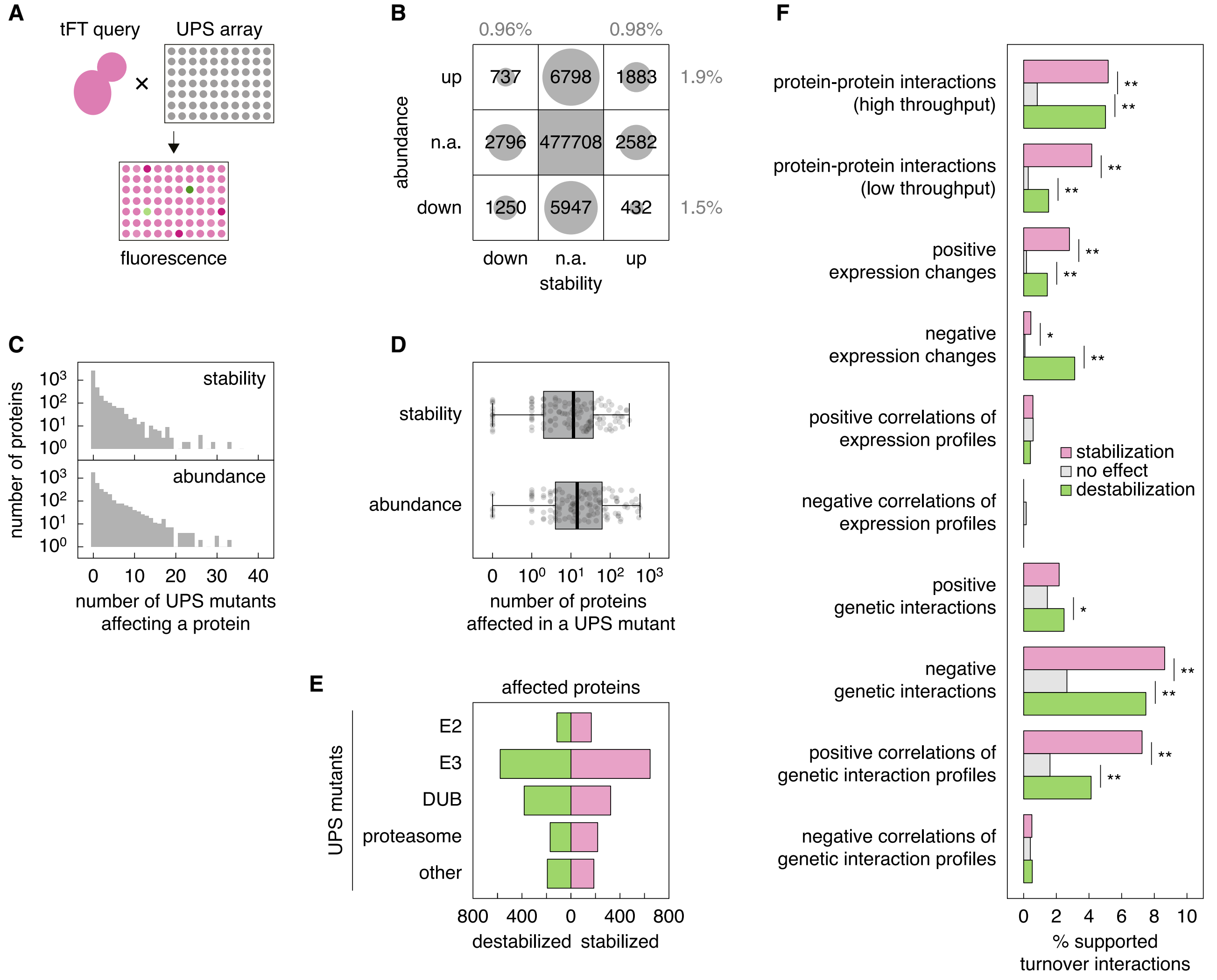

Figure 2. Influence of UPS components on proteome abundance and stability

(A) Cartoon of screens to profile the yeast UPS. Each strain in the tFT library (tFT query) was crossed with an array of mutants in UPS components (UPS array), followed by mCherry and sfGFP fluorescence measurements of colonies.

(B) Summary of phenotypic outcomes (changes of protein abundance and stability; n.a., not affected) across all tested mutant-tFT pairs at 1% FDR (false discovery rate). The percentage of mutant-tFT pairs with each phenotype is indicated.

(C) Number of mutants affecting protein stability or abundance for the 3,806 tested tFT queries. Only significant interactions (1% FDR, absolute stability or abundance score > 4) were considered (C–E).

(D) Number of proteins affected in terms of stability or abundance in 132 mutants in the UPS array. Centerlines mark the medians, box limits indicate the 25th and 75th percentiles, and whiskers extend to minimum and maximum values.

(E) Number of proteins destabilized or stabilized in UPS mutants grouped by function (Table S2).

(F) Overlap between turnover interactions, grouped according to change in protein stability at 1% FDR and external datasets. *$p < 0.05$ and **$p < 0.01$ in a Fisher's exact test.

See also Figures S2–S4 and Tables S2 and S3.

(Bengtson and Joazeiro, 2010; Brandman et al., 2012; Joazeiro, 2019). In these cases, electrostatic interactions between polybasic tracts and the ribosome exit tunnel are thought to cause ribosome stalling (Lu and Deutsch, 2008). Interestingly, Ltn1 appears to control the levels of Rqc1, another RQC factor. This control depends on a polybasic stretch located in the N-terminal portion of Rqc1, raising the possibility that Rqc1 is an Ltn1 substrate (Brandman et al., 2012). Indeed, we observed strong stabilization of Rqc1 in the absence of Ltn1 (Figure S4A). Surprisingly, of 62 yeast proteins that contain a strong polybasic stretch (Brandman et al., 2012), only Rqc1 and Nop12 were stabilized in *ltn1Δ* cells (Figure S4A). Therefore, polybasic stretches in endogenous proteins do not commonly lead to Ltn1-dependent degradation, at least under our experimental conditions, in agreement with a recent report (Barros et al., 2021).

Ubc13-Mms2. It is worth noting that a given UPS mutant, on average, stabilized and destabilized a similar number of proteins (Figure S2G), indicating that a significant fraction of turnover and abundance interactions is likely explained by indirect effects or adaptation of mutant strains to long-term loss of UPS factors. Nevertheless, gene set enrichment analysis suggested that, for ~48% (63 of 132) of UPS mutants, the phenotypes were specific because the affected proteins were associated with defined signatures (Figure S4B). For instance, the Asi, Hrd1, and Tul1 E3s are involved in turnover of proteins in the endomembrane system (Foresti et al., 2014; Khmelinskii et al., 2014; Reggiori and Pelham, 2002; Ruggiano et al., 2014; Yang et al., 2018). Accordingly, proteins affected in the corresponding mutants were enriched in transmembrane domains and localized to the endoplasmic reticulum or vacuole. Interestingly, we observed a similar trend for Ubc13 (Figure S4B). Together with Mms2, Ubc13 forms a heteromeric E2 enzyme involved in replication of damaged DNA (Hofmann and Pickart, 1999). However, a second role of Ubc13-Mms2 in sorting of membrane proteins has been reported recently (Renz et al., 2020). Indeed, 13 of 25 proteins affected in *ubc13*Δ cells (an *mms2*Δ mutant was not included in the screen) had a transmembrane domain compared with 17% in the tFT library (Figure S4C), indicating that sorting of membrane proteins is a key function of Ubc13-Mms2 under unstressed conditions.

Ubr1. The E3 Ubr1 can target for proteasomal degradation proteins carrying N-degrons (Varshavsky, 2011). In the Arg/N-degron pathway, such N-degrons are typically exposed through endoproteolytic cleavage and, depending on the identity of the new N-terminal residue, recognized by Ubr1 directly or upon modification by the N-terminal amidase Nta1 and/or the N-terminal arginyl-transferase Ate1 (Varshavsky, 2011; Figure 3A). Two substrates of the Arg/N-degron pathways were among the proteins stabilized in the absence of Ubr1 or the cognate E2 enzyme Rad6: the cohesin subunit Mcd1, which is subject to proteolytic cleavage by the protease Esp1/separase, exposing a destabilizing arginine residue at the N terminus (Rao et al., 2001), and the N-tFT protein generated in the *UBI4-tFT* strain (Figure 3B). Ubi4 encodes a polyubiquitin precursor that is efficiently processed into free ubiquitin by DUBs (Özkaynak et al., 1984). In addition to free ubiquitin, processing of tFT-tagged Ubi4 results in a free tFT moiety with an N-terminal asparagine residue (N-tFT), which is then modified by Nta1 and Ate1 before Ubr1-mediated degradation. Accordingly, the mCherry/sfGFP ratio of the *UBI4-tFT* strain was increased upon deletion of *NTA1*, *ATE1*, or *UBR1* (Figure 3C). Interestingly, the same phenotype was observed in the autophagy mutants *atg8*Δ and *atg12*Δ (Figure S2A), although the reasons for this stabilization are unclear.

Multiple potential Ubr1 substrates were also stabilized in the *nta1*Δ or *ate1*Δ mutants (Figures 3B and 3C), suggesting that these proteins carry N-degrons exposed via proteolytic processing. We thus used immunoblotting to search for potential proteolytic fragments in strains expressing C-terminally TAP-tagged proteins. During logarithmic growth, we observed accumulation of an Mcr1 fragment in *ubr1*Δ cells. This phenotype was exacerbated upon glucose starvation (Figure 3D), a condition that is closer to cells in a colony (Cáp et al., 2012). Mcr1 is a mitochondrial NADH-cytochrome b5 reductase that exists in two isoforms: a full-length, 34-kDa protein inserted into the outer mitochondrial membrane (Mcr1(34)) and a shorter, 32-kDa isoform located in the intermembrane space (Mcr1(32)) (Hahne et al., 1994). This shorter isoform results from Mcr1 proteolysis by the mitochondrial inner membrane peptidase Imp1, which exposes a glutamate residue at the Mcr1(32) N terminus (Hahne et al., 1994), making it a potential Ate1 substrate (Figure 3A). Accordingly, Mcr1-tFT was stabilized in *ate1*Δ cells, and Mcr1(32) accumulated in the absence of Ate1 or upon proteasome inhibition (Figures 3C, 3D, and S4D).

We asked whether the bulky tFT and TAP tags might interfere with mitochondrial import of Mcr1 and explain these phenotypes. Mcr1(32) fused a small 3× hemagglutinin (HA) tag also accumulated in *ubr1*Δ cells, as evidenced by a higher Mcr1(32)/Mcr1(34) ratio in the *ubr1*Δ mutant compared with the wild-type, although to a substantially reduced extent relative to the TAP-tagged variant (Figures 3D and 3E). Inhibiting Mcr1-3×HA import into mitochondria (thus preventing its processing by Imp1) by deletion of the Tom7 and Tim11 subunits of the translocases of the outer and inner membrane complexes, respectively, prevented Mcr1(32) accumulation in the absence of Ubr1 (Figure 3E). These results suggest that incomplete mitochondrial import of Mcr1 (e.g., caused by a bulky tag) leads to release of N-terminally processed Mcr1(32) into the cytosol, where Ubr1 and Ate1 localize (Huh et al., 2003; Varshavsky, 2011), followed by its Ate1/Ubr1-dependent degradation. Although Mcr1(32) accumulation can be modulated by the tag, we cannot formally exclude that altered Imp1 activity in Arg/N-degron mutants contributes to this phenotype. Nevertheless, considering the preference of Imp1 for aspartate and glutamate residues at the P'1 position (Luo et al., 2006), which becomes the new N terminus upon proteolysis, it seems likely that Ubr1 plays a more general role in quality control of mitochondrial protein import (Bragoszewski et al., 2015). An analogous pathway appears to operate in human cells, as exemplified by regulation of PINK1 (PTEN-induced kinase 1) in mitophagy. Whereas PINK1 accumulates in the outer membrane of damaged mitochondria and promotes mitophagy, under normal conditions, PINK1 is processed by the mitochondrial inner membrane rhomboid protease PARL (presenilin associated rhomboid like) and released into the cytosol, where it is targeted for degradation by the Arg/N-degron pathway (Greene et al., 2012; Harper et al., 2018; Jin et al., 2010; Meissner et al., 2011; Yamano and Youle, 2013). It will be interesting to examine whether the Arg/N-degron pathway plays a broader role in shaping the human mitochondrial proteome. This analysis demonstrates how protein turnover and abundance interactions can be used to identify new functions of selective protein degradation machinery.

Correlations of proteome turnover profiles

Next we explored our dataset to gain insights into functional relationships between UPS components. We calculated correlations of proteome turnover profiles between pairs of UPS mutants, adjusted for the number of affected proteins (Figures 4A and S5A–S5C; Table S4; STAR Methods). Up to 30% of positive

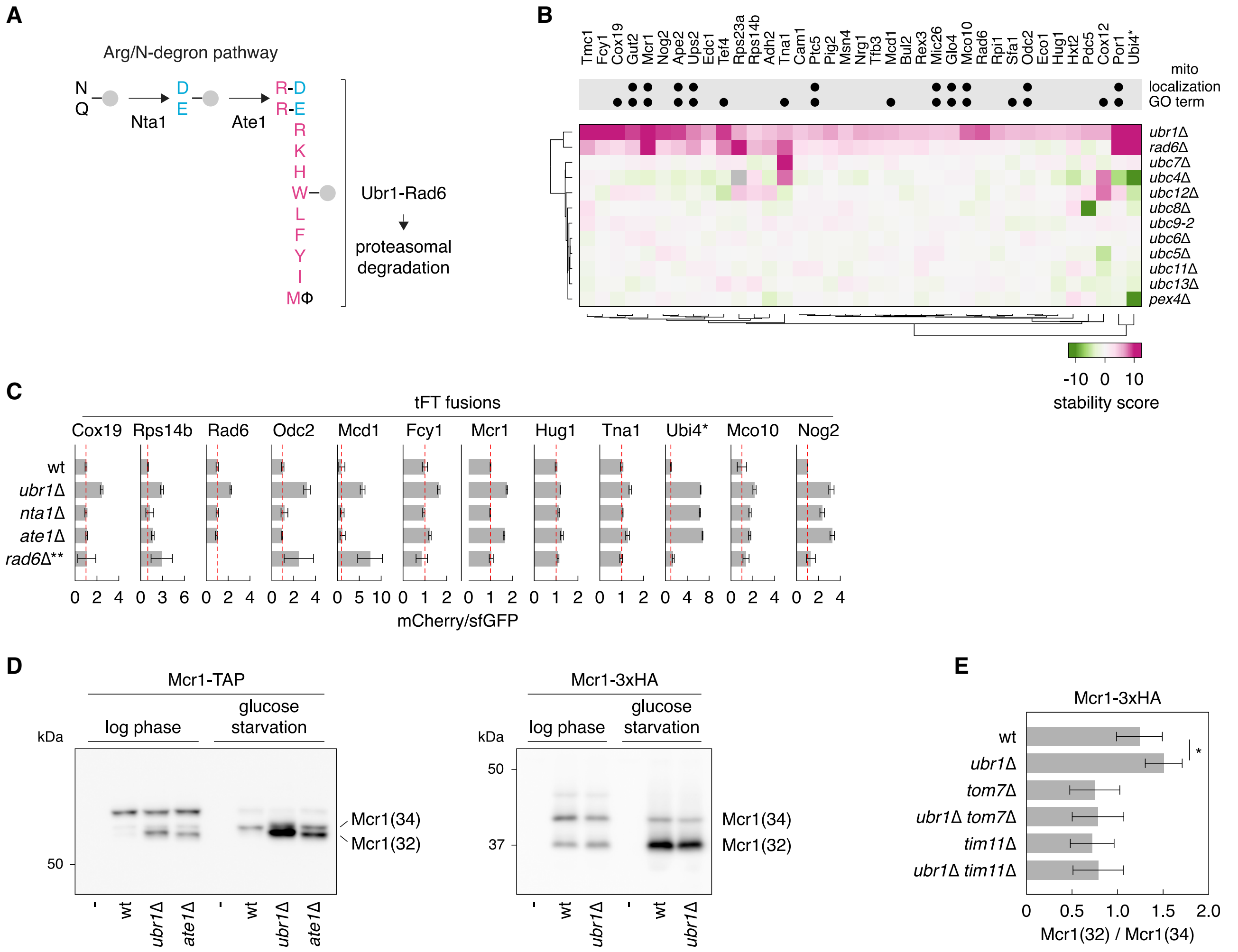

Figure 3. Ubr1-dependent protein turnover

(A) Scheme of the Arg/N-degron pathway, which targets proteins with the indicated N-terminal residues for degradation. Φ, large hydrophobic residues (W, L, F, Y, I).

(B) Heatmap of protein stability changes in the absence of Ubr1 (screens in Figure 2). Changes in mCherry/sfGFP ratios are color coded from green (decrease) to magenta (increase). Only proteins stabilized in the *ubr1*Δ mutant (1% FDR, stability score >4) are shown; their behavior in E2 mutants is included for comparison. Proteins localized to mitochondria based on GFP tagging (Huh et al., 2003) or mapped to the GO term mitochondrion are marked. *, Ubi4-tFT is not stabilized in the *ubr1*Δ mutant. Processing of the Ubi4-tFT fusion by DUBs releases free tFT with an N-terminal asparagine, which is the substrate of the Arg/N-degron pathway (B and C).

(C) mCherry/sfGFP ratios of colonies expressing tFT fusions and lacking components of the Arg/N-degron pathway (mean ± SD, n = 4). Hereafter, red dashed lines mark mCherry/sfGFP ratios in the wild type (WT). **, protein stability measurements in the *rad6*Δ mutant are confounded by its fitness defect; this effect is partially corrected for in the screen (B).

(D) Immunoblots of strains expressing Mcr1-TAP (left) or Mcr1-3xHA (right). Samples were collected from log-phase cultures or after 48 h of growth in low-glucose medium (glucose starvation).

(E) Quantification of Mcr1(32) and Mcr1(34) relative abundance by immunoblotting of strains expressing Mcr1-3xHA (mean ± SD, n = 2 biological replicates each with 3 technical replicates). *p < 0.04 in a one-sided unpaired t test.

See also Figure S4.

correlations of proteome turnover profiles were supported by protein-protein interactions (Oughtred et al., 2019) between UPS components, genetic interactions, and correlations of genetic interaction profiles (Costanzo et al., 2016; Figure 4B), indicating that UPS components with similar proteome turnover profiles are likely to interact physically or to be involved in the same process. Correlations of proteome turnover profiles provided stronger evidence of functional similarity compared with correlations of genetic interactions profiles because proteome turnover is likely a more informative phenotype to dissect functions of UPS components compared with fitness-based genetic interactions (Costanzo et al., 2016). This is exemplified by correlations between mutants of the Asi E3 and the Ubc7 E2 enzyme, which participate in protein quality control at the inner nuclear

CellPress
OPEN ACCESS

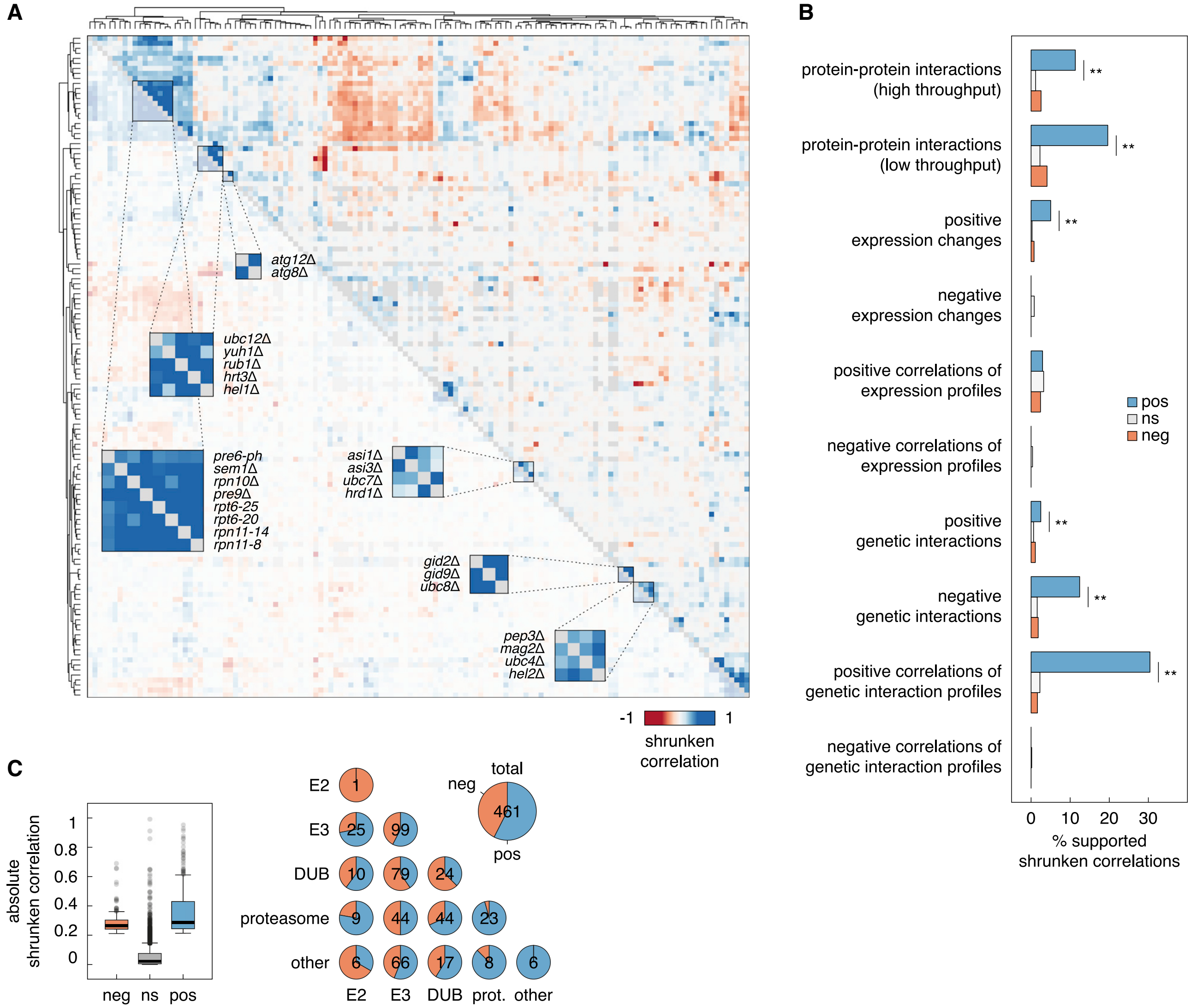

Figure 4. Correlations of proteome turnover profiles

(A) Heatmap of correlations of proteome turnover profiles for all tested mutants in the UPS array (screens in Figure 2). For each pair of mutants, a shrunken correlation was calculated based on the set of proteins with altered stability (1% FDR, absolute stability score >4) in at least one of the mutants. Select clusters of correlating mutants are highlighted.

(B) Overlap between shrunken correlations of proteome turnover profiles and external datasets. Correlations of proteome turnover profiles from (A) were grouped according to significance (1% FDR) and sign (pos, positive; ns, not significant; neg, negative). **p < 0.01 in a Fisher's exact test.

(C) Magnitude of shrunken correlations of proteome turnover profiles grouped according to significance and sign as in (B) (left) and number of significant correlations between UPS mutants grouped by function (Table S2) (right).

See also Figures S5 and S6 and Table S4.

membrane (Foresti et al., 2014; Khmelinskii et al., 2014; Figure S5D). We also expected to observe negative correlations of proteome turnover profiles because of the existence of opposing activities in the UPS, for instance, E3s and DUBs. Indeed, 43% of significant correlations in our dataset were negative, including 47 of 79 significant correlations between E3s and DUBs and 14 of 44 significant correlations between DUBs and the proteasome (Figure 4C; Table S4). Factors with anticorrelated phenotypes are less likely to be in one complex because negative correlations of proteome turnover profiles were not supported by protein-protein interactions (Figure 4B). These anticorrelations appeared to not be captured by correlations of fitness-based genetic interaction profiles, possibly because of the less specific phenotype or because of reduced sensitivity of the fitness assay toward positive genetic interactions (Baryshnikova et al., 2010a).

Based on this analysis, we conclude that correlations of proteome turnover profiles are a measure of functional similarity that could be used to identify complexes or pathways in the UPS. Supporting this notion, mutants of proteasomal components (Pre6, Sem1, Rpn10, Pre9, Rpt6, and Rpn11) exhibited similar

proteome turnover profiles and clustered together (Figure 4A). The same behavior was observed for the two mutants in autophagy genes, *atg8*Δ and *atg12*Δ; for mutants lacking subunits of the GID complex, Gid2 and Gid9; or for mutants in Asi and Hrd1 E3s, which have overlapping roles in protein abundance and quality control at the inner nuclear membrane and the endoplasmic reticulum (Foresti et al., 2014; Khmelinskii et al., 2014; Ruggiano et al., 2014; Figure 4A). Most correlations between DUBs and the proteasome were positive because of two DUBs, Rpn11 and Ubp6 (Figure 4C; Table S4), which are involved in recycling ubiquitin from ubiquitin-protein conjugates at the proteasome (Hanna et al., 2006; Leggett et al., 2002; Verma et al., 2002; Yao and Cohen, 2002). Correlations of proteome turnover profiles also revealed new functional relationships, for example:

Hel2-Mag2. Hel2 was initially identified as an E3 that targets excess histones for degradation (Singh et al., 2012). In addition, Hel2 and its human homolog ZNF598 are involved in RQC, where Hel2-mediated ubiquitination of the 40S small ribosomal subunit within a collided di-ribosome is required to trigger RQC (Ikeuchi et al., 2019; Juszkiewicz and Hegde, 2017; Juszkiewicz et al., 2018; Matsuo et al., 2017; Sitron et al., 2017; Sundaramoorthy et al., 2017; Winz et al., 2019). Accordingly, turnover of histones and components of the 40S ribosome was affected in the *hel2*Δ mutant (Figure S6A). This phenotype correlated with phenotypes of mutants lacking Ubc4, the E2 enzyme that interacts with Hel2 (Singh et al., 2012), and Mag2 (Figures 4A and S6A). Mag2 is a poorly characterized RING E3 that, together with Hel2 and the E3 Rsp5, was recently implicated in degradation of non-functional 18S rRNA in a process that also involves ubiquitination of the 40S small ribosomal subunit (Sugiyama et al., 2019). Lack of Mag2 resulted in stabilization of several ribosome components, specifically of the 40S ribosome (Figure S6B). However, this phenotype was anticorrelated with that of *rsp5* mutants (Figure S6A), suggesting that, besides its role in degradation of non-functional 18S rRNA, Mag2 could also be involved in RQC.

Hel1-SCF$^{\mathbf{Hrt3}}$. Another set of correlations involved components of the cullin-RING E3 SCF (the cullin 1 Cdc53 and the F-box substrate adaptor Hrt3), the neddylation machinery (the ubiquitin-like modifier Rub1/Nedd8, the cognate E2 Ubc12, and the DUB Yuh1), the RING-IBR-RING (RBR) E3 of the Ariadne family Hel1, the RING E3 Nam7, and the DUB Ubp10 (Figures 4A and S6C–S6F). Although neddylation of cullins is required for robust activity of cullin-RING E3s, the neddylation machinery is not essential in yeast (Lammer et al., 1998; Liakopoulos et al., 1998; Willems et al., 2004). Accordingly, few proteins were affected in the *rub1*Δ and *yuh1*Δ strains compared with the *cdc53-1* mutant (Figures S2G and S2H). The phenotypes of the *rub1*Δ and *yuh1*Δ mutants were largely restricted to stabilization of the translation elongation factor 2 (eEF2), which, in yeast, is encoded by two paralogs, *EFT1* and *EFT2.* In fact, the whole set of correlations was mostly driven by changes in the stability of Eft1 and Eft2 (Figures S6C–S6F). Interestingly, Eft1-tFT was less affected in logarithmically growing cultures compared with colonies (Figure S6G), indicating conditional regulation of eEF2. Using Eft1-tFT stability in colonies as a readout, we further assessed the relationships between these correlating UPS components. Eft1 destabilization in the *ubp10*Δ and *nam7*Δ mutants depended on Hel1 and Hrt3, placing these factors in one pathway (Figure S6H). Deletion of *HRT3* or *HEL1* stabilized Eft1-tFT to a similar extent, and no further stabilization was detected in the *hrt3*Δ *hel1*Δ double mutant, suggesting that Hel1 and SCF^{Hrt3} cooperate in substrate ubiquitination. In human cells and in *C. elegans*, cullin-RING E3s associate and work together with an Ariadne family RBR, ARIH1/HHARI (human homolog of *Drosophila* Ariadne-1) (Dove et al., 2017; Scott et al., 2016). Our results point toward similar cooperation between Hel1 and SCF^{Hrt3} in yeast, possibly in conditional control of translation.

GID complex. The GID E3 is involved in degradation of gluconeogenic enzymes when switching from gluconeogenesis to glycolysis (Regelmann et al., 2003; Santt et al., 2008). Only one of its substrates, the malate dehydrogenase Mdh2 (Hung et al., 2004; Santt et al., 2008), was in the tFT library. Mdh2 was stabilized in the absence of Gid2 or Gid9, the two RING subunits, and in cells lacking Ubc8, the E2 that works with GID (Figure 5A). Ubc8 appears to function exclusively with the GID E3 under the screen conditions because the phenotypes of *ubc8*Δ, *gid2*Δ and *gid9*Δ mutants were almost identical (Figures 4A and S7A). The DUB Ubp14 has been implicated previously in degradation of GID substrates (Eisele et al., 2006; Regelmann et al., 2003). However, not all potential GID substrates were affected in the *ubp14*Δ mutant (Figure 5A). The *ubp14*Δ and *gid2*Δ phenotypes were not additive (Figure S7B), indicating that Ubp14 promotes protein degradation with the GID E3 but in a substrate-specific manner.

In total, 31 proteins were stabilized in the *gid2*Δ, *gid9*Δ, or *ubc8*Δ mutants (Figure 5A). These included several GID subunits and three potential GID substrates (Aro10, Tma10, and Stf2) identified in a recent proteomic study (Karayel et al., 2020). For most tFT fusions, their stabilization was detectable in colonies, which consist of cells in different metabolic states (Čáp et al., 2012), but not in logarithmically growing cultures with glucose as a carbon source (Figure 5B). This is consistent with the idea that GID regulates protein turnover in metabolic transitions or in response to stress (Melnykov et al., 2019) and highlights how colony-based proteome profiling can reveal conditional phenotypes. Metabolic heterogeneity of colonies also complicates direct comparison with pulse SILAC proteomic profiling of the UPS (Christiano et al., 2020). GID-dependent turnover of Mdh2, Ydr222w, and several GID subunits in log-phase cultures (Figure 5B) suggests that the GID E3 is also active under steady-state conditions, consistent with previous observations (Menssen et al., 2018).

Next we examined the role of other GID components in turnover of potential GID substrates. All tested tFT fusions were stabilized in mutants lacking any single GID subunit except Gid7 (Figure 5C). In contrast, loss of Ipf1 or Moh1, which co-purify with the GID complex (Ho et al., 2002; Subbotin and Chait, 2014), did not affect any tFT fusions (Figures S7C and S7D).

CellPress
OPEN ACCESS

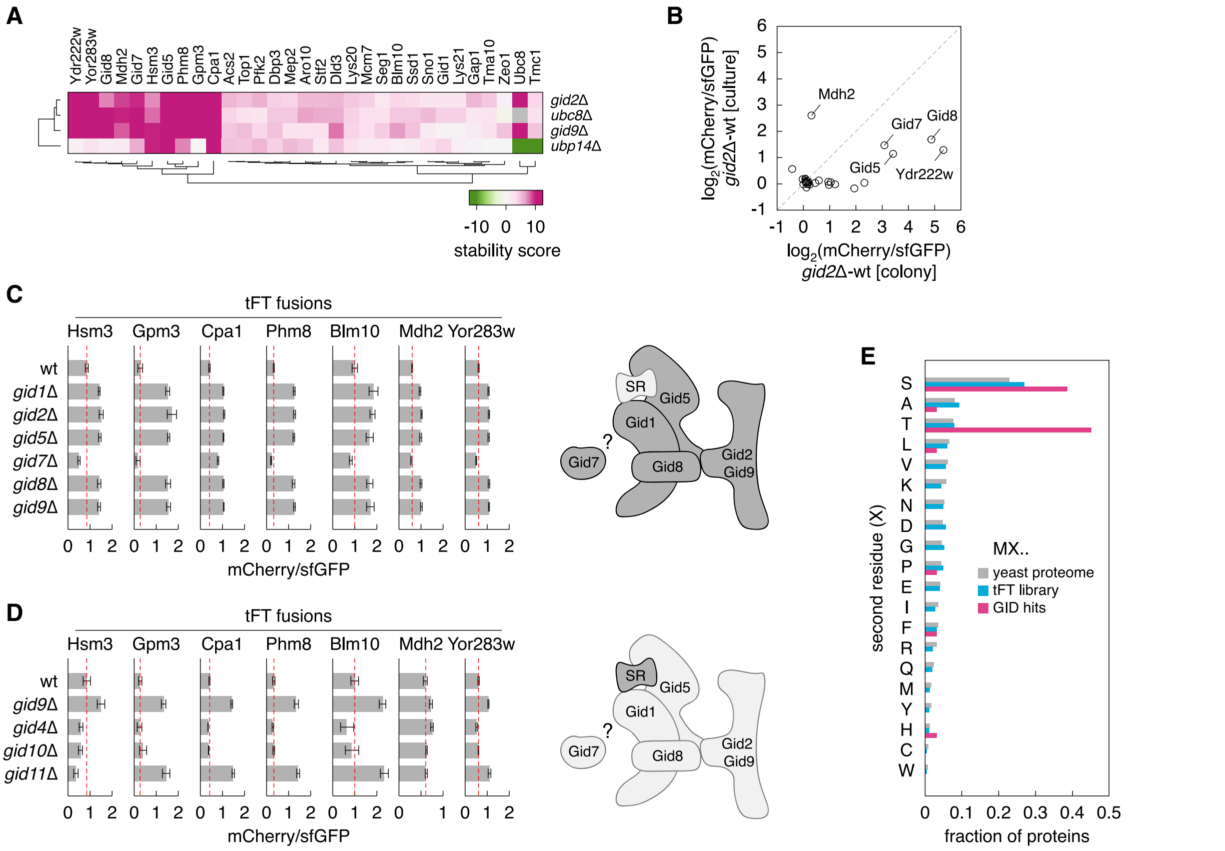

Figure 5. Protein turnover by the GID complex

(A) Heatmap of protein stability changes in the absence of GID components (screens in Figure 2). Only proteins stabilized in at least one of the mutants (*gid2*Δ, *gid9*Δ, and *ubc8*Δ; 1% FDR, stability score >4) are shown; their behavior in the *ubp14*Δ mutant is included for comparison.

(B) Differences in mCherry/sfGFP ratios between *gid2*Δ and WT cells for tFT-tagged proteins from (A). Flow cytometry measurements of log-phase cultures and plate reader measurements of colonies (mean, n = 4). Proteins stabilized in the *gid2*Δ background in cultures are indicated.

(C and D) mCherry/sfGFP ratios of colonies expressing tFT fusions and lacking GID components (mean ± SD, n = 4) and cartoon of the GID complex; SR, substrate receptor (right; adapted from Qiao et al., 2020).

(E) Frequency of residues at the second position in potential GID substrates from (A) (GID hits).

See also Figure S7 and Table S5.

Lack of protein stabilization in the *gid7*Δ mutant is consistent with the reported structure of a functional GID complex, which does not include Gid7 (Qiao et al., 2020). Nevertheless, it is possible that a variant GID complex containing Gid7 exists, considering that Gid7 is involved in Gid4-depedent turnover of Fbp1 (Regelmann et al., 2003), and correlations of genetic interaction profiles indicate that *GID7* is functionally related to core GID subunits (Costanzo et al., 2016; Figure S7E). Moreover, turnover of Gid7-tFT was affected in various *gid* mutants, similar to other GID subunits (Figure S7D).

GID recognizes substrates via N-degrons using the interchangeable receptor subunits Gid4 and Gid10 (Chen et al., 2017; Melnykov et al., 2019; Qiao et al., 2020). Although Gid4 recognizes substrates such as Mdh2 via N-degrons with an N-terminal proline (Chen et al., 2017; Hämmerle et al., 1998), the partially overlapping specificity of Gid10 is less understood (Melnykov et al., 2019). In the case of Mdh2, proline is exposed at the N terminus after co-translational removal of the initiator methionine by methionine aminopeptidases (MetAPs), whose substrates comprise N termini with small residues (A, S, T, V, C, G, P) after the initiator methionine (Moerschell et al., 1990; Varland et al., 2015). As expected, Mdh2 was stabilized in the absence of Gid4 in the tFT assay. However, deletion of *GID4* or *GID10* did not affect any tested potential GID substrates (Figure 5D).

Interestingly, 26 of the 31 potential GID substrates have a serine or a threonine after the initiator methionine (Figure 5E; Table S5). We thus asked whether an unknown GID receptor was involved in their recognition. To identify such a receptor, we performed genetic screens with two potential substrates, the carbamoyl phosphate synthetase Cpa1 (N terminus MSSAA) and the nucleotidase Phm8 (N terminus MTIAK). We crossed *CPA1-tFT* and *PHM8-tFT* strains with a genome-wide knockout library (Winzeler et al., 1999) and measured the mCherry and sfGFP fluorescence of the resulting colonies to identify mutants that increased the abundance and stability of each fusion. In the control screen with Mdh2-tFT, we identified mutants of *UBC8* and of all known GID subunits except *GID9* (absent from the knockout

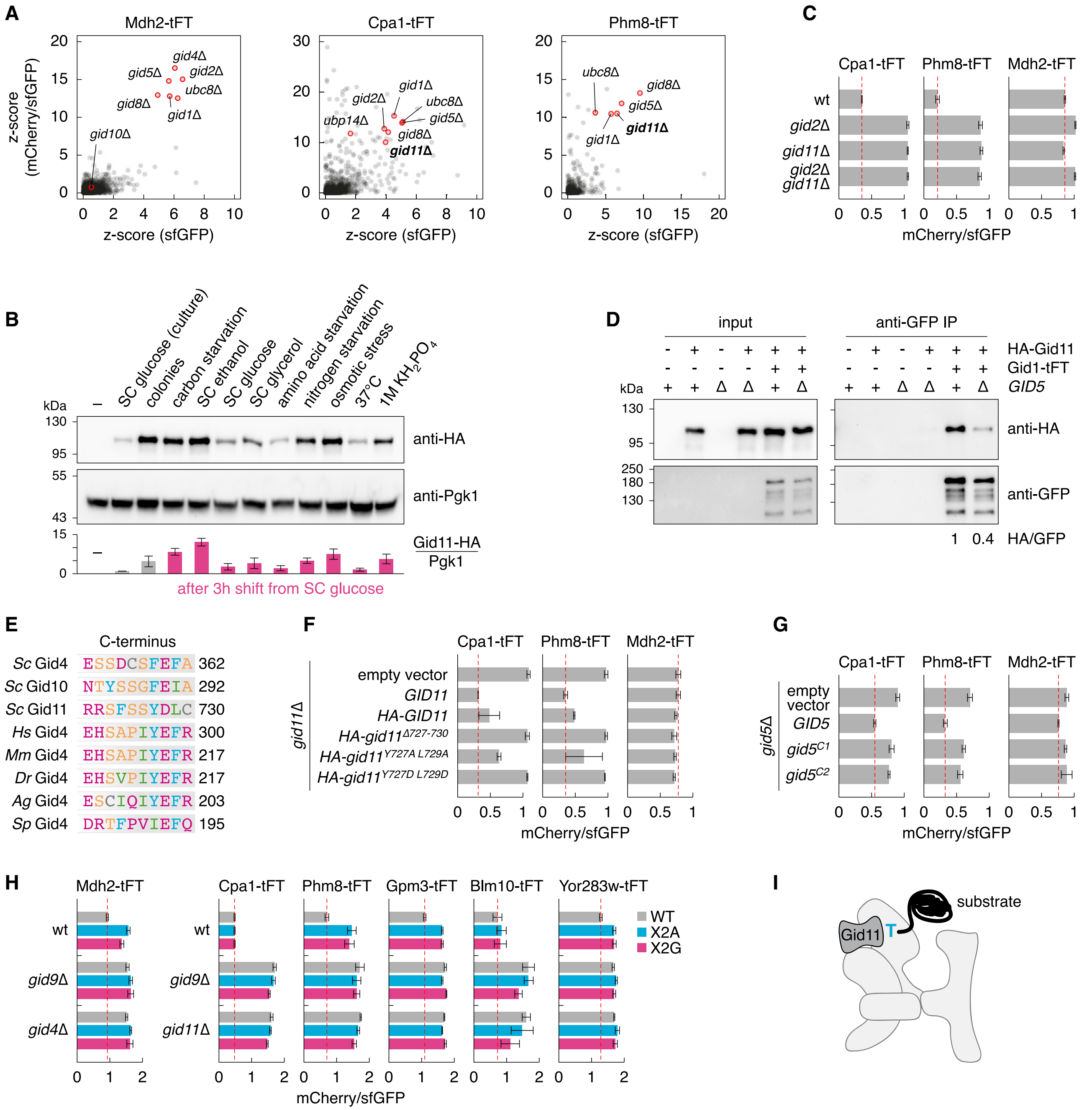

Figure 6. Gid11-dependent turnover of proteins with an N-terminal threonine

(A) Genome-wide screens for factors involved in turnover of Cpa1 and Phm8. Only gene deletions with positive *Z* scores are shown.

(B) Immunoblot of strains expressing chromosomally tagged Gid11-HA (top) and quantification of relative Gid11-HA expression levels (bottom, mean ± SD, n ≥ 3). Samples were collected from log-phase cultures in synthetic complete medium with glucose as a carbon source (SC glucose) from colonies or after a 3-h shift from SC glucose into the indicated environment (red).

(C) mCherry/sfGFP ratios of colonies expressing tFT fusions and lacking *GID2* and/or *GID11* (mean ± SD, n = 4).

(D) Co-immunoprecipitation of overexpressed HA-Gid11 and chromosomally tagged Gid1-tFT. The relative amount of co-immunoprecipitated HA-Gid11, normalized to precipitated Gid1-tFT, was reduced to 0.43 ± 0.15 (mean ± SD, n = 3) in *gid5*Δ cells compared with the WT.

(E) C termini of GID receptors from different organisms. Sc, *S. cerevisiae*; Hs, *Homo sapiens*; Mm, *Mus musculus*; Dr, *Danio rerio*; Ag, *Anopheles gambiae*; Sp, *Schizosaccharomyces pombe*.

(F and G) mCherry/sfGFP ratios of colonies expressing tFT fusions (mean ± SD, n = 3 [F] or n = 4 [G]). Dashed lines mark mCherry/sfGFP ratios in *gid11*Δ or *gid5*Δ mutants complemented with WT *GID11* or *GID5*, respectively.

(legend continued on next page)

library), *GID7* and *GID10* (Figure 6A). Among the top hits for Cpa1-tFT and Phm8-tFT were mutants of several GID subunits and a strain lacking *YLR149C*, a gene of unknown function that we named *GID11* (Figure 6A; Table S6). Gid11 is a WD40/YVTN repeat-like domain protein conserved across yeasts (Figure S7F; Table S7). Gid11 expression was higher in colonies compared with log-phase cultures with glucose as a carbon source and was upregulated by using ethanol as a carbon source or by various stresses, including carbon starvation, nitrogen starvation, and hyperosmotic stress (Figure 6B). This is consistent with the conditional nature of GID phenotypes (Figure 5B).

Although the genetic interaction profile of *GID11* correlated poorly with other *GID* genes (Figure S7E), deletion of *GID11* stabilized Cpa1-tFT and Phm8-tFT to the same extent as deletion of *GID2*, and no further stabilization was seen in the *gid2*Δ *gid11*Δ double mutant (Figure 6C). This suggests that Gid11 functions with the GID E3. We thus tested whether Gid11 interacts with GID subunits in co-immunoprecipitation experiments. Using strains expressing tFT-tagged GID subunits and HA-Gid11 overexpression during logarithmic growth, we detected an interaction between Gid11 and Gid1 (Figures 6D and S7G). This interaction was dependent on Gid5 (Figure 6D), which also recruits the Gid4 and Gid10 receptors to the GID complex (Melnykov et al., 2019; Menssen et al., 2012; Qiao et al., 2020).

Gid4 and Gid10 interact with Gid5 via C-terminal anchors that end with a conserved ΦEΦX motif (where Φ denotes a hydrophobic residue) (Qiao et al., 2020; Figure 6E). Gid11 has a similar C-terminal YDLC motif that only differs in the acidic residue D instead of E (Figures 6E and S7F), raising the possibility of an analogous mode of interaction between Gid11 and Gid5. To test this, we first examined how mutations in the Gid11 C terminus affect its function. Although plasmid-borne expression of HA-Gid11 could complement a *gid11*Δ mutant and restore turnover of Cpa1-tFT and Phm8-tFT, deleting the last 4 residues in Gid11 ($\text{Gid11}^{\Delta 727-730}$) or replacing the C-terminal hydrophobic residues with charged ones ($\text{Gid11}^{\text{Y727D L729D}}$) resulted in nonfunctional Gid11 variants (Figure 6F). Second, the Gid4 C-terminal anchor interacts with the C-terminal domain of Gid5. Gid5 variants with mutations at this interaction interface, such as Gid5^{C1} (W606A, H610A, and Y613A) and Gid5^{C2} (W606A, Y613A, and Q649A), show impaired Gid4-dependent ubiquitination and turnover of gluconeogenic enzymes (Qiao et al., 2020). The Gid5^{C1} and Gid5^{C2} mutants were also defective in Gid11-dependent turnover of Cpa1 and Phm8 (Figure 6G). Finally, fusing the last 20 residues of Gid11 to luciferase (HA-Luc-$\text{Gid11}^{\text{C20}}$) was sufficient to recruit it to the GID complex, as evidenced by co-immunoprecipitation of HA-Luc-$\text{Gid11}^{\text{C20}}$ with Gid1 (Figure S7H). These results argue that Gid11 is recruited to the GID complex by a C-terminal anchor that interacts with Gid5, similar to the Gid4 and Gid10 receptors.

Next we determined the spectrum of Gid11 substrates. Using the tFT assay, we tested whether turnover of potential GID substrates (Figure 5A) depends on *GID11*. Remarkably, 12 of the 14 proteins with an N-terminal threonine were stabilized in the absence of Gid11 to the same extent as in a *gid9*Δ mutant (Figures 5D and S7I). The only exceptions were two GID subunits, Gid5 and Gid8. In contrast, of the 16 proteins with residues other than threonine after the initiator methionine, only Cpa1 showed Gid11-dependent turnover (Figure S7I). Therefore, we tested how Gid11-dependent protein turnover depends on the substrate N terminus. As controls, we mutated the N-terminal proline of Mdh2 to alanine (Mdh2^{P2A}) or glycine residues (Mdh2^{P2G}), which do not prevent removal of the initiator methionine by MetAPs (Moerschell et al., 1990; Varland et al., 2015). Both mutations preclude Gid4 binding to the Mdh2 N terminus and largely abolished Gid4-dependent turnover of Mdh2-tFT (Chen et al., 2017; Hämmerle et al., 1998; Figure 6H). The same mutations in Cpa1 and Blm10 had no obvious effect on their turnover. It is possible that Cpa1 and Blm10 (N terminus MTANN) stabilization in *gid* mutants is indirect or that their recognition is more complex. Nevertheless, Phm8, Gpm3, and Yor283w variants with the N-terminal threonine mutated to alanine or glycine were stable compared with wild-type proteins and were not stabilized further by deletion of *GID9* or *GID11* (Figure 6H), indicating that their N termini carry degrons recognized by the GID complex. In addition, we replaced the N terminus of Phm8 with N termini of other GID substrates. Only sequences with an N-terminal threonine allowed Gid11-dependent turnover of Phm8 (Figure S7J). These results suggest that Gid11 is a substrate receptor of the GID complex that recognizes degrons with an N-terminal threonine (Figure 6I). The precise N-degron motif bound by Gid11 and the role of MetAPs and N-terminal acetyltransferases, which should modify such N termini, in Gid11-dependent protein turnover remain to be determined.

The vertebrate GID/CTLH (C-terminal to LisH) complex is linked to a variety of processes, including cilium function, the cell cycle, and metabolism (Boldt et al., 2016; Lampert et al., 2018; Leal-Esteban et al., 2018; Liu and Pfirrmann, 2019; Liu et al., 2020; Pfirrmann et al., 2015; Texier et al., 2014). Yeast and human Gid4 appear to recognize similar N-degrons, although no substrates of human Gid4 are known (Chen et al., 2017; Dong et al., 2018, 2020), and N termini of known substrates do not fit the Gid4 consensus (Lampert et al., 2018). It will therefore be important to understand how the expanded specificity of the GID complex described here translates outside of yeast.

DISCUSSION

As the key system of selective protein degradation, the UPS is connected to all cellular processes by destroying unnecessary or abnormal proteins at the right time and place (Hershko and Ciechanover, 1998; Kleiger and Mayor, 2014; Zheng and Shabek, 2017). This work provides a rich dataset to explore different

(H) mCherry/sfGFP ratios of colonies expressing tFT-tagged proteins, either WT or with the second residue X mutated to alanine (X2A) or glycine (X2G) (mean ± SD, n ≥ 3).
(I) Model of Gid11 as a receptor for substrates with an N-terminal threonine, exposed after removal of the initiator methionine by MetAPs. Gid11-dependent protein turnover requires all core GID subunits but not Gid7.
See also Figure S7 and Tables S6 and S7.

functions of this system from the perspective of a protein of interest or with a UPS component as a starting point.

The proteomic approach applied here, although arguably more laborious compared with mass spectrometry-based proteomics (Christiano et al., 2020), has several advantages. First, because protein abundance and stability were measured in yeast colonies, which consist of metabolically heterogeneous populations (Cáp et al., 2012), our proteome profiling is sampling multiple environmental conditions in one experiment. This is evidenced by various conditional phenotypes identified in the screen. Second, the strains generated for proteome profiling could be used in other downstream analyses, including studies of noise in protein degradation or analysis of protein localization changes upon perturbation of selective protein degradation. Although proteome profiling is ideally suited to investigate mechanisms of selective protein degradation, this approach could be extended to other cellular processes as an unbiased phenotypic assay to characterize gene functions. Ultimately, integrating different phenotypic profiles, including genetic interactions and changes in transcriptome, proteome, and intracellular organization, should refine functional predictions and help dissect complex cellular processes.

Limitations of study

Several protein classes known to be affected by C-terminal tagging, such as tail-anchored or glycosylphosphatidylinositol-anchored proteins, are excluded from the tFT library (Khmelinskii et al., 2014). Tagging could still impair turnover of some proteins (e.g., by blocking C-terminal degrons; Koren et al., 2018; Lin et al., 2018) or promote turnover of others because of the large size of the tFT tag, as seen with Ubr1-dependent Mcr1 turnover. In addition, potential substrates identified for various UPS components should be validated independently to rule out indirect effects or adaption of UPS mutants. Finally, the number of identified potential substrates is likely limited by redundancies within the UPS.

STAR★METHODS

Detailed methods are provided in the online version of this paper and include the following:

- KEY RESOURCES TABLE
- RESOURCE AVAILABILITY
  - Lead contact
  - Materials availability
  - Data and code availability
- EXPERIMENTAL MODEL AND SUBJECT DETAILS
- METHOD DETAILS
  - Immunoblotting
  - Flow cytometry
  - Measurements of proteome abundance and turnover with the tFT library
  - Profiling of the ubiquitin-proteasome system
  - Genome-wide screens for factors affecting protein stability
  - Analysis of Gid11 conservation
  - Co-immunoprecipitation
- QUANTIFICATION AND STATISTICAL ANALYSIS

SUPPLEMENTAL INFORMATION

Supplemental information can be found online at https://doi.org/10.1016/j.molcel.2021.04.018.

ACKNOWLEDGMENTS

We thank John Lindner, Balca Mardin, Sebastian Schuck, Helle Ulrich, and Fridolin Kielisch for comments on the manuscript and the ZMBH flow cytometry facility and IMB media lab for assistance with experiments. This work was supported by the Deutsche Forschungsgemeinschaft (SFB1036/TP10 to M.K. and A.K.), the CellNetworks Cluster of Excellence (to M.K.), the European Molecular Biology Organization (EMBO ALTF 1124-2010 and EMBO ASTF 546-2012 to A.K.), the European Research Council (ERC-2017-STG#759427 to A.K.), the collaborative research project "SOUND" funded by European Commission H2020 Program (grant agreement 633974), the Canadian Institutes of Health Research (FDN-143264 and FDN-143265 to B.J.A. and C.B., respectively), and the National Institutes of Health (R01HG005853). C.B. holds a Canada Research Chair (Tier 1) in Proteomics, Bioinformatics, and Functional Genomics.

AUTHOR CONTRIBUTIONS

Conceptualization, M.K. and A.K.; methodology and investigation, K.-Y.E.K., B.F., M.M., I.K., J.D.B., D.K., V.C., B.-J.S.L., Z.L., M.C., W.H., B.J.A., C.B., M.K., and A.K.; formal analysis, B.F., F.R., J.D.B., and A.K.; writing – original draft, A.K.; writing – review and editing, K.-Y.E.K., M.K., and A.K.; supervision, M.K. and A.K.; funding acquisition, B.J.A., C.B., W.H., M.K., and A.K.

DECLARATION OF INTERESTS

The authors declare no competing interests.

Received: August 10, 2020
Revised: March 15, 2021
Accepted: April 19, 2021
Published: May 10, 2021

REFERENCES

Bachmair, A., and Varshavsky, A. (1989). The degradation signal in a short-lived protein. Cell *56*, 1019–1032.

Bachmair, A., Finley, D., and Varshavsky, A. (1986). In vivo half-life of a protein is a function of its amino-terminal residue. Science *234*, 179–186.

Baker, R.T., and Varshavsky, A. (1995). Yeast N-terminal amidase. A new enzyme and component of the N-end rule pathway. J. Biol. Chem. *270*, 12065–12074.

Balch, W.E., Morimoto, R.I., Dillin, A., and Kelly, J.W. (2008). Adapting proteostasis for disease intervention. Science *319*, 916–919.

Balchin, D., Hayer-Hartl, M., and Hartl, F.U. (2016). In vivo aspects of protein folding and quality control. Science *353*, aac4354.

Balzi, E., Choder, M., Chen, W.N., Varshavsky, A., and Goffeau, A. (1990). Cloning and functional analysis of the arginyl-tRNA-protein transferase gene ATE1 of Saccharomyces cerevisiae. J. Biol. Chem. *265*, 7464–7471.

Barros, G.C., Requião, R.D., Carneiro, R.L., Masuda, C.A., Moreira, M.H., Rossetto, S., Domitrovic, T., and Palhano, F.L. (2021). Rqc1 and other yeast proteins containing highly positively charged sequences are not targets of the RQC complex. J. Biol. Chem. Published online March 24, 2021. https://doi.org/10.1016/j.jbc.2021.100586.

Baryshnikova, A., Costanzo, M., Kim, Y., Ding, H., Koh, J., Toufighi, K., Youn, J.-Y.Y., Ou, J., San Luis, B.-J.J., Bandyopadhyay, S., et al. (2010a). Quantitative analysis of fitness and genetic interactions in yeast on a genome scale. Nat. Methods *7*, 1017–1024.

Baryshnikova, A., Costanzo, M., Dixon, S., Vizeacoumar, F.J., Myers, C.L., Andrews, B., and Boone, C. (2010b). Synthetic genetic array (SGA) analysis

CellPress
OPEN ACCESS

in Saccharomyces cerevisiae and Schizosaccharomyces pombe. Methods Enzymol. *470*, 145–179.

Belle, A., Tanay, A., Bitincka, L., Shamir, R., and O'Shea, E.K. (2006). Quantification of protein half-lives in the budding yeast proteome. Proc. Natl. Acad. Sci. USA *103*, 13004–13009.

Bengtson, M.H., and Joazeiro, C.A.P. (2010). Role of a ribosome-associated E3 ubiquitin ligase in protein quality control. Nature *467*, 470–473.

Boisvert, F.M., Ahmad, Y., Gierliński, M., Charrière, F., Lamont, D., Scott, M., Barton, G., and Lamond, A.I. (2012). A quantitative spatial proteomics analysis of proteome turnover in human cells. Mol. Cell. Proteomics *11*, 011429.

Boldt, K., van Reeuwijk, J., Lu, Q., Koutroumpas, K., Nguyen, T.M.T., Texier, Y., van Beersum, S.E.C., Horn, N., Willer, J.R., Mans, D.A., et al.; UK10K Rare Diseases Group (2016). An organelle-specific protein landscape identifies novel diseases and molecular mechanisms. Nat. Commun. *7*, 11491.

Brachmann, C.B., Davies, A., Cost, G.J., Caputo, E., Li, J., Hieter, P., and Boeke, J.D. (1998). Designer deletion strains derived from Saccharomyces cerevisiae S288C: a useful set of strains and plasmids for PCR-mediated gene disruption and other applications. Yeast *14*, 115–132.

Bragoszewski, P., Wasilewski, M., Sakowska, P., Gornicka, A., Böttinger, L., Qiu, J., Wiedemann, N., and Chacinska, A. (2015). Retro-translocation of mitochondrial intermembrane space proteins. Proc. Natl. Acad. Sci. USA *112*, 7713–7718.

Brandman, O., Stewart-Ornstein, J., Wong, D., Larson, A., Williams, C.C., Li, G.-W.W., Zhou, S., King, D., Shen, P.S., Weibezahn, J., et al. (2012). A ribosome-bound quality control complex triggers degradation of nascent peptides and signals translation stress. Cell *151*, 1042–1054.

Cambridge, S.B., Gnad, F., Nguyen, C., Bermejo, J.L., Krüger, M., and Mann, M. (2011). Systems-wide proteomic analysis in mammalian cells reveals conserved, functional protein turnover. J. Proteome Res. *10*, 5275–5284.

Cáp, M., Stěpánek, L., Harant, K., Váchová, L., and Palková, Z. (2012). Cell differentiation within a yeast colony: metabolic and regulatory parallels with a tumor-affected organism. Mol. Cell *46*, 436–448.

Chen, S.J., Wu, X., Wadas, B., Oh, J.H., and Varshavsky, A. (2017). An N-end rule pathway that recognizes proline and destroys gluconeogenic enzymes. Science *355*, eaal3655.

Christiano, R., Nagaraj, N., Fröhlich, F., and Walther, T.C. (2014). Global proteome turnover analyses of the Yeasts S. cerevisiae and S. pombe. Cell Rep. *9*, 1959–1965.

Christiano, R., Arlt, H., Kabatnik, S., Mejhert, N., Lai, Z.W., Farese, R.V., Jr., and Walther, T.C. (2020). A Systematic Protein Turnover Map for Decoding Protein Degradation. Cell Rep. *33*, 108378.

Costanzo, M., VanderSluis, B., Koch, E.N., Baryshnikova, A., Pons, C., Tan, G., Wang, W., Usaj, M., Hanchard, J., Lee, S.D., et al. (2016). A global genetic interaction network maps a wiring diagram of cellular function. Science *353*, aaf1420.

de Godoy, L.M.F., Olsen, J.V., Cox, J., Nielsen, M.L., Hubner, N.C., Fröhlich, F., Walther, T.C., and Mann, M. (2008). Comprehensive mass-spectrometry-based proteome quantification of haploid versus diploid yeast. Nature *455*, 1251–1254.

Dederer, V., Khmelinskii, A., Huhn, A.G., Okreglak, V., Knop, M., and Lemberg, M.K. (2019). Cooperation of mitochondrial and ER factors in quality control of tail-anchored proteins. eLife *8*, e45506.

Dephoure, N., Hwang, S., O'Sullivan, C., Dodgson, S.E., Gygi, S.P., Amon, A., and Torres, E.M. (2014). Quantitative proteomic analysis reveals posttranslational responses to aneuploidy in yeast. eLife *3*, e03023.

Dong, C., Zhang, H., Li, L., Tempel, W., Loppnau, P., and Min, J. (2018). Molecular basis of GID4-mediated recognition of degrons for the Pro/N-end rule pathway. Nat. Chem. Biol. *14*, 466–473.

Dong, C., Chen, S.J., Melnykov, A., Weirich, S., Sun, K., Jeltsch, A., Varshavsky, A., and Min, J. (2020). Recognition of nonproline N-terminal residues by the Pro/N-degron pathway. Proc. Natl. Acad. Sci. USA *117*, 14158–14167.

Dove, K.K., Kemp, H.A., Di Bona, K.R., Reiter, K.H., Milburn, L.J., Camacho, D., Fay, D.S., Miller, D.L., and Klevit, R.E. (2017). Two functionally distinct E2/E3 pairs coordinate sequential ubiquitination of a common substrate in *Caenorhabditis elegans* development. Proc. Natl. Acad. Sci. USA *114*, E6576–E6584.

Dujon, B. (2010). Yeast evolutionary genomics. Nat. Rev. Genet. *11*, 512–524.

Edgar, R.C. (2004). MUSCLE: multiple sequence alignment with high accuracy and high throughput. Nucleic Acids Res. *32*, 1792–1797.

Eisele, F., Braun, B., Pfirrmann, T., and Wolf, D.H. (2006). Mutants of the deubiquitinating enzyme Ubp14 decipher pathway diversity of ubiquitin-proteasome linked protein degradation. Biochem. Biophys. Res. Commun. *350*, 329–333.

Ella, H., Reiss, Y., and Ravid, T. (2019). The hunt for degrons of the 26S proteasome. Biomolecules *9*, 230.

Finley, D., Ulrich, H.D., Sommer, T., and Kaiser, P. (2012). The ubiquitin-proteasome system of Saccharomyces cerevisiae. Genetics *192*, 319–360.

Foresti, O., Rodriguez-Vaello, V., Funaya, C., and Carvalho, P. (2014). Quality control of inner nuclear membrane proteins by the Asi complex. Science *346*, 751–755.

Francis, O., Han, F., and Adams, J.C. (2013). Molecular phylogeny of a RING E3 ubiquitin ligase, conserved in eukaryotic cells and dominated by homologous components, the muskelin/RanBPM/CTLH complex. PLoS ONE *8*, e75217.

Ghaemmaghami, S., Huh, W.-K., Bower, K., Howson, R.W., Belle, A., Dephoure, N., O'Shea, E.K., and Weissman, J.S. (2003). Global analysis of protein expression in yeast. Nature *425*, 737–741.

Greene, A.W., Grenier, K., Aguileta, M.A., Muise, S., Farazifard, R., Haque, M.E., McBride, H.M., Park, D.S., and Fon, E.A. (2012). Mitochondrial processing peptidase regulates PINK1 processing, import and Parkin recruitment. EMBO Rep. *13*, 378–385.

Hahne, K., Haucke, V., Ramage, L., and Schatz, G. (1994). Incomplete arrest in the outer membrane sorts NADH-cytochrome b5 reductase to two different submitochondrial compartments. Cell *79*, 829–839.

Hämmerle, M., Bauer, J., Rose, M., Szallies, A., Thumm, M., Düsterhus, S., Mecke, D., Entian, K.D., and Wolf, D.H. (1998). Proteins of newly isolated mutants and the amino-terminal proline are essential for ubiquitin-proteasome-catalyzed catabolite degradation of fructose-1,6-bisphosphatase of Saccharomyces cerevisiae. J. Biol. Chem. *273*, 25000–25005.

Hanna, J., Hathaway, N.A., Tone, Y., Crosas, B., Elsasser, S., Kirkpatrick, D.S.S., Leggett, D.S., Gygi, S.P., King, R.W., and Finley, D. (2006). Deubiquitinating enzyme Ubp6 functions noncatalytically to delay proteasomal degradation. Cell *127*, 99–111.

Harper, J.W., Ordureau, A., and Heo, J.M. (2018). Building and decoding ubiquitin chains for mitophagy. Nat. Rev. Mol. Cell Biol. *19*, 93–108.

Hershko, A., and Ciechanover, A. (1998). The ubiquitin system. Annu. Rev. Biochem. *67*, 425–479.

Ho, Y., Gruhler, A., Heilbut, A., Bader, G.D., Moore, L., Adams, S.L., Millar, A., Taylor, P., Bennett, K., Boutilier, K., et al. (2002). Systematic identification of protein complexes in Saccharomyces cerevisiae by mass spectrometry. Nature *415*, 180–183.

Hofmann, R.M., and Pickart, C.M. (1999). Noncanonical MMS2-encoded ubiquitin-conjugating enzyme functions in assembly of novel polyubiquitin chains for DNA repair. Cell *96*, 645–653.

Huerta-Cepas, J., Capella-Gutiérrez, S., Pryszcz, L.P., Marcet-Houben, M., and Gabaldón, T. (2014). PhylomeDB v4: zooming into the plurality of evolutionary histories of a genome. Nucleic Acids Res. *42*, D897–D902.

Huh, W.-K., Falvo, J.V., Gerke, L.C., Carroll, A.S., Howson, R.W., Weissman, J.S., and O'Shea, E.K. (2003). Global analysis of protein localization in budding yeast. Nature *425*, 686–691.

Hung, G.-C., Brown, C.R., Wolfe, A.B., Liu, J., and Chiang, H.-L. (2004). Degradation of the gluconeogenic enzymes fructose-1,6-bisphosphatase and malate dehydrogenase is mediated by distinct proteolytic pathways and signaling events. J. Biol. Chem. *279*, 49138–49150.

Ikeuchi, K., Tesina, P., Matsuo, Y., Sugiyama, T., Cheng, J., Saeki, Y., Tanaka, K., Becker, T., Beckmann, R., and Inada, T. (2019). Collided ribosomes form a unique structural interface to induce Hel2-driven quality control pathways. EMBO J. *38*, e100276.

Janke, C., Magiera, M.M., Rathfelder, N., Taxis, C., Reber, S., Maekawa, H., Moreno-Borchart, A., Doenges, G., Schwob, E., Schiebel, E., and Knop, M. (2004). A versatile toolbox for PCR-based tagging of yeast genes: new fluorescent proteins, more markers and promoter substitution cassettes. Yeast *21*, 947–962.

Jin, S.M., Lazarou, M., Wang, C., Kane, L.A., Narendra, D.P., and Youle, R.J. (2010). Mitochondrial membrane potential regulates PINK1 import and proteolytic destabilization by PARL. J. Cell Biol. *191*, 933–942.

Joazeiro, C.A.P. (2019). Mechanisms and functions of ribosome-associated protein quality control. Nat. Rev. Mol. Cell Biol. *20*, 368–383.

Juszkiewicz, S., and Hegde, R.S. (2017). Initiation of Quality Control during Poly(A) Translation Requires Site-Specific Ribosome Ubiquitination. Mol. Cell *65*, 743–750.e4.

Juszkiewicz, S., Chandrasekaran, V., Lin, Z., Kraatz, S., Ramakrishnan, V., and Hegde, R.S. (2018). ZNF598 Is a Quality Control Sensor of Collided Ribosomes. Mol. Cell *72*, 469–481.e7.

Käll, L., Krogh, A., and Sonnhammer, E.L.L. (2004). A combined transmembrane topology and signal peptide prediction method. J. Mol. Biol. *338*, 1027–1036.

Karayel, O., Michaelis, A.C., Mann, M., Schulman, B.A., and Langlois, C.R. (2020). DIA-based systems biology approach unveils E3 ubiquitin ligase-dependent responses to a metabolic shift. Proc. Natl. Acad. Sci. USA *117*, 32806–32815.

Kemmeren, P., Sameith, K., van de Pasch, L.A.L., Benschop, J.J., Lenstra, T.L., Margaritis, T., O'Duibhir, E., Apweiler, E., van Wageningen, S., Ko, C.W., et al. (2014). Large-scale genetic perturbations reveal regulatory networks and an abundance of gene-specific repressors. Cell *157*, 740–752.

Khmelinskii, A., and Knop, M. (2014). Analysis of protein dynamics with tandem fluorescent protein timers. Methods Mol. Biol. *1174*, 195–210.

Khmelinskii, A., Meurer, M., Duishoev, N., Delhomme, N., and Knop, M. (2011). Seamless gene tagging by endonuclease-driven homologous recombination. PLoS ONE *6*, e23794.

Khmelinskii, A., Keller, P.J., Bartosik, A., Meurer, M., Barry, J.D., Mardin, B.R., Kaufmann, A., Trautmann, S., Wachsmuth, M., Pereira, G., et al. (2012). Tandem fluorescent protein timers for in vivo analysis of protein dynamics. Nat. Biotechnol. *30*, 708–714.

Khmelinskii, A., Blaszczak, E., Pantazopoulou, M., Fischer, B., Omnus, D.J., Le Dez, G., Brossard, A., Gunnarsson, A., Barry, J.D., Meurer, M., et al. (2014). Protein quality control at the inner nuclear membrane. Nature *516*, 410–413.

Kim, H., Melén, K., Osterberg, M., and von Heijne, G. (2006). A global topology map of the Saccharomyces cerevisiae membrane proteome. Proc. Natl. Acad. Sci. USA *103*, 11142–11147.

Kim, H.-K., Kim, R.-R., Oh, J.-H., Cho, H., Varshavsky, A., and Hwang, C.-S. (2014). The N-terminal methionine of cellular proteins as a degradation signal. Cell *156*, 158–169.

Kleiger, G., and Mayor, T. (2014). Perilous journey: a tour of the ubiquitin-proteasome system. Trends Cell Biol. *24*, 352–359.

Knop, M., Siegers, K., Pereira, G., Zachariae, W., Winsor, B., Nasmyth, K., and Schiebel, E. (1999). Epitope tagging of yeast genes using a PCR-based strategy: more tags and improved practical routines. Yeast *15* (10B), 963–972.

Koren, I., Timms, R.T., Kula, T., Xu, Q., Li, M.Z., and Elledge, S.J. (2018). The eukaryotic proteome Is shaped by E3 ubiquitin ligases targeting C-terminal degrons. Cell *173*, 1622–1635.e14.

Kristensen, A.R., Gsponer, J., and Foster, L.J. (2013). Protein synthesis rate is the predominant regulator of protein expression during differentiation. Mol. Syst. Biol. *9*, 689.

Krogh, A., Larsson, B., von Heijne, G., and Sonnhammer, E.L.L. (2001). Predicting transmembrane protein topology with a hidden Markov model: application to complete genomes. J. Mol. Biol. *305*, 567–580.

Labbadia, J., and Morimoto, R.I. (2015). The biology of proteostasis in aging and disease. Annu. Rev. Biochem. *84*, 435–464.

Lammer, D., Mathias, N., Laplaza, J.M., Jiang, W., Liu, Y., Callis, J., Goebl, M., and Estelle, M. (1998). Modification of yeast Cdc53p by the ubiquitin-related protein rub1p affects function of the SCFCdc4 complex. Genes Dev. *12*, 914–926.

Lampert, F., Stafa, D., Goga, A., Soste, M.V., Gilberto, S., Olieric, N., Picotti, P., Stoffel, M., and Peter, M. (2018). The multi-subunit GID/CTLH E3 ubiquitin ligase promotes cell proliferation and targets the transcription factor Hbp1 for degradation. eLife *7*, e35528.

Leal-Esteban, L.C., Rothé, B., Fortier, S., Isenschmid, M., and Constam, D.B. (2018). Role of Bicaudal C1 in renal gluconeogenesis and its novel interaction with the CTLH complex. PLoS Genet. *14*, e1007487.

Leggett, D.S., Hanna, J., Borodovsky, A., Crosas, B., Schmidt, M., Baker, R.T., Walz, T., Ploegh, H., and Finley, D. (2002). Multiple associated proteins regulate proteasome structure and function. Mol. Cell *10*, 495–507.

Li, Z., Vizeacoumar, F.J., Bahr, S., Li, J., Warringer, J., Vizeacoumar, F.S., Min, R., Vandersluis, B., Bellay, J., Devit, M., et al. (2011). Systematic exploration of essential yeast gene function with temperature-sensitive mutants. Nat. Biotechnol. *29*, 361–367.

Li, G.-W., Burkhardt, D., Gross, C., and Weissman, J.S. (2014). Quantifying absolute protein synthesis rates reveals principles underlying allocation of cellular resources. Cell *157*, 624–635.

Liakopoulos, D., Doenges, G., Matuschewski, K., and Jentsch, S. (1998). A novel protein modification pathway related to the ubiquitin system. EMBO J. *17*, 2208–2214.

Lin, H.C., Yeh, C.W., Chen, Y.F., Lee, T.T., Hsieh, P.Y., Rusnac, D.V., Lin, S.Y., Elledge, S.J., Zheng, N., and Yen, H.S. (2018). C-terminal end-directed protein elimination by CRL2 ubiquitin ligases. Mol. Cell *70*, 602–613.e3.

Liu, H., and Pfirrmann, T. (2019). The Gid-complex: an emerging player in the ubiquitin ligase league. Biol. Chem. *400*, 1429–1441.

Liu, H., Ding, J., Köhnlein, K., Urban, N., Ori, A., Villavicencio-Lorini, P., Walentek, P., Klotz, L.O., Hollemann, T., and Pfirrmann, T. (2020). The GID ubiquitin ligase complex is a regulator of AMPK activity and organismal lifespan. Autophagy *16*, 1618–1634.

Lu, J., and Deutsch, C. (2008). Electrostatics in the ribosomal tunnel modulate chain elongation rates. J. Mol. Biol. *384*, 73–86.

Luo, W., Fang, H., and Green, N. (2006). Substrate specificity of inner membrane peptidase in yeast mitochondria. Mol. Genet. Genomics *275*, 431–436.

Matsuo, Y., Ikeuchi, K., Saeki, Y., Iwasaki, S., Schmidt, C., Udagawa, T., Sato, F., Tsuchiya, H., Becker, T., Tanaka, K., et al. (2017). Ubiquitination of stalled ribosome triggers ribosome-associated quality control. Nat. Commun. *8*, 159.

McShane, E., Sin, C., Zauber, H., Wells, J.N., Donnelly, N., Wang, X., Hou, J., Chen, W., Storchova, Z., Marsh, J.A., et al. (2016). Kinetic Analysis of Protein Stability Reveals Age-Dependent Degradation. Cell *167*, 803–815.e21.

Meissner, C., Lorenz, H., Weihofen, A., Selkoe, D.J., and Lemberg, M.K. (2011). The mitochondrial intramembrane protease PARL cleaves human Pink1 to regulate Pink1 trafficking. J. Neurochem. *117*, 856–867.

Melnykov, A., Chen, S.-J., and Varshavsky, A. (2019). Gid10 as an alternative N-recognin of the Pro/N-degron pathway. Proc. Natl. Acad. Sci. USA *116*, 15914–15923.

Menssen, R., Schweiggert, J., Schreiner, J., Kusevic, D., Reuther, J., Braun, B., and Wolf, D.H. (2012). Exploring the topology of the Gid complex, the E3 ubiquitin ligase involved in catabolite-induced degradation of gluconeogenic enzymes. J. Biol. Chem. *287*, 25602–25614.

Menssen, R., Bui, K., and Wolf, D.H. (2018). Regulation of the Gid ubiquitin ligase recognition subunit Gid4. FEBS Lett. *592*, 3286–3294.

Meurer, M., Duan, Y., Sass, E., Kats, I., Herbst, K., Buchmuller, B.C., Dederer, V., Huber, F., Kirrmaier, D., Štefl, M., et al. (2018). Genome-wide C-SWAT library for high-throughput yeast genome tagging. Nat. Methods *15*, 598–600.

Mevissen, T.E.T., and Komander, D. (2017). Mechanisms of Deubiquitinase Specificity and Regulation. Annu. Rev. Biochem. *86*, 159–192.

CellPress
OPEN ACCESS

Mitchell, A.L., Attwood, T.K., Babbitt, P.C., Blum, M., Bork, P., Bridge, A., Brown, S.D., Chang, H.Y., El-Gebali, S., Fraser, M.I., et al. (2019). InterPro in 2019: improving coverage, classification and access to protein sequence annotations. Nucleic Acids Res. *47* (D1), D351–D360.

Moerschell, R.P., Hosokawa, Y., Tsunasawa, S., and Sherman, F. (1990). The specificities of yeast methionine aminopeptidase and acetylation of amino-terminal methionine in vivo. Processing of altered iso-1-cytochromes c created by oligonucleotide transformation. J. Biol. Chem. *265*, 19638–19643.

Newman, J.R.S., Ghaemmaghami, S., Ihmels, J., Breslow, D.K., Noble, M., DeRisi, J.L., and Weissman, J.S. (2006). Single-cell proteomic analysis of S. cerevisiae reveals the architecture of biological noise. Nature *441*, 840–846.

Nikko, E., and Pelham, H.R.B. (2009). Arrestin-mediated endocytosis of yeast plasma membrane transporters. Traffic *10*, 1856–1867.

Oh, E., Akopian, D., and Rape, M. (2018). Principles of ubiquitin-dependent signaling. Annu. Rev. Cell Dev. Biol. *34*, 137–162.

Oughtred, R., Stark, C., Breitkreutz, B.J., Rust, J., Boucher, L., Chang, C., Kolas, N., O'Donnell, L., Leung, G., McAdam, R., et al. (2019). The BioGRID interaction database: 2019 update. Nucleic Acids Res. *47* (D1), D529–D541.

Özkaynak, E., Finley, D., and Varshavsky, A. (1984). The yeast ubiquitin gene: head-to-tail repeats encoding a polyubiquitin precursor protein. Nature *312*, 663–666.

Petersen, T.N., Brunak, S., von Heijne, G., and Nielsen, H. (2011). SignalP 4.0: discriminating signal peptides from transmembrane regions. Nat. Methods *8*, 785–786.

Pfirrmann, T., Villavicencio-Lorini, P., Subudhi, A.K., Menssen, R., Wolf, D.H., and Hollemann, T. (2015). RMND5 from Xenopus laevis is an E3 ubiquitin-ligase and functions in early embryonic forebrain development. PLoS ONE *10*, e0120342.

Pickart, C.M. (2001). Mechanisms underlying ubiquitination. Annu. Rev. Biochem. *70*, 503–533.

Qiao, S., Langlois, C.R., Chrustowicz, J., Sherpa, D., Karayel, O., Hansen, F.M., Beier, V., von Gronau, S., Bollschweiler, D., Schäfer, T., et al. (2020). Interconversion between Anticipatory and Active GID E3 Ubiquitin Ligase Conformations via Metabolically Driven Substrate Receptor Assembly. Mol. Cell *77*, 150–163.e9.

Rao, H., Uhlmann, F., Nasmyth, K., and Varshavsky, A. (2001). Degradation of a cohesin subunit by the N-end rule pathway is essential for chromosome stability. Nature *410*, 955–959.

Ravid, T., and Hochstrasser, M. (2008). Diversity of degradation signals in the ubiquitin-proteasome system. Nat. Rev. Mol. Cell Biol. *9*, 679–690.

Regelmann, J., Schüle, T., Josupeit, F.S., Horak, J., Rose, M., Entian, K.-D., Thumm, M., and Wolf, D.H. (2003). Catabolite degradation of fructose-1,6-bisphosphatase in the yeast Saccharomyces cerevisiae: a genome-wide screen identifies eight novel GID genes and indicates the existence of two degradation pathways. Mol. Biol. Cell *14*, 1652–1663.

Reggiori, F., and Pelham, H.R.B. (2002). A transmembrane ubiquitin ligase required to sort membrane proteins into multivesicular bodies. Nat. Cell Biol. *4*, 117–123.

Renz, C., Albanèse, V., Tröster, V., Albert, T.K., Santt, O., Jacobs, S.C., Khmelinskii, A., Léon, S., and Ulrich, H.D. (2020). Ubc13–Mms2 cooperates with a family of RING E3 proteins in budding yeast membrane protein sorting. J. Cell Sci. *133*, jcs.244566.

Ritchie, M.E., Phipson, B., Wu, D., Hu, Y., Law, C.W., Shi, W., and Smyth, G.K. (2015). limma powers differential expression analyses for RNA-sequencing and microarray studies. Nucleic Acids Res. *43*, e47.

Ruggiano, A., Foresti, O., and Carvalho, P. (2014). Quality control: ER-associated degradation: protein quality control and beyond. J. Cell Biol. *204*, 869–879.

Santt, O., Pfirrmann, T., Braun, B., Juretschke, J., Kimmig, P., Scheel, H., Hofmann, K., Thumm, M., and Wolf, D.H. (2008). The yeast GID complex, a novel ubiquitin ligase (E3) involved in the regulation of carbohydrate metabolism. Mol. Biol. Cell *19*, 3323–3333.

Schneider, C.A., Rasband, W.S., and Eliceiri, K.W. (2012). NIH Image to ImageJ: 25 years of image analysis. Nat. Methods *9*, 671–675.

Schwanhäusser, B., Busse, D., Li, N., Dittmar, G., Schuchhardt, J., Wolf, J., Chen, W., and Selbach, M. (2011). Global quantification of mammalian gene expression control. Nature *473*, 337–342.

Scott, D.C., Rhee, D.Y., Duda, D.M., Kelsall, I.R., Olszewski, J.L., Paulo, J.A., de Jong, A., Ovaa, H., Alpi, A.F., Harper, J.W., and Schulman, B.A. (2016). Two Distinct Types of E3 Ligases Work in Unison to Regulate Substrate Ubiquitylation. Cell *166*, 1198–1214.e24.

Sikorski, R.S., and Hieter, P. (1989). A system of shuttle vectors and yeast host strains designed for efficient manipulation of DNA in Saccharomyces cerevisiae. Genetics *122*, 19–27.

Singh, R.K., Gonzalez, M., Kabbaj, M.-H.M., and Gunjan, A. (2012). Novel E3 ubiquitin ligases that regulate histone protein levels in the budding yeast Saccharomyces cerevisiae. PLoS ONE *7*, e36295.

Sitron, C.S., Park, J.H., and Brandman, O. (2017). Asc1, Hel2, and Slh1 couple translation arrest to nascent chain degradation. RNA *23*, 798–810.

Subbotin, R.I., and Chait, B.T. (2014). A pipeline for determining protein-protein interactions and proximities in the cellular milieu. Mol. Cell. Proteomics *13*, 2824–2835.

Sugiyama, T., Li, S., Kato, M., Ikeuchi, K., Ichimura, A., Matsuo, Y., and Inada, T. (2019). Sequential Ubiquitination of Ribosomal Protein uS3 Triggers the Degradation of Non-functional 18S rRNA. Cell Rep. *26*, 3400–3415.e7.

Sundaramoorthy, E., Leonard, M., Mak, R., Liao, J., Fulzele, A., and Bennett, E.J. (2017). ZNF598 and RACK1 Regulate Mammalian Ribosome-Associated Quality Control Function by Mediating Regulatory 40S Ribosomal Ubiquitylation. Mol. Cell *65*, 751–760.e4.

Supek, F., Bošnjak, M., Škunca, N., and Šmuc, T. (2011). REVIGO summarizes and visualizes long lists of gene ontology terms. PLoS ONE *6*, e21800.

Taggart, J.C., and Li, G.W. (2018). Production of Protein-Complex Components Is Stoichiometric and Lacks General Feedback Regulation in Eukaryotes. Cell Syst. *7*, 580–589.e4.

Texier, Y., Toedt, G., Gorza, M., Mans, D.A., van Reeuwijk, J., Horn, N., Willer, J., Katsanis, N., Roepman, R., Gibson, T.J., et al. (2014). Elution profile analysis of SDS-induced subcomplexes by quantitative mass spectrometry. Mol. Cell. Proteomics *13*, 1382–1391.

Tong, A.H.Y., and Boone, C. (2007). High-throughput strain construction and systematic synthetic lethal screening in Saccharomyces cerevisiae. Methods Microbiol. *36*, 369–386, 706–707.

Tong, A.H., Evangelista, M., Parsons, A.B., Xu, H., Bader, G.D., Pagé, N., Robinson, M., Raghibizadeh, S., Hogue, C.W., Bussey, H., et al. (2001). Systematic genetic analysis with ordered arrays of yeast deletion mutants. Science *294*, 2364–2368.

van der Lee, R., Lang, B., Kruse, K., Gsponer, J., Sánchez de Groot, N., Huynen, M.A., Matouschek, A., Fuxreiter, M., and Babu, M.M. (2014). Intrinsically disordered segments affect protein half-life in the cell and during evolution. Cell Rep. *8*, 1832–1844.

Varland, S., Osberg, C., and Arnesen, T. (2015). N-terminal modifications of cellular proteins: The enzymes involved, their substrate specificities and biological effects. Proteomics *15*, 2385–2401.

Varshavsky, A. (2011). The N-end rule pathway and regulation by proteolysis. Protein Sci. *20*, 1298–1345.

Varshavsky, A. (2019). N-degron and C-degron pathways of protein degradation. Proc. Natl. Acad. Sci. USA *116*, 358–366.

Verma, R., Aravind, L., Oania, R., McDonald, W.H., Yates, J.R., 3rd, Koonin, E.V., and Deshaies, R.J. (2002). Role of Rpn11 metalloprotease in deubiquitination and degradation by the 26S proteasome. Science *298*, 611–615.

Wach, A., Brachat, A., Pöhlmann, R., and Philippsen, P. (1994). New heterologous modules for classical or PCR-based gene disruptions in Saccharomyces cerevisiae. Yeast *10*, 1793–1808.

Wapinski, I., Pfeffer, A., Friedman, N., and Regev, A. (2007). Natural history and evolutionary principles of gene duplication in fungi. Nature *449*, 54–61.

Waterhouse, A.M., Procter, J.B., Martin, D.M.A., Clamp, M., and Barton, G.J. (2009). Jalview Version 2–a multiple sequence alignment editor and analysis workbench. Bioinformatics *25*, 1189–1191.

Willems, A.R., Schwab, M., and Tyers, M. (2004). A hitchhiker's guide to the cullin ubiquitin ligases: SCF and its kin. Biochim. Biophys. Acta *1695*, 133–170.

Winz, M.L., Peil, L., Turowski, T.W., Rappsilber, J., and Tollervey, D. (2019). Molecular interactions between Hel2 and RNA supporting ribosome-associated quality control. Nat. Commun. *10*, 563.

Winzeler, E.A., Shoemaker, D.D., Astromoff, A., Liang, H., Anderson, K., Andre, B., Bangham, R., Benito, R., Boeke, J.D., Bussey, H., et al. (1999). Functional characterization of the S. cerevisiae genome by gene deletion and parallel analysis. Science *285*, 901–906.

Wolff, S., Weissman, J.S., and Dillin, A. (2014). Differential scales of protein quality control. Cell *157*, 52–64.

Yamano, K., and Youle, R.J. (2013). PINK1 is degraded through the N-end rule pathway. Autophagy *9*, 1758–1769.

Yang, X., Arines, F.M., Zhang, W., and Li, M. (2018). Sorting of a multi-subunit ubiquitin ligase complex in the endolysosome system. eLife *7*, e33116.

Yao, T., and Cohen, R.E. (2002). A cryptic protease couples deubiquitination and degradation by the proteasome. Nature *419*, 403–407.

Yu, H., Braun, P., Yildirim, M.A., Lemmens, I., Venkatesan, K., Sahalie, J., Hirozane-Kishikawa, T., Gebreab, F., Li, N., Simonis, N., et al. (2008). High-quality binary protein interaction map of the yeast interactome network. Science *322*, 104–110.

Zheng, N., and Shabek, N. (2017). Ubiquitin Ligases: Structure, Function, and Regulation. Annu. Rev. Biochem. *86*, 129–157.

CellPress
OPEN ACCESS

STAR★METHODS

KEY RESOURCES TABLE

REAGENT or RESOURCE	SOURCE	IDENTIFIER
Antibodies		
Mouse monoclonal anti-HA (clone 12CA5)	In-house	N/A
Mouse monoclonal anti-GFP (clones 7.1 and 13.1)	Sigma-Aldrich	Cat#11814460001; RRID: AB_390913
Rabbit Peroxidase anti-Peroxidase	Sigma-Aldrich	Cat#P1291; RRID: AB_1079562
Mouse monoclonal anti-Pgk1 (clone 22C5D8)	Thermo Fisher Scientific	Cat#459250; RRID: AB_2532235
Rabbit Peroxidase anti-Peroxidase	Dako	Cat#Z0113
Goat polyclonal anti-Mouse IgG, HRP-conjugated	Thermo Fisher Scientific	Cat#G-21040; RRID: AB_2536527
Goat polyclonal anti-Mouse IgG, HRP-conjugated	Dianova GmbH	Cat#115-035-003
Rabbit polyclonal anti-GFP	Abcam	Cat#ab290; RRID: AB_303395
Dynabeads M-280 Sheep Anti-Rabbit IgG	Thermo Fisher Scientific	Cat#11203D; RRID: AB_2783009
Chemicals, peptides, and recombinant proteins		
MG132	Enzo Life Sciences	Cat#BML-PI102-0025
Complete EDTA-free protease inhibitor cocktail	Sigma-Aldrich	Cat#4693159001
Critical commercial assays		
Pierce ECL Plus Western Blotting Substrate	Thermo Fisher Scientific	Cat#32132
Deposited data		
R vignette reproducing the analysis of the screens profiling the ubiquitin-proteasome system and containing the screen dataset	This study	https://heidata.uni-heidelberg.de/citation?persistentId=doi:10.11588/data/Q3TSLH
Unprocessed immunoblot images	This study	https://data.mendeley.com/datasets/rv8f9bp5b2
Experimental models: organisms/strains		
Saccharomyces cerevisiae strain BY4741: S288c MATa his3Δ1 leu2Δ0 met15Δ0 ura3Δ0	Brachmann et al., 1998	N/A
Saccharomyces cerevisiae strain Y8205: S288c MATalpha his3Δ1 leu2Δ0 met15Δ0 ura3Δ0 can1Δ::STE2pr-spHIS5 lyp1Δ::STE3pr-LEU2	Tong and Boone, 2007	N/A
Saccharomyces cerevisiae strain Y7092: S288c MATalpha his3Δ1 leu2Δ0 met15Δ0 ura3Δ0 can1Δ::STE2pr-SpHIS5 lyp1Δ	Tong and Boone, 2007	N/A
Saccharomyces cerevisiae strain BY4743: S288c MATa/alpha his3Δ1/his3Δ1 leu2Δ0/leu2Δ0 MET15/met15Δ0 LYS2/lys2Δ0 ura3Δ0/ura3Δ0	Brachmann et al., 1998	N/A
YMaM330: Y8205 leu2Δ::GAL1pr-I-SCEI-natNT2	Khmelinskii et al., 2014	N/A
tFT library: YMaM330 ORF-mCherry-SceIsite-SpCYC1term-ScURA3-SceIsite-mCherryΔN-sfGFP	Khmelinskii et al., 2014	N/A
UPS array: BY4741 orf::kanMX	This study	N/A
UPS array control_1: BY4741 his3Δ::kanMX	This study	N/A

(*Continued on next page*)

Continued

REAGENT or RESOURCE	SOURCE	IDENTIFIER
UPS array control_2: BY4741 ura3Δ::kanMX	This study	N/A
AK1230: BY4741 ubr1Δ::kanMX6	This study	N/A
AK1231: BY4741 nta1Δ::kanMX6	This study	N/A
AK1232: BY4741 ate1Δ::kanMX6	This study	N/A
AK1233: BY4741 rad6Δ::kanMX6	This study	N/A
YIK138: BY4741 MCR1-TAP-HisMX	Ghaemmaghami et al., 2003	N/A
YIK101: BY4741 MCR1-TAP-HisMX ubr1Δ::natNT2	This study	N/A
YIK174: BY4741 MCR1-TAP-HisMX ate1Δ::natNT2	This study	N/A
YLZY0093: BY4741 MCR1-TAP-HisMX pdr5Δ::natNT2	This study	N/A
YIK179: BY4741 MCR1-3HA-kanMX	This study	N/A
YIK182: BY4741 MCR1-3HA-kanMX ubr1Δ::natNT2	This study	N/A
YIK189: BY4741 MCR1-3HA-kanMX tom7Δ::hphNT1	This study	N/A
YIK190: BY4741 MCR1-3HA-kanMX ubr1Δ::natNT2 tom7Δ::hphNT1	This study	N/A
YIK191: BY4741 MCR1-3HA-kanMX tim11Δ::hphNT1	This study	N/A
YIK192: BY4741 MCR1-3HA-kanMX ubr1Δ::natNT2 tim11Δ::hphNT1	This study	N/A
YBB228: YMaM330 EFT1-mCherry-sfGFP	This study	N/A
AK1293: YMaM330 EFT1-mCherry-sfGFP hel1Δ::kanMX6	This study	N/A
AK1312: YMaM330 EFT1-mCherry-sfGFP yuh1Δ::kanMX6	This study	N/A
AK1294: YMaM330 EFT1-mCherry-sfGFP ubc12Δ::kanMX6	This study	N/A
AK1295: YMaM330 EFT1-mCherry-sfGFP rub1Δ::kanMX6	This study	N/A
AK1296: YMaM330 EFT1-mCherry-sfGFP hrt3Δ::kanMX6	This study	N/A
AK1311: YMaM330 EFT1-mCherry-sfGFP ubp10Δ::kanMX6	This study	N/A
AK1297: YMaM330 EFT1-mCherry-sfGFP nam7Δ::kanMX6	This study	N/A
AK1326: BY4741 hel1Δ::hphNT1	This study	N/A
AK1322: BY4741 yuh1Δ::hphNT1	This study	N/A
AK1327: BY4741 ubc12Δ::hphNT1	This study	N/A
AK1328: BY4741 rub1Δ::hphNT1	This study	N/A
AK1329: BY4741 hrt3Δ::hphNT1	This study	N/A
AK1321: BY4741 ubp10Δ::hphNT1	This study	N/A
AK1330: BY4741 nam7Δ::hphNT1	This study	N/A
YMaM1074: YMaM330 HSM3-mCherry-sfGFP	This study	N/A
AK1248: YMaM330 HSM3-mCherry-sfGFP gid1Δ::kanMX6	This study	N/A

(*Continued on next page*)

Continued

REAGENT or RESOURCE	SOURCE	IDENTIFIER
YMaM1110: YMaM330 HSM3-mCherry-sfGFP gid2Δ::kanMX6	This study	N/A
AK1249: YMaM330 HSM3-mCherry-sfGFP gid5Δ::kanMX6	This study	N/A
AK1250: YMaM330 HSM3-mCherry-sfGFP gid7Δ::kanMX6	This study	N/A
AK1251: YMaM330 HSM3-mCherry-sfGFP gid8Δ::kanMX6	This study	N/A
AK1252: YMaM330 HSM3-mCherry-sfGFP gid9Δ::kanMX6	This study	N/A
YMaM1079: YMaM330 GPM3-mCherry-sfGFP	This study	N/A
AK1254: YMaM330 GPM3-mCherry-sfGFP gid1Δ::kanMX6	This study	N/A
YMaM1115: YMaM330 GPM3-mCherry-sfGFP gid2Δ::kanMX6	This study	N/A
AK1255: YMaM330 GPM3-mCherry-sfGFP gid5Δ::kanMX6	This study	N/A
AK1256: YMaM330 GPM3-mCherry-sfGFP gid7Δ::kanMX6	This study	N/A
AK1257: YMaM330 GPM3-mCherry-sfGFP gid8Δ::kanMX6	This study	N/A
AK1258: YMaM330 GPM3-mCherry-sfGFP gid9Δ::kanMX6	This study	N/A
YMaM1080: YMaM330 CPA1-mCherry-sfGFP	This study	N/A
AK1260: YMaM330 CPA1-mCherry-sfGFP gid1Δ::kanMX6	This study	N/A
YMaM1116: YMaM330 CPA1-mCherry-sfGFP gid2Δ::kanMX6	This study	N/A
AK1261: YMaM330 CPA1-mCherry-sfGFP gid5Δ::kanMX6	This study	N/A
AK1262: YMaM330 CPA1-mCherry-sfGFP gid7Δ::kanMX6	This study	N/A
AK1263: YMaM330 CPA1-mCherry-sfGFP gid8Δ::kanMX6	This study	N/A
AK1264: YMaM330 CPA1-mCherry-sfGFP gid9Δ::kanMX6	This study	N/A
YMaM1082: YMaM330 PHM8-mCherry-sfGFP	This study	N/A
AK1265: YMaM330 PHM8-mCherry-sfGFP gid1Δ::kanMX6	This study	N/A
YMaM1118: YMaM330 PHM8-mCherry-sfGFP gid2Δ::kanMX6	This study	N/A
AK1266: YMaM330 PHM8-mCherry-sfGFP gid5Δ::kanMX6	This study	N/A
AK1267: YMaM330 PHM8-mCherry-sfGFP gid7Δ::kanMX6	This study	N/A
AK1268: YMaM330 PHM8-mCherry-sfGFP gid8Δ::kanMX6	This study	N/A
AK1269: YMaM330 PHM8-mCherry-sfGFP gid9Δ::kanMX6	This study	N/A

(*Continued on next page*)

Continued

REAGENT or RESOURCE	SOURCE	IDENTIFIER
YMaM1090: YMaM330 BLM10-mCherry-sfGFP	This study	N/A
AK1270: YMaM330 BLM10-mCherry-sfGFP gid1Δ::kanMX6	This study	N/A
YMaM1126: YMaM330 BLM10-mCherry-sfGFP gid2Δ::kanMX6	This study	N/A
AK1271: YMaM330 BLM10-mCherry-sfGFP gid5Δ::kanMX6	This study	N/A
AK1272: YMaM330 BLM10-mCherry-sfGFP gid7Δ::kanMX6	This study	N/A
AK1273: YMaM330 BLM10-mCherry-sfGFP gid8Δ::kanMX6	This study	N/A
AK1274: YMaM330 BLM10-mCherry-sfGFP gid9Δ::kanMX6	This study	N/A
YMaM1091: YMaM330 MDH2-m Cherry-sfGFP	This study	N/A
AK1276: YMaM330 MDH2-mCherry-sfGFP gid1Δ::kanMX6	This study	N/A
YMaM1127: YMaM330 MDH2-mCherry-sfGFP gid2Δ::kanMX6	This study	N/A
AK1277: YMaM330 MDH2-mCherry-sfGFP gid5Δ::kanMX6	This study	N/A
AK1278: YMaM330 MDH2-mCherry-sfGFP gid7Δ::kanMX6	This study	N/A
AK1279: YMaM330 MDH2-mCherry-sfGFP gid8Δ::kanMX6	This study	N/A
AK1280: YMaM330 MDH2-mCherry-sfGFP gid9Δ::kanMX6	This study	N/A
YMaM1094: YMaM330 YOR283W-m Cherry-sfGFP	This study	N/A
AK1281: YMaM330 YOR283W-mCherry-sfGFP gid1Δ::kanMX6	This study	N/A
YMaM1130: YMaM330 YOR283W-m Cherry-sfGFP gid2Δ::kanMX6	This study	N/A
AK1282: YMaM330 YOR283W-mCherry-sfGFP gid5Δ::kanMX6	This study	N/A
AK1283: YMaM330 YOR283W-mCherry-sfGFP gid7Δ::kanMX6	This study	N/A
AK1284: YMaM330 YOR283W-mCherry-sfGFP gid8Δ::kanMX6	This study	N/A
AK1285: YMaM330 YOR283W-mCherry-sfGFP gid9Δ::kanMX6	This study	N/A
AK1253: YMaM330 HSM3-mCherry-sfGFP gid4Δ::kanMX6	This study	N/A
YKEK067: YMaM330 HSM3-mCherry-sfGFP gid10Δ::kanMX6	This study	N/A
YKEK084: YMaM330 HSM3-mCherry-sfGFP ylr149cΔ::kanMX6	This study	N/A
AK1259: YMaM330 GPM3-mCherry-sfGFP gid4Δ::kanMX6	This study	N/A
YKEK068: YMaM330 GPM3-mCherry-sfGFP gid10Δ::kanMX6	This study	N/A

(*Continued on next page*)

CellPress
OPEN ACCESS

Continued

REAGENT or RESOURCE	SOURCE	IDENTIFIER
YKEK085: YMaM330 GPM3-mCherry-sfGFP ylr149cΔ::kanMX6	This study	N/A
YMaM1145: YMaM330 CPA1-mCherry-sfGFP gid4Δ::kanMX6	This study	N/A
YKEK069: YMaM330 CPA1-mCherry-sfGFP gid10Δ::kanMX6	This study	N/A
AK1335: YMaM330 CPA1-mCherry-sfGFP ylr149cΔ::hphNT1	This study	N/A
AK1338: YMaM330 CPA1-mCherry-sfGFP gid2Δ::kanMX6 ylr149cΔ::hphNT1	This study	N/A
YMaM1146: YMaM330 PHM8-mCherry-sfGFP gid4Δ::kanMX6	This study	N/A
YKEK070: YMaM330 PHM8-mCherry-sfGFP gid10Δ::kanMX6	This study	N/A
AK1336: YMaM330 PHM8-mCherry-sfGFP ylr149cΔ::hphNT1	This study	N/A
AK1339: YMaM330 PHM8-mCherry-sfGFP gid2Δ::kanMX6 ylr149cΔ::hphNT1	This study	N/A
AK1275: YMaM330 BLM10-mCherry-sfGFP gid4Δ::kanMX6	This study	N/A
YKEK071: YMaM330 BLM10-mCherry-sfGFP gid10Δ::kanMX6	This study	N/A
YKEK088: YMaM330 BLM10-mCherry-sfGFP ylr149cΔ::kanMX6	This study	N/A
YMaM1148: YMaM330 MDH2-mCherry-sfGFP gid4Δ::kanMX6	This study	N/A
YKEK072: YMaM330 MDH2-mCherry-sfGFP gid10Δ::kanMX6	This study	N/A
AK1337: YMaM330 MDH2-mCherry-sfGFP ylr149cΔ::hphNT1	This study	N/A
AK1340: YMaM330 MDH2-mCherry-sfGFP gid2Δ::kanMX6 ylr149cΔ::hphNT1	This study	N/A
AK1286: YMaM330 YOR283W-mCherry-sfGFP gid4Δ::kanMX6	This study	N/A
YKEK073: YMaM330 YOR283W-mCherry-sfGFP gid10Δ::kanMX6	This study	N/A
YKEK090: YMaM330 YOR283W-mCherry-sfGFP ylr149cΔ::kanMX6	This study	N/A
AK1240: BY4741 gid2Δ::kanMX6	This study	N/A
AK1241: BY4741 gid9Δ::kanMX6	This study	N/A
AK1243: BY4741 gid4Δ::kanMX6	This study	N/A
AK1245: BY4741 moh1Δ::kanMX6	This study	N/A
AK1244: BY4741 ipf1Δ::kanMX6	This study	N/A
AK1242: BY4741 ubc8Δ::kanMX6	This study	N/A
YMaM1205: Y7092 can1Δ::STE3pr-LEU2-GAL1pr-NLS-I-SCEI	Meurer et al., 2018	N/A
YJJF0017: Y7092 can1Δ::STE3pr-SpHIS5-TEFterm-GAL1pr-NLS-I-SCEI	This study	N/A
YLZY0017: YJJF0017 CPA1-hph	This study	N/A
YLZY0002: YJJF0017 CPA1-mCherry-sfGFP-hph	This study	N/A

(*Continued on next page*)

Continued

REAGENT or RESOURCE	SOURCE	IDENTIFIER
YLZY0007: YJJF0017 CPA1-mCherry-sfGFP-hph gid2Δ::natNT2	This study	N/A
YLZY0006: YJJF0017 CPA1-mCherry-sfGFP-hph ubp14Δ::natNT2	This study	N/A
YLZY0013: YJJF0017 CPA1-mCherry-sfGFP-hph gid2Δ::natNT2 ubp14Δ::kanMX6	This study	N/A
YLZY0018: YJJF0017 PHM8-hph	This study	N/A
YLZY0003: YJJF0017 PHM8-mCherry-sfGFP-hph	This study	N/A
YLZY0009: YJJF0017 PHM8-mCherry-sfGFP-hph gid2Δ::natNT2	This study	N/A
YLZY0008: YJJF0017 PHM8-mCherry-sfGFP-hph ubp14Δ::natNT2	This study	N/A
YLZY0015: YJJF0017 PHM8-mCherry-sfGFP-hph gid2Δ::natNT2 ubp14Δ::kanMX6	This study	N/A
CPA1_tFT library: YMaM330 CPA1-mCherry-SceIsite-SpCYC1term-ScURA3-SceIsite-mCherryΔN-sfGFP	Khmelinskii et al., 2014	N/A
PHM8_tFT library: YMaM330 PHM8-mCherry-SceIsite-SpCYC1term-ScURA3-SceIsite-mCherryΔN-sfGFP	Khmelinskii et al., 2014	N/A
MDH2_tFT library: YMaM330 MDH2-mCherry-SceIsite-SpCYC1term-ScURA3-SceIsite-mCherryΔN-sfGFP	Khmelinskii et al., 2014	N/A
KO library: BY4743 ORF/orfΔ::kanMX	Winzeler et al., 1999	N/A
YKEK114: YMaM330 gid9Δ::kanMX	This study	N/A
YKEK115: YMaM330 gid4Δ::kanMX	This study	N/A
YKEK075: YMaM330 gid10Δ::kanMX	This study	N/A
YKEK092: YMaM330 ylr149cΔ::kanMX	This study	N/A
YKEK146: YMaM330 YLR149C-3HA-kanMX4	This study	N/A
YLZY0089: YMaM330 gid5Δ::hphNT1	This study	N/A
YMaM1083: YMaM330 GID1-mCherry-sfGFP	This study	N/A
YLZY0091: YMaM330 GID1-mCherry-sfGFP gid5Δ::hphNT1	This study	N/A
YMaM1075: YMaM330 GID8-mCherry-sfGFP	This study	N/A
YMaM1085: YMaM330 GID5-mCherry-sfGFP	This study	N/A
YMaM1088: YMaM330 GID7-mCherry-sfGFP	This study	N/A
YMaM1100: YMaM330 UBC8-mCherry-sfGFP	This study	N/A
YKEK148: YMaM330 CPA1(S2A)-mCherry-sfGFP	This study	N/A
YKEK149: YMaM330 CPA1(S2G)-mCherry-sfGFP	This study	N/A
YKEK151: YMaM330 CPA1(S2A)-mCherry-sfGFP gid9Δ::kanMX6	This study	N/A

(*Continued on next page*)

Continued

REAGENT or RESOURCE	SOURCE	IDENTIFIER
YKEK152: YMaM330 CPA1(S2G)-mCherry-sfGFP gid9Δ::kanMX6	This study	N/A
YKEK160: YMaM330 CPA1(S2A)-mCherry-sfGFP ylr149cΔ::hphNT1	This study	N/A
YKEK161: YMaM330 CPA1(S2G)-mCherry-sfGFP ylr149cΔ::hphNT1	This study	N/A
YKEK196: YMaM330 BLM10(T2A)-mCherry-sfGFP	This study	N/A
YKEK197: YMaM330 BLM10(T2G)-mCherry-sfGFP	This study	N/A
YKEK199: YMaM330 BLM10(T2A)-mCherry-sfGFP gid9Δ::kanMX6	This study	N/A
YKEK200: YMaM330 BLM10(T2G)-mCherry-sfGFP gid9Δ::kanMX6	This study	N/A
YKEK202: YMaM330 BLM10(T2A)-mCherry-sfGFP ylr149cΔ::hphNT1	This study	N/A
YKEK203: YMaM330 BLM10(T2G)-mCherry-sfGFP ylr149cΔ::hphNT1	This study	N/A
GID_hits array: YMaM330 ORF-mCherry-SceIsite-SpCYC1term-ScURA3-SceIsite-mCherryΔN-sfGFP	This study	N/A
YKEK178: BY4741 gid10Δ::kanMX6	This study	N/A
YKEK179: BY4741 ylr149cΔ::kanMX6	This study	N/A
Recombinant DNA		
pFA6a-kanMX6	Wach et al., 1994	N/A
pFA6a-hphNT1	Janke et al., 2004	Euroscarf: P30347
pFA6a-natNT2	Janke et al., 2004	Euroscarf: P30346
pYM1: pFA6a-3HA-kanMX4	Knop et al., 1999	N/A
pRS413-GPDpr-MDH2-mCherry-sfGFP	This study	N/A
pRS413-GPDpr-MDH2(P2A)-mCherry-sfGFP	This study	N/A
pRS413-GPDpr-MDH2(P2G)-mCherry-sfGFP	This study	N/A
pRS413-GPDpr-PHM8-mCherry-sfGFP	This study	N/A
pRS413-GPDpr-PHM8(T2A)-mCherry-sfGFP	This study	N/A
pRS413-GPDpr-PHM8(T2G)-mCherry-sfGFP	This study	N/A
pRS413-GPDpr	This study	N/A
pRS413-GPDpr-YLR149C	This study	N/A
pRS413-GPDpr-HA-YLR149C	This study	N/A
pRS413-GPDpr-HA-YLR149C(Δ727-730)	This study	N/A
pRS413-GPDpr-HA-YLR149C(Y727A L729A)	This study	N/A
pRS413-GPDpr-HA-YLR149C(Y727D L729D)	This study	N/A
pRS316	Sikorski and Hieter, 1989	N/A
pRS316-GID5	This study	N/A
pRS316-GID5(W606A H610A Y613A)	This study	N/A
pRS316-GID5(W606A Y613A Q649A)	This study	N/A
pRS413-GPDpr-GPM3-mCherry-sfGFP	This study	N/A

(*Continued on next page*)

Continued		
REAGENT or RESOURCE	SOURCE	IDENTIFIER
pRS413-GPDpr-GPM3(T2A)-mCherry-sfGFP	This study	N/A
pRS413-GPDpr-GPM3(T2G)-mCherry-sfGFP	This study	N/A
pRS413-GPDpr-YOR283W-mCherry-sfGFP	This study	N/A
pRS413-GPDpr-YOR283W(T2A)-mCherry-sfGFP	This study	N/A
pRS413-GPDpr-YOR283W(T2G)-mCherry-sfGFP	This study	N/A
pRS413-GPDpr-HA-Luc	This study	N/A
pRS413-GPDpr-HA-Luc-GID11^{C20}	This study	N/A
pRS413-GPDpr-HA-Luc-GID4^{C20}	This study	N/A
pRS413-GPDpr-PHM8(HSM3$^{2-5}$)-mCherry-sfGFP	This study	N/A
pRS413-GPDpr-PHM8(GPM3$^{2-5}$)-mCherry-sfGFP	This study	N/A
pRS413-GPDpr-PHM8(CPA1$^{2-5}$)-mCherry-sfGFP	This study	N/A
pRS413-GPDpr-PHM8(BLM10$^{2-5}$)-mCherry-sfGFP	This study	N/A
pRS413-GPDpr-PHM8(MDH2$^{2-5}$)-mCherry-sfGFP	This study	N/A
pRS413-GPDpr-PHM8(YOR283W$^{2-5}$)-mCherry-sfGFP	This study	N/A
Software and algorithms		
ImageJ	Schneider et al., 2012	https://imagej.nih.gov/ij/
ImageLab	Bio-Rad	http://www.bio-rad.com/en-us/product/image-lab-software?ID=KRE6P5E8Z
R	R Foundation for Statistical Computing	https://www.R-project.org/
Phobius	Käll et al., 2004	N/A
TMHMM	Krogh et al., 2001	N/A
InterPro database	Mitchell et al., 2019	N/A
SignalP	Petersen et al., 2011	N/A
MUSCLE	Edgar, 2004	https://www.ebi.ac.uk/Tools/msa/muscle/
JalView	Waterhouse et al., 2009	https://www.jalview.org/
Revigo	Supek et al., 2011	http://revigo.irb.hr/

RESOURCE AVAILABILITY

Lead contact

Information and requests for resources and reagents should be directed to the Lead Contact, Anton Khmelinskii (a.khmelinskii@imb-mainz.de).

Materials availability

All unique/stable reagents generated in this study are available from the Lead Contact with a completed Materials Transfer Agreement.

Data and code availability

The R vignette reproducing the analysis of the screens profiling the ubiquitin-proteasome system and containing the screen dataset is deposited in the heiData repository:

https://heidata.uni-heidelberg.de/citation?persistentId=doi:10.11588/data/Q3TSLH

Unprocessed immunoblot images are available at the Mendeley Data repository:

https://data.mendeley.com/datasets/rv8f9bp5b2

EXPERIMENTAL MODEL AND SUBJECT DETAILS

All yeast strains used in this study are listed in the Key resources table and are derivatives of BY4741, Y8205, Y7092 or BY4743. Yeast genome manipulations (gene tagging and gene deletion) were performed using PCR targeting and lithium acetate transformation (Janke et al., 2004). All experiments were performed at 30°C in synthetic complete (SC) medium with 2% (w/v) glucose as carbon source, unless stated otherwise.

METHOD DETAILS

Immunoblotting

(i) For log phase experiments with *MCR1-TAP* and *MCR1-3xHA* strains, cells were grown to $6x10^6$-$1x10^7$ cells/ml in SC medium with 2% (w/v) glucose. 1 mL samples were mixed with 150 μL of 1.85 M NaOH and 10 μL of 2-mercaptoethanol and flash-frozen in liquid nitrogen.

(ii) For glucose starvation experiments with *MCR1-TAP* and *MCR1-3xHA* strains, cells from a dense pre-culture were inoculated into SC medium with 0.1% (w/v) glucose to a density of $1x10^6$ cells/ml and grown for 48 h to $4x10^7$ cells/ml. 250 μL samples were processed as above.

(iii) For proteasome inhibition experiments with the *MCR1-TAP pdr5Δ* strain, log phase cultures were treated with MG132 (BML-PI102-0025, Enzo Life Sciences) to 80 μg/ml final concentration or DMSO as control for 90 min, followed by cell harvesting as above.

(iv) For conditional analysis of Gid11-HA expression, cells were first grown to $8x10^6$-$1x10^7$ cells/ml in SC medium with 2% (w/v) glucose. Then, 10% of the culture was harvested as control, while the remaining was washed once with water and resuspended in growth media with different compositions: (1) carbon starvation medium (SC without carbon sources), (2) SC with 2% (v/v) ethanol, (3) SC with 2% (w/v) glucose, (4) SC with 2% (v/v) glycerol, (5) amino acid starvation medium (SC with 2% (w/v) glucose but without amino acids), (6) nitrogen starvation medium (SC with 2% (w/v) glucose but without nitrogen base), (7) osmotic stress medium (SC with 2% (w/v) glucose and 1.4 M NaCl) and (8) SC with 2% (w/v) glucose and 1 M KH_2PO_4. Cells were incubated at 30°C (also 37°C for cells resuspended in SC glucose) for 3 h before harvesting. In addition, yeast colonies were harvested from an agar plate incubated at 30°C for 48 h.

For (i) and (ii), samples were thawed on ice and whole cell protein extracts were prepared by precipitation with 150 μL of 55% (w/v) of trichloroacetic acid, followed by centrifugation to remove the supernatant. The pellet was resuspended in 50-100 μL of HU buffer (8 M urea, 5% SDS, 200 mM Tris-HCl pH 6.8, 1 mM EDTA, 1.5% DTT and phenol blue as coloring and pH indicator) per $1x10^7$ cells (Knop et al., 1999), followed by SDS-PAGE and immunoblotting. For TAP-tagged strains, membranes were probed with rabbit peroxidase anti-peroxidase (PAP) antibodies (Z0113, Dako). For HA-tagged strains, membranes were probed with mouse anti-HA antibodies (12CA5) and HRP-conjugated goat anti-mouse antibodies (Dianova 115-035-003). Membranes were imaged on a LAS-4000 system (Fuji). Quantification was performed in ImageJ (Schneider et al., 2012).

For (iii) and (iv), samples were processed as above, followed by SDS-PAGE and immunoblotting. Membranes were probed with rabbit peroxidase anti-peroxidase (PAP) antibodies (P1291, Sigma-Aldrich) to detect Mcr1-TAP, or with mouse anti-HA antibodies (12CA5) followed by HRP-conjugated anti-mouse antibodies (G-21040, Thermo Fisher Scientific) to detect Gid11-HA. The loading control Pgk1 was detected using mouse anti-Pgk1 antibodies (459250, Thermo Fisher Scientific) followed by the same anti-mouse secondary antibodies. The ChemiDoc MP imaging system (Bio-Rad) was used to image the membranes after addition of the Pierce ECL Plus Western Blotting Substrate (32132, Thermo Fisher Scientific). Quantification was performed using ImageLab (Bio-Rad).

Flow cytometry

Strains were grown to saturation in 96-well plates, diluted into fresh medium, and grown for 8 h to 2-8 × 10^6 cells/ml. Fluorescence measurements were performed on a BD FACSCanto RUO (BD Biosciences) equipped with a high-throughput sampler loader, a 488-nm laser with a combination of 505 nm long-pass and 530/30 nm band pass emission filters for sfGFP detection and a 561 nm laser with a combination of 600 nm long-pass and 610/20 nm band pass emission filters for mCherry detection. Populations were gated for single cells in the G1 phase of the cell cycle using the first peak in the side scatter width (SSC-W) histogram. At least 13000 cells were analyzed for each strain. Median intensities of cellular autofluorescence were subtracted from each channel and the mCherry/sfGFP ratio was calculated.

Measurements of proteome abundance and turnover with the tFT library

The tFT library, taken through marker excision in the tagged loci (Khmelinskii et al., 2011), was arranged in 1536-colony format using a pinning robot (RoToR, Singer Instruments), with 4 technical replicates of each tFT-tagged strain next to replicates of an untagged control strain, dummy colonies on the outer rows and columns to minimize the influence of nutrient access on colony size and fluorescence and, on each plate, a set of reference strains spanning the full range of protein abundances and stabilities in the tFT library.

Fluorescence intensities of the final colonies were measured after 24 h of growth using Infinite M1000 or Infinite M1000 Pro plate readers (Tecan) equipped with stackers for automated plate loading (Tecan) and custom temperature control chambers. Measurements in mCherry (587/10 nm excitation, 610/10 nm emission, optimal detector gain) and sfGFP (488/10 nm excitation, 510/10 nm emission, optimal detector gain) channels were performed at 400 Hz frequency of the flash lamp, with ten flashes averaged for each measurement.

After removing measurements of border colonies, mCherry and sfGFP fluorescence intensities were corrected for spatial effects by robust local regression using measurements of the control strain, corrected for cellular autofluorescence by subtracting the median of the control strain on each plate, log-transformed and scaled across plates by the median of the reference strains on each plate. Technical replicates were summarized by taking the median. Distribution of fluorescence intensities and mCherry/sfGFP ratios were median-centered.

For the gene ontology analysis, all proteins tagged in the tFT library were mapped to GO Slim terms. GO terms with 200 or fewer proteins were subsequently removed. In addition, GO terms with high variability in $\log_2$(mCherry/sfGFP) were excluded. For unbiased identification of GO terms with high variability, the dependence of $\log_2$(mCherry/sfGFP) median absolute deviation (MAD) on median $\log_2$(mCherry/sfGFP) per term was removed by linear regression, and terms with corrected MAD($\log_2$(mCherry/sfGFP)) above an arbitrary threshold of 0.1 were subsequently removed. The result was filtered for redundant GO Slim terms and visualized using Revigo (Supek et al., 2011).

Profiling of the ubiquitin-proteasome system

Each strain in the tFT library (Khmelinskii et al., 2014) was crossed to the UPS array (Table S2) using synthetic genetic array (SGA) methodology (Baryshnikova et al., 2010b; Tong et al., 2001). Crosses were performed in 1536-colony format, with 4 technical replicates placed next to each other. Screens were conducted in batches of 192 tFT queries. Two queries (*UBI4-tFT* and *YJR096W-tFT*) were repeated in every batch (Figure S2A). Each screen plate consisted of two queries crossed to the UPS array and a set of strains spanning the full range of protein abundances and stabilities in the tFT library, used as a reference across all plates. Mating, sporulation, selection of haploids carrying both a tFT-tagged allele and a mutant allele, followed by marker excision in the tFT-tagged locus were performed by sequential pinning of yeast colonies on appropriate selective media using pinning robots (BioMatrix, S&P Robotics) (Baryshnikova et al., 2010b; Khmelinskii et al., 2011). Plates were photographed to determine colony sizes. Fluorescence intensities of the final colonies were measured after 24 h of growth at 30°C as detailed above. This temperature was chosen to only partially inhibit growth of all temperature-sensitive mutants. Measurements were filtered for failed crosses based on colony size after haploid selection. Fluorescence intensity measurements were log-transformed and median effects for each tFT query were subtracted. Spatial effects on plates were corrected by local regression. The UPS array contained two negative control strains (*ura3Δ::kanMX* and *his3Δ::kanMX*; Table S2). Absolute fluorescence intensities of all tFT queries in the wild-type background were scaled across plates using the reference strains before calculating mCherry/sfGFP ratios. For each tFT query, corrected sfGFP and mCherry intensities in each mutant were compared to the mean of negative controls. For each mutant, the dependence of changes in fluorescence on absolute fluorescence intensity and screen order was removed by a local polynomial fit. The same correction was applied to mCherry/sfGFP ratios. Finally, a moderated t test implemented in the R/Bioconductor package limma (Ritchie et al., 2015) was used to test for interaction effects and to compute p values, adjusted for multiple testing using the method of Benjamini-Hochberg.

Stability measurements of individual proteins in low throughput (Figures 3C, 5C, 5D, 5G, S6G, S6H, S7B–S7D, S7I, and S7J) were performed with strains constructed independently (Key resources table) or obtained through independent crosses using identical procedures on a pinning robot (RoToR, Singer Instruments). For each tFT-tagged protein, fluorescence intensities of colonies were corrected for autofluorescence, using measurements from neighboring negative controls, and normalized for plate effects, using measurements from neighboring wild-type colonies.

Genome-wide screens for factors affecting protein stability

tFT query strains (*MDH2-tFT*, *CPA1-tFT* and *PHM8-tFT*, Key resources table) were crossed with a heterozygous diploid genome-wide library of yeast gene deletion mutants (Winzeler et al., 1999). Crosses were performed in 1536-colony format, with 4 technical replicates of each cross arranged next to each other. Mating, sporulation, selection of haploids carrying both a tFT-tagged allele and a gene deletion, followed by marker excision in the tFT-tagged locus were performed by sequential pinning of yeast colonies on appropriate selective media using a pinning robot (RoToR, Singer Instruments) (Baryshnikova et al., 2010b; Khmelinskii et al., 2011). Plates were photographed to determine colony sizes. Fluorescence intensities of the final colonies were measured after 24 h of growth as detailed above. Measurements of colony size after haploid selection, corrected for spatial effects by local regression, were used to identify and remove failed crosses or measurements from empty positions on the plates. Fluorescence intensity measurements were log-transformed and corrected for spatial effects before calculating mCherry/sfGFP ratios. For each query, changes in protein abundance (sfGFP intensity) and stability (mCherry/sfGFP ratio) were estimated by calculating z-scores (Dederer et al., 2019). Technical replicates were summarized by calculating the mean and standard deviation. P values were computed using a t test and adjusted for multiple testing using the method of Benjamini-Hochberg.

Analysis of Gid11 conservation

Gid11 homologs in different yeast species were obtained from Huerta-Cepas et al. (2014) and Wapinski et al. (2007) or identified by sequence homology to *S. cerevisiae* Gid11 (Table S7). The topology of the evolutionary tree was adapted from Dujon (2010). Multiple sequence alignment of putative Gid11 sequences was performed using MUSCLE (Edgar, 2004) and a histogram of alignment conservation was calculated with JalView (Waterhouse et al., 2009).

Co-immunoprecipitation

Strains were grown in 100 mL SC medium lacking histidine with 2% (w/v) glucose to $\sim 1\times10^7$ cells/ml. Cells were harvested by centrifugation and lysed in 600 μL of IP buffer (50 mM Tris-HCl pH 7.5, 100 mM NaCl, 1% (v/v) Triton X-100, 2 mM EDTA and protease inhibitors (4693159001, Sigma-Aldrich)) by vortexing at 4°C in the presence of acid-washed glass beads. Lysates were centrifuged at 4°C for 10 min and 400 μL of supernatant was collected, of which 300 μL was then added to 60 μL of Dynabeads M-280 Sheep Anti-Rabbit IgG (11203D, Thermo Fisher Scientific) previously conjugated with 1 μL of rabbit anti-GFP antibodies (ab290, Abcam). Immunoprecipitation of tFT-tagged proteins was allowed to proceed for 2 h at 4°C with gentle rocking. After that, beads were washed three times with 1 mL of IP buffer, resuspended in 50 μL of IP buffer plus 17 μL of 4X Laemmli SDS sample buffer (250 mM Tris-HCl pH 6.8, 8% (w/v) SDS, 40% (v/v) glycerol, 5% (v/v) 2-mercaptoethanol, and 0.1% (w/v) bromophenol blue), and incubated at 99°C for 10 min, followed by SDS-PAGE and immunoblotting as described above. Input samples were prepared by mixing 30 μL of lysates with 20 μL of IP buffer and 17 μL of 4X Laemmli SDS sample buffer and processed similarly. Membranes were probed with mouse anti-GFP antibodies (11814460001, Sigma-Aldrich) followed by HRP-conjugated anti-mouse antibodies (G-21040, Thermo Fisher Scientific) to detect tFT-tagged proteins, or with mouse anti-HA antibodies (12CA5) followed by the same secondary antibodies to detect the presence of any co-immunoprecipitated HA-tagged proteins.

QUANTIFICATION AND STATISTICAL ANALYSIS

Quantification and statistical analysis procedures are detailed for every experiment in Method details. Bar plots throughout the manuscript represent mean ± standard deviation ($n \geq 3$).

Molecular Cell, Volume *81*

Supplemental information

Timer-based proteomic profiling

# of the ubiquitin-proteasome system reveals

a substrate receptor of the GID ubiquitin ligase

Ka-Yiu Edwin Kong, Bernd Fischer, Matthias Meurer, Ilia Kats, Zhaoyan Li, Frank Rühle, Joseph D. Barry, Daniel Kirrmaier, Veronika Chevyreva, Bryan-Joseph San Luis, Michael Costanzo, Wolfgang Huber, Brenda J. Andrews, Charles Boone, Michael Knop, and Anton Khmelinskii

Figure S1

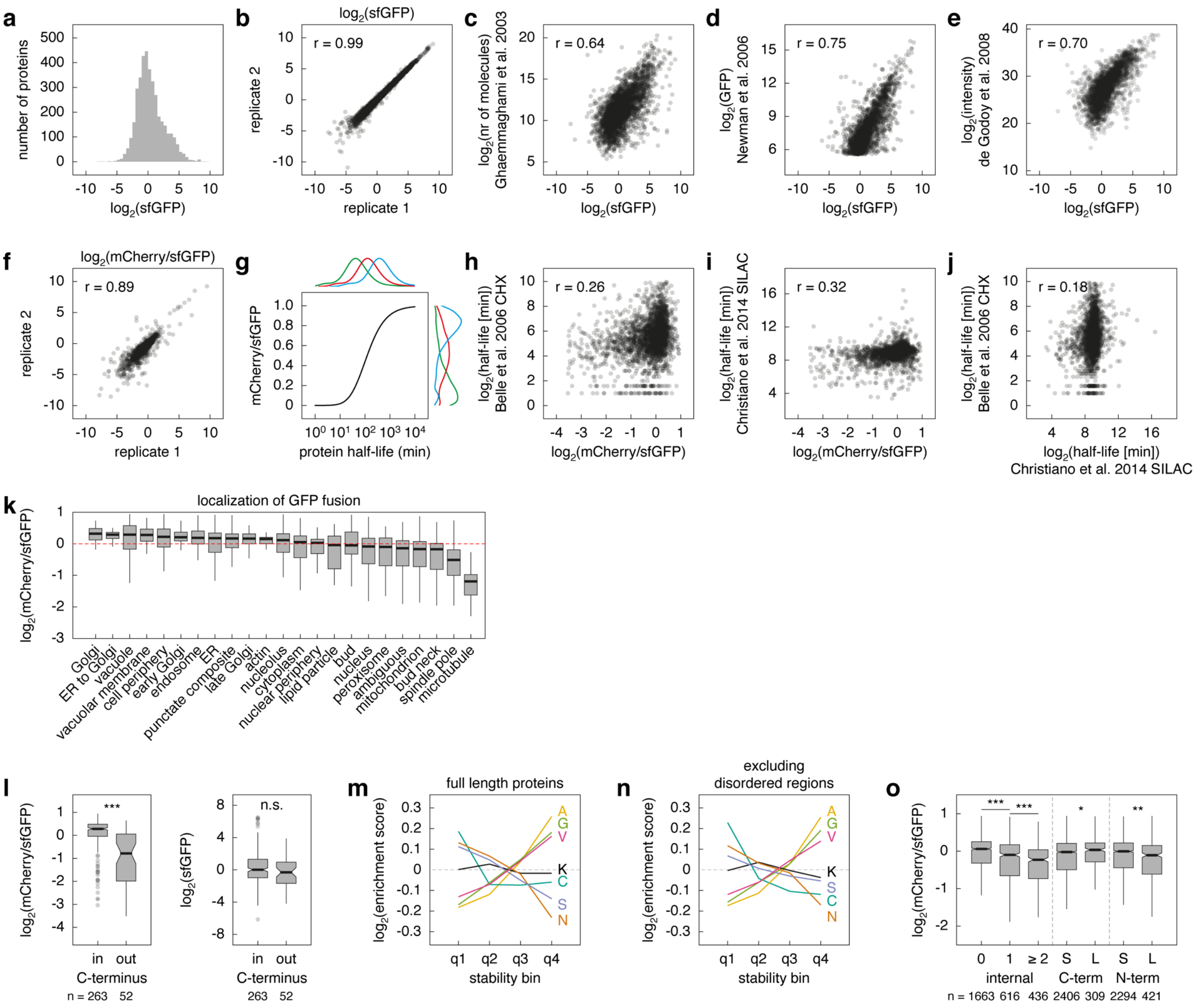

Figure S1. Measurements of proteome abundance and turnover with the tFT library, related to Figure 1

a – Distribution of protein abundance in the yeast proteome, determined using the tFT library. sfGFP fluorescence measurements of 4004 strains expressing different proteins C-terminally tagged with the mCherry-sfGFP timer at their endogenous chromosomal loci, grown on agar medium with 2 biological replicates each with 4 technical replicates per protein, summarized by their median value (Table S1).

b – Comparison of sfGFP fluorescence intensities between two biological replicates of the tFT library.

c, **d**, **e** – Comparison between protein abundance estimates with the tFT library and protein abundance measurements by (**c**) immunoblotting of strains expressing TAP-tagged proteins (Ghaemmaghami et al., 2003), (**d**) flow cytometry of strains expressing GFP-tagged proteins (Newman et al., 2006) and (**e**) mass spectrometry (de Godoy et al., 2008).

f – Comparison of protein stability estimates between two biological replicates of the tFT library.

g – Theoretical relationship between the mCherry/sfGFP ratio and protein half-life calculated using experimentally determined maturation parameters of mCherry and sfGFP (Khmelinskii et al., 2012). To assess if the non-linear dependence of the mCherry/sfGFP ratio on protein half-life could affect the distribution of mCherry/sfGFP ratios in the tFT library, distributions of mCherry/sfGFP ratios (right) were calculated for three distributions of protein half-lives (top, red curve – distribution of protein half-lives in *S. cerevisiae* experimentally determined by cycloheximide chases of strains expressing TAP-tagged proteins, with an average half-life of ~43 min (Belle et al., 2006), green and blue – shifts of the red curve towards faster or slower average protein turnover, respectively). The distribution of mCherry/sfGFP ratios in the tFT library could be skewed towards low mCherry/sfGFP ratios if the average half-life of yeast proteins is significantly higher than ~43 min (blue curves).

h, **i** – Comparison between protein stability estimates with the tFT library and (**h**) protein stability measurements by cycloheximide chase of strains expressing TAP-tagged proteins (Belle et al., 2006) or (**i**) using pulse-SILAC mass spectrometry (Christiano et al., 2014).

j – Comparison between protein stability measurements by cycloheximide chase of strains expressing TAP-tagged proteins (Ghaemmaghami et al., 2003) and measurements using pulse-SILAC mass spectrometry (Christiano et al., 2014).

k – mCherry/sfGFP ratios of proteins with different subcellular localizations, determined by fluorescence microscopy of strains expressing GFP-tagged proteins (Huh et al., 2003). Dashed line marks the median of mCherry/sfGFP ratios in the tFT library.
l – mCherry/sfGFP ratios (left) and sfGFP intensities (right) of secretory proteins with the C-terminus located in the cytosol (in) or in the ER lumen (out) (Kim et al., 2006). ***, p-value < 0.001; n.s., not significant in a Wilcoxon rank-sum test. Differences in local environment between ER lumen and cytosol likely contribute to the observed difference in mCherry/sfGFP ratios between the two groups of proteins.
m, n – Enrichment scores of the indicated amino acids (amino acid frequency in a selected group relative to the whole tFT library) for proteins in the tFT library split into four quantiles according to their mCherry/sfGFP ratios (q1 – lowest stability, q4 – highest stability), as indicated in Fig. 1b. Full protein sequences (**m**) or sequences excluding long disordered segments (**n**) retrieved from (van der Lee et al., 2014) were considered.
o – mCherry/sfGFP ratios of proteins with different levels of disorder: 0, 1 or ≥ 2 internal disordered segments (defined as continuous stretches of ≥ 40 disordered residues), long (L) or short (S) disordered stretches (defined as stretches of ≤ 30 or > 30 disordered residues, respectively) at the N- or at the C-terminus (van der Lee et al., 2014). C-terminal disordered stretches considered here are in the context of native untagged proteins. Upon C-terminal tagging with the tFT, these disordered stretches become internal. *, **, ***, p-value < 0.05, 0.01, 0.001 in a Wilcoxon rank-sum test, respectively.

Figure S2

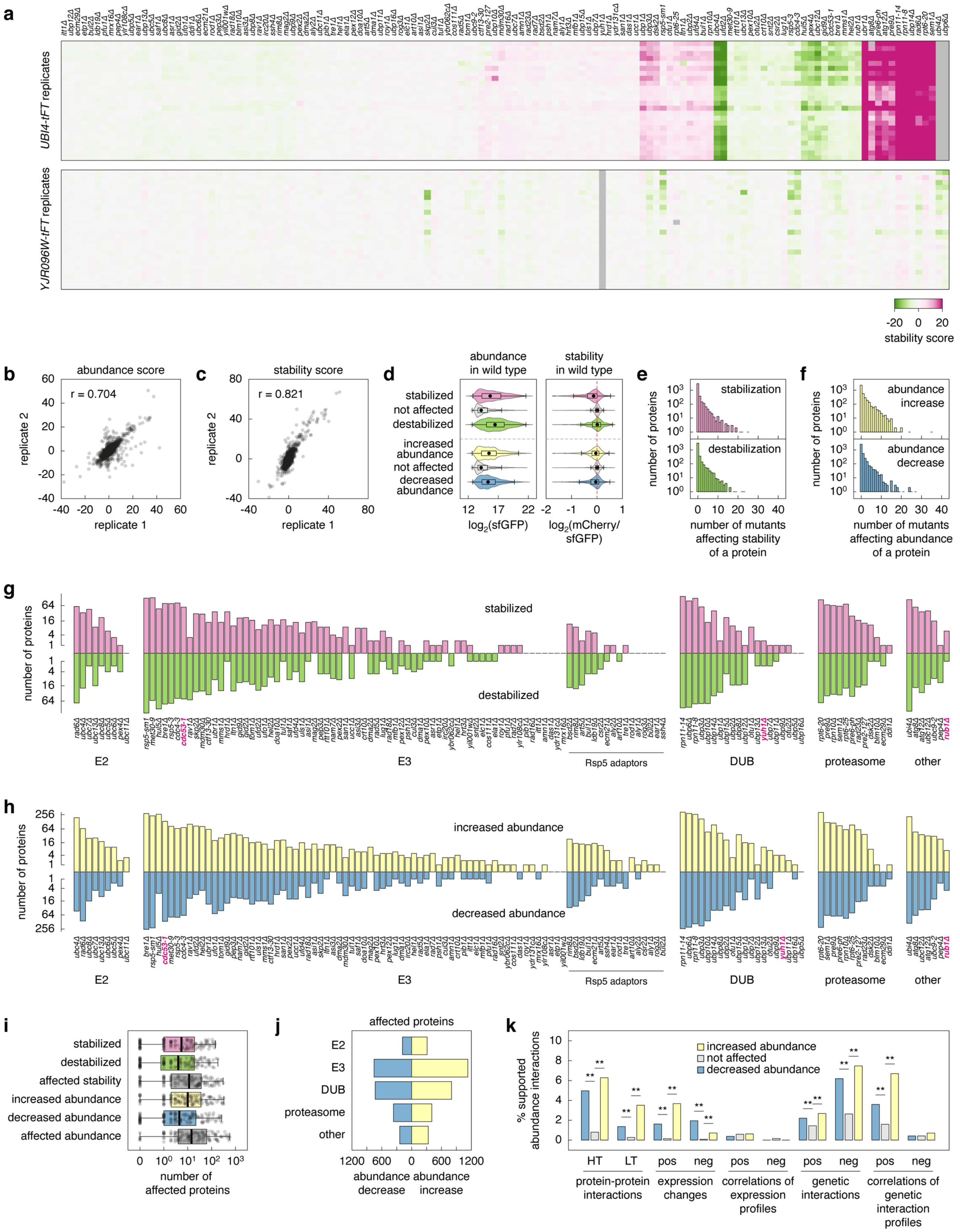

Figure S2. Overview of tFT-tagged proteins affected in mutants of UPS components, related to Figure 2

a – Reproducibility of screens to functionally profile the ubiquitin-proteasome system. Scaled changes in mCherry/sfGFP ratios (stability score) for two tFT queries, *UBI4-tFT* (top) and *YJR096W-tFT* (bottom), across all tested UPS mutants, assayed 24 times throughout the screens.

b, **c** – Reproducibility of screens to functionally profile the ubiquitin-proteasome system. Comparison of scaled changes in sfGFP intensities (abundance score, **b**) and mCherry/sfGFP ratios (stability score, **c**) for 96 tFT-tagged proteins that were independently screened against the UPS array in two biological replicates. Each data point represents the mean of 4 technical replicates.
d – Distributions of protein abundance (sfGFP levels) and stability (mCherry/sfGFP intensity ratio) of tFT-tagged proteins in the wild type background. Proteins were grouped according to their behavior in terms of abundance or stability in mutants of ubiquitin-proteasome system components. Proteins were considered to be significantly affected in terms of abundance or stability for mutant-tFT interactions with an absolute abundance or stability score > 4 at 1% false discovery rate (**d**-**j**).
e, **f** – Distributions of the number of mutants affecting stability (**e**) or abundance (**f**) of a protein for the 3806 tested tFT queries.
g, **h** – Number of proteins affected in terms of stability (**g**) or abundance (**h**) in the 132 mutants in the UPS array. *cdc53-1*, *yuh1*Δ, *rub1*Δ strains and mutants of Rsp5 adaptors are highlighted for clarity.
i – Box plot of the number of proteins affected in terms of stability or abundance in the 132 mutants in the UPS array. Centerlines mark the medians, box limits indicate the 25th and 75th percentiles, and whiskers extend to minimum and maximum values.
j – Distribution of the total number of proteins that increased or decreased in abundance in mutants of ubiquitin-conjugating enzymes (E2), ubiquitin-protein ligases (E3), deubiquitinating enzymes (DUB), proteasomal components (proteasome) or other factors (Table S2).
k – Overlap between abundance interactions and different types of interactions obtained from external datasets (STAR Methods). Abundance interactions were grouped according to impact of the mutant on the abundance of the tFT-tagged protein at 1% false discovery rate. pos – positive, neg – negative; **, p-value < 0.01 in a Fisher's exact test.

Figure S3

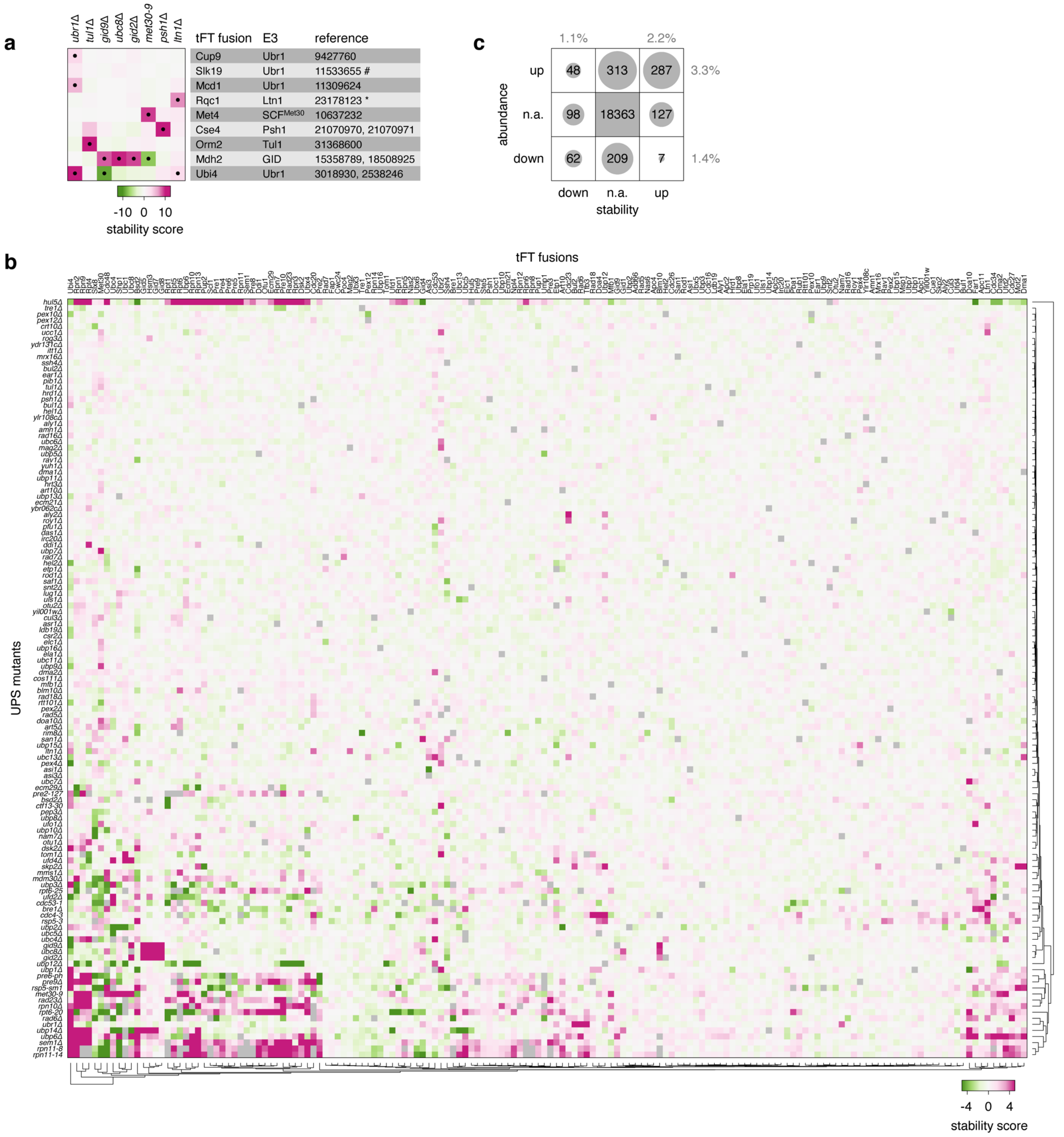

Figure S3. Self-regulation in the UPS, related to Figure 2

a – Heatmap of protein stability changes for known substrates of various ubiquitin-protein ligases (E3) in the indicated UPS mutants. Stability scores are color-coded from green (stability decrease) to magenta (stability increase). Significant changes in protein stability at 1% false discovery rate are marked (●). For each substrate, the cognate E3 and the PubMed IDs of the studies describing the enzyme-substrate relationship are listed.
#, Slk19 was shown to not be a Ubr1 substrate. *, Rqc1 was suggested to be a potential Ltn1 substrate.
b – Heatmap of protein stability changes for tFT-tagged UPS components in the indicated UPS mutants. Stability scores are color-coded from green (stability decrease) to magenta (stability increase).
c – Summary of phenotypic outcomes in terms of protein abundance and stability for tFT-tagged UPS components across all tested mutant-tFT pairs at 1% false discovery rate. n.a. – protein abundance or stability not affected. The percentage of mutant-tFT pairs with each phenotype is indicated.

Figure S4

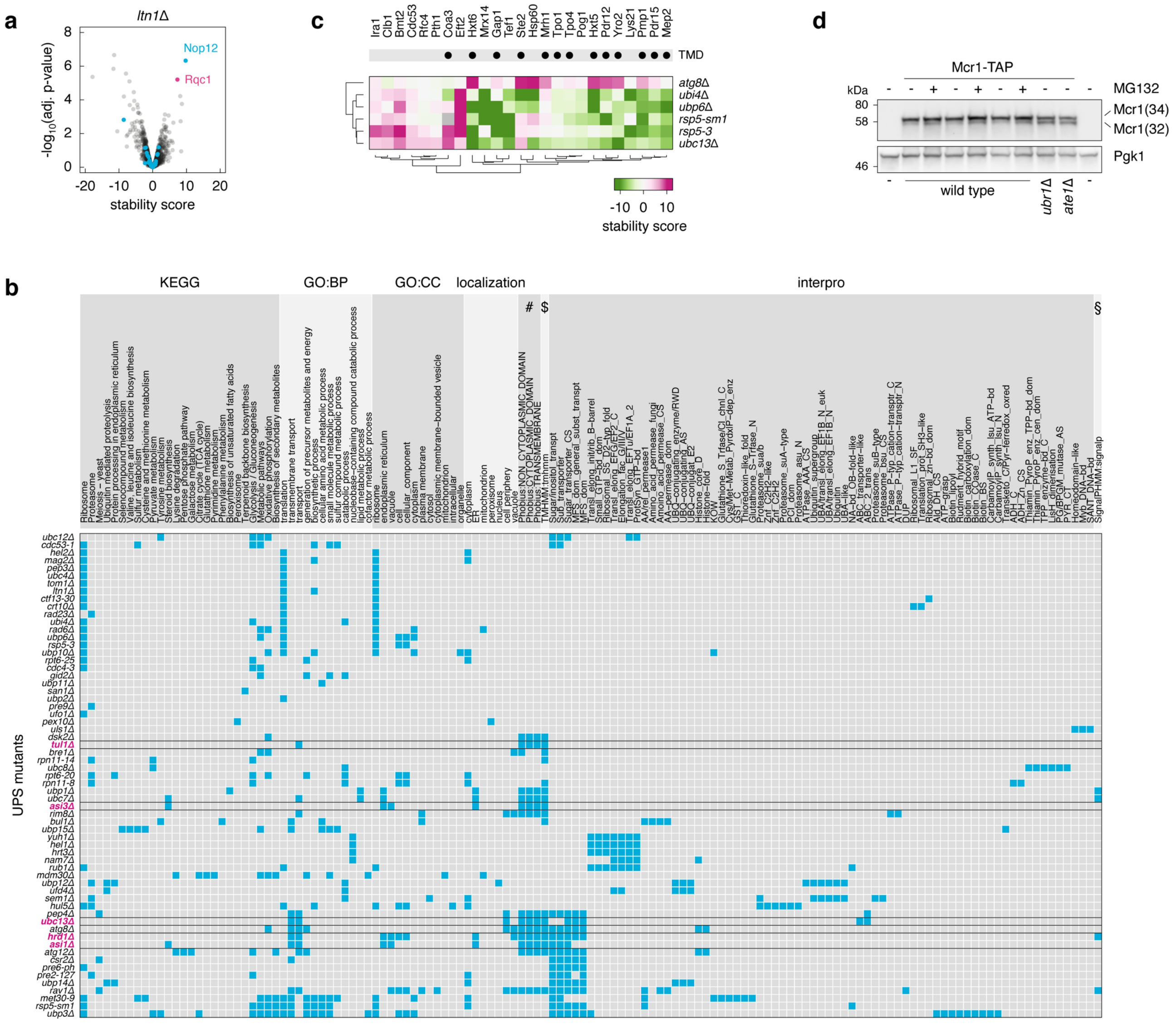

Figure S4. Turnover interactions inform on UPS functions, related to Figures 2 and 3

a – Volcano plot of changes in protein stability in the absence of the E3 Ltn1. Proteins containing a strong polybasic stretch (Brandman et al., 2012) are highlighted in blue.

b – Summary of gene set enrichment analysis for proteins with altered stability in UPS mutants. Blue – proteins in a given category are overrepresented among those affected by a given mutant, gray – no overrepresentation. KEGG – pathways in the Kyoto Encyclopedia of Genes and Genomes; GO:BP – biological process GO terms; GO Slim CC – cellular component GO terms; localization – protein localization as determined by microscopy of strains expressing GFP-tagged proteins (Huh et al., 2003); #, $ – proteins with predicted transmembrane domains using Phobius (Käll et al., 2004) or TMHMM (Krogh et al., 2001); interpro – protein families or domains in the InterPro database (Mitchell et al., 2019); § – proteins with predicted signals peptides using SignalP (Petersen et al., 2011). Only mutants with at least one significant association were included in the heatmap. Profiles of *tul1*Δ, *asi3*Δ, *ubc13*Δ, *hrd1*Δ and *asi1*Δ mutants are highlighted for clarity.

c – Summary heatmap of protein stability changes in the absence of the Ubc13 ubiquitin-conjugating enzyme. Stability scores are color-coded from green (stability decrease) to magenta (stability increase). Only proteins with a significant change in stability in the *ubc13*Δ mutant (1% false discovery rate and absolute stability score > 4) are shown. Proteins with transmembrane domains (TMDs) are indicated. Included for comparison are mutants with phenotypes correlated to that of *ubc13*Δ (Fig. 4a): positive correlation – *ubi4*Δ, *ubp6*Δ, *rsp5-sm1*, *rsp5-3* and negative correlation – *atg8*Δ. Because sfGFP has a higher pKa value than mCherry, the mCherry-sfGFP timer is pH-sensitive and the mCherry/sfGFP ratio increases with decreasing pH (Khmelinskii and Knop, 2014). Therefore, for transmembrane proteins degraded in the vacuole, e.g., Gap1, blocking protein degradation can result in a decrease in the mCherry/sfGFP ratio.

d – Accumulation of Mcr1(32) upon proteasome inhibition with MG132 for 90 min. Pgk1 was used as loading control.

Figure S5

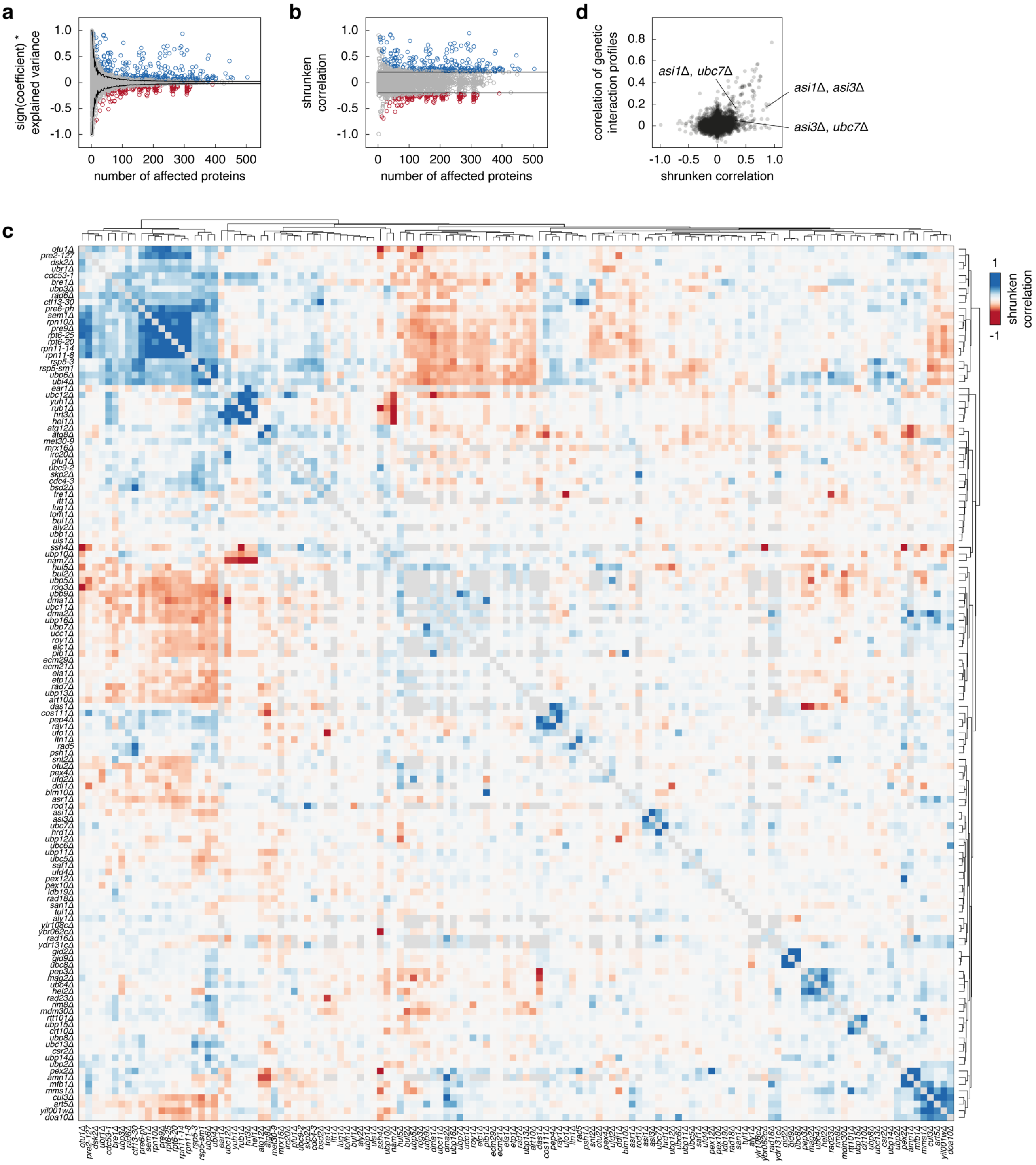

Figure S5. Correlation analysis of proteome turnover profiles, related to Figure 4

a, b – Calculation of correlations of protein turnover profiles. For each mutant, tFT-tagged proteins with significantly altered stability are selected. To assess if proteome turnover profiles of two mutants are correlated, a linear model is fitted on the union of sets of proteins affected in each mutant and p-values are adjusted by the method of Benjamini-Hochberg that controls for the false discovery rate. The explained variance is then used as a measure for the goodness-of-fit. Since a different number of proteins is used for each correlation, pairs of mutants with a small number of affected proteins can achieve a high value of explained variance while the p-value is still poor (**a**). Therefore, the explained variance was corrected for the number of affected proteins used in the test to obtain a shrunken correlation used hereafter (**b**) (STAR Methods). Red – significant negative correlations, blue – significant positive correlations (1% false discovery rate). The explained variance of negatively correlated pairs of mutants is multiplied by -1.

c – Annotated heatmap of shrunken correlations of proteome turnover profiles for all tested mutants in the UPS array.

d – Comparison of correlations of genetic interaction profiles between genes in the UPS array and shrunken correlations of proteome turnover profiles from **c**. For genetic interaction profiles, correlations are typically considered significant for Pearson correlation coefficients > 0.2 (Costanzo et al., 2016). Absolute shrunken correlation coefficients < 0.2 are not significant (**b**). Significant negative and some positive shrunken correlations are not supported by correlations of genetic interaction profiles, as exemplified by correlations between *asi1*Δ, *asi3*Δ and *ubc7*Δ mutants.

Figure S6

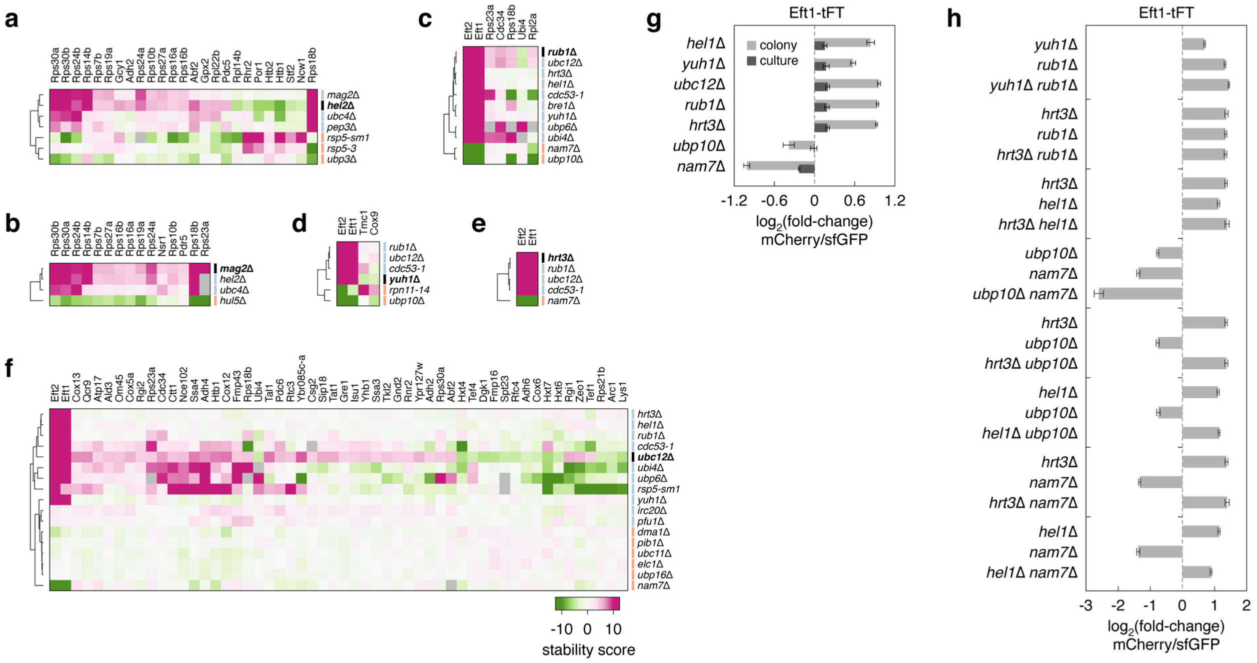

Figure S6. Protein turnover changes underlying correlations of proteome turnover profiles, related to Figure 4

a-f – Summary heatmaps of protein stability changes in the absence of the indicated components of the ubiquitin-proteasome system. Stability scores are color-coded from green (stability decrease) to magenta (stability increase). In each heatmap, only proteins with a significant change in stability in the mutant highlighted in bold (1% false discovery rate and absolute stability score > 4) are shown and their behavior in mutants with correlated phenotypes (blue – mutants with positive correlation of proteome turnover profile, orange – negative correlation) is included for comparison.

g, h – Differences in mCherry/sfGFP ratios between the indicated mutants and wild type strains expressing Eft1-tFT (mean ± s.d., $n = 4$). Comparison between measurements of log phase cultures by flow cytometry and plate reader measurements of colonies (**g**); plate reader measurements of colonies (**h**).

Figure S7

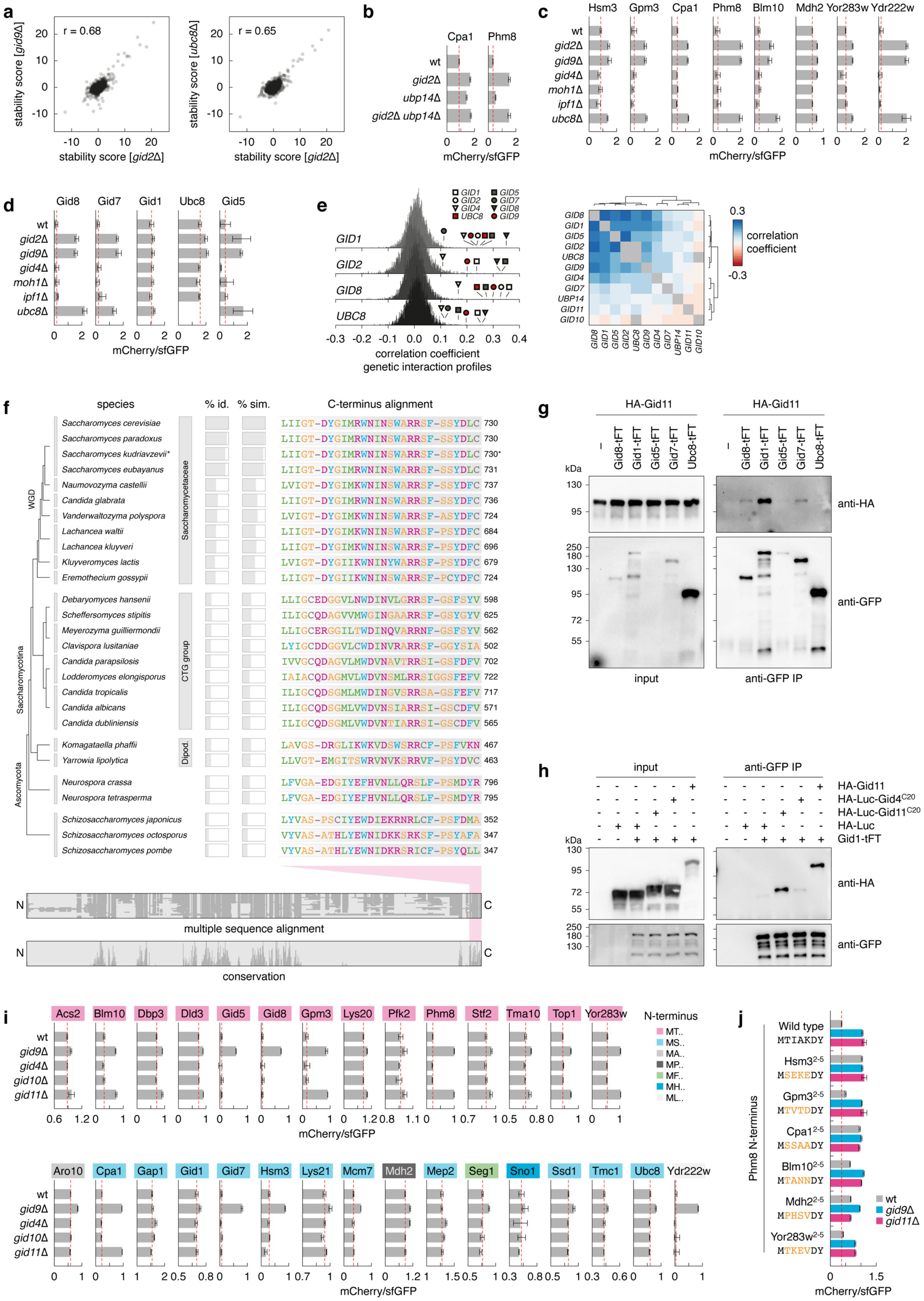
a
r = 0.68
stability score [gid9Δ]
stability score [gid2Δ]
r = 0.65
stability score [ubc8Δ]
stability score [gid2Δ]
b
Cpa1
Phm8
wt
gid2Δ
ubp14Δ
gid2Δ ubp14Δ
mCherry/sfGFP
c
Hsm3
Gpm3
Cpa1
Phm8
Blm10
Mdh2
Yor283w
Ydr222w
wt
gid2Δ
gid9Δ
gid4Δ
moh1Δ
ipf1Δ
ubc8Δ
mCherry/sfGFP
d
Gid8
Gid7
Gid1
Ubc8
Gid5
mCherry/sfGFP
e
GID1
GID2
GID8
UBC8
correlation coefficient
genetic interaction profiles
correlation coefficient
f
species
% id.
% sim.
C-terminus alignment
Saccharomyces cerevisiae
Saccharomyces paradoxus
Saccharomyces kudriavzevii*
Saccharomyces eubayanus
Naumovozyma castellii
Candida glabrata
Vanderwaltozyma polyspora
Lachancea waltii
Lachancea kluyveri
Kluyveromyces lactis
Eremothecium gossypii
Debaryomyces hansenii
Scheffersomyces stipitis
Meyerozyma guilliermondii
Clavispora lusitaniae
Candida parapsilosis
Lodderomyces elongisporus
Candida tropicalis
Candida albicans
Candida dubliniensis
Komagataella phaffii
Yarrowia lipolytica
Neurospora crassa
Neurospora tetrasperma
Schizosaccharomyces japonicus
Schizosaccharomyces octosporus
Schizosaccharomyces pombe
WGD
Saccharomycetaceae
CTG group
Dipod.
Saccharomycotina
Ascomycota
multiple sequence alignment
conservation
g
HA-Gid11
Gid8-tFT
Gid1-tFT
Gid5-tFT
Gid7-tFT
Ubc8-tFT
kDa
anti-HA
anti-GFP
input
anti-GFP IP
h
input
anti-GFP IP
HA-Gid11
HA-Luc-Gid4C20
HA-Luc-Gid11C20
HA-Luc
Gid1-tFT
anti-HA
anti-GFP
i
Acs2
Blm10
Dbp3
Dld3
Gid5
Gid8
Gpm3
Lys20
Pfk2
Phm8
Stf2
Tma10
Top1
Yor283w
N-terminus
MT..
MS..
MA..
MP..
MF..
MH..
ML..
wt
gid9Δ
gid4Δ
gid10Δ
gid11Δ
mCherry/sfGFP
Aro10
Cpa1
Gap1
Gid1
Gid7
Hsm3
Lys21
Mcm7
Mdh2
Mep2
Seg1
Sno1
Ssd1
Tmc1
Ubc8
Ydr222w
mCherry/sfGFP
j
Wild type
MTIAKDY
Hsm3 2-5
MSEKEDY
Gpm3 2-5
MTVTDDY
Cpa1 2-5
MSSAADY
Blm10 2-5
MTANNDY
Mdh2 2-5
MPHSVDY
Yor283w 2-5
MTKEVDY
Phm8 N-terminus
wt
gid9Δ
gid11Δ
mCherry/sfGFP

Figure S7. Analysis of protein turnover by the GID complex, related to Figures 5 and 6

a – Comparison of changes in mCherry/sfGFP ratios (stability score) in strains lacking *GID2*, *GID9* or *UBC8* for the 3806 tested tFT queries.

b-d – mCherry/sfGFP ratios of colonies expressing tFT-tagged proteins and carrying the indicated mutations (mean ± s.d., *n* = 4).

e – Histograms of Pearson correlation coefficients (PCC) calculated between the genetic interaction profiles of the indicated GID genes and 75% of all yeast genes, obtained from a previously published genome-scale genetic interaction map (Costanzo et al., 2016) (left). Correlating GID genes (PCC > 0.1) are marked on the plots. Heatmap of Pearson correlation coefficients calculated between the genetic interaction profiles of all GID genes (right).

f – Gid11 conservation across yeasts. Left – putative Gid11 homologs in different yeast species (Table S7). The topology of the evolutionary tree was adapted from (Dujon, 2010). The branch lengths are arbitrary. WGD – whole-genome duplication, Dipod. – Dipodascaceae. Percentage of sequence identity and similarity (% id. and % sim., respectively) relative to *S. cerevisiae* Gid11 were calculated. *, the annotated Gid11 sequence of *Saccharomyces kudriavzevii* is 640 residues long, shorter than those of closely related species. This appears to be due to a premature stop codon caused by a single nucleotide deletion in codon 631. Here we reverted this single nucleotide deletion, which results in a protein 730 residues long. Bottom – multiple sequence alignment of putative Gid11 sequences and histogram of alignment conservation. Sequences were ordered following the evolutionary tree, from *S. cerevisiae* Gid11 (top) to *S. pombe* Gid11 (bottom). Gaps in the multiple sequence alignment are indicated in light gray. Right – C-terminal portion of the multiple sequence alignment, highlighting the C-terminal Φ[D/E]ΦX motif.

g – Co-immunoprecipitation analysis of the interaction between overexpressed HA-Gid11 and chromosomally tFT-tagged GID subunits or the ubiquitin-conjugating enzyme Ubc8.

h – Co-immunoprecipitation analysis of the interaction between endogenously tagged Gid1-tFT and HA-luciferase (HA-Luc), HA-Luc-Gid4^{C20} or HA-Luc-Gid11^{C20} (HA-Luc fused to the C-terminal 20 residues of Gid4 or Gid11, respectively). A strain with overexpressed HA-Gid11 was included for comparison.

i – mCherry/sfGFP ratios of colonies expressing tFT-tagged potential GID substrates (Fig. 5a) and lacking GID components (mean ± s.d., *n* = 3). Proteins are color-coded according to the identity of the residue after the initiator methionine. Note that for some tFT fusions the x-axis does not start at zero to clearly visualize the small but reproducible effects of *gid* mutants.

j – mCherry/sfGFP ratios of colonies expressing tFT-tagged Phm8 variants with different N-termini (mean ± s.d., *n* = 4). The sequence of each N-terminus and the potential GID substrate from which the first four residues after the initiator methionine were derived are indicated.
